# Black soldier fly larvae (*Hermetia illucens*) strengthen the metabolic function of food waste biodegradation by gut microbiome

**DOI:** 10.1111/1751-7915.13393

**Published:** 2019-03-18

**Authors:** Cheng‐Liang Jiang, Wei‐Zheng Jin, Xin‐Hua Tao, Qian Zhang, Jun Zhu, Shi‐Yun Feng, Xin‐Hua Xu, Hong‐Yi Li, Ze‐Hua Wang, Zhi‐Jian Zhang

**Affiliations:** ^1^ College of Environmental and Resource Sciences ZheJiang University HangZhou 310058 China; ^2^ HangZhou GuSheng Biotechnology Co. Ltd HangZhou 311108 China; ^3^ Department of Biological and Agricultural Engineering University of Arkansas Fayetteville AR 72701 USA; ^4^ College of Agriculture and Biotechnology ZheJiang University HangZhou 310058 China

## Abstract

Vermicomposting using black soldier fly (BSF) larvae (*Hermetia illucens*) has gradually become a promising biotechnology for waste management, but knowledge about the larvae gut microbiome is sparse. In this study, 16S rRNA sequencing, SourceTracker, and network analysis were leveraged to decipher the influence of larvae gut microbiome on food waste (FW) biodegradation. The microbial community structure of BSF vermicompost (BC) changed greatly after larvae inoculation, with a peak colonization traceable to gut bacteria of 66.0%. The relative abundance of 11 out of 21 metabolic function groups in BC were significantly higher than that in natural composting (NC), such as carbohydrate‐active enzymes. In addition, 36.5% of the functional genes in BC were significantly higher than those in NC. The changes of metabolic functions and functional genes were significantly correlated with the microbial succession. Moreover, the bacteria that proliferated in vermicompost, including *Corynebacterium*,* Vagococcus*, and *Providencia*, had strong metabolic abilities. Systematic and complex interactions between the BSF gut and BC bacteria occurred over time through invasion, altered the microbial community structure, and thus evolved into a new intermediate niche favourable for FW biodegradation. The study highlights BSF gut microbiome as an engine for FW bioconversion, which is conducive to bioproducts regeneration from wastes.

## Introduction

An enormous amount of food wastes (FW) is produced annually worldwide [totalling about 1.6 gigatons] (De Clercq *et al*., 2017), which poses a great threat to the environment and has become an intensive public concern in many countries. Landfills and incineration are the major final destination of FW at present (Cerda *et al*., 2018); however, these methods could generate a large amount of leachate, odour, dioxin, and greenhouse gases (Yang *et al*., 2012, 2013; Liu *et al*., 2017). Given this situation, more effective and environmental‐friendly management of FW has become a top priority that is gaining increased public, academic, and political attention around the world.

Vermicomposting has gradually come to the front stage as a promising biotechnology for waste management owing to its technical feasibility, economic viability (Lim *et al*., 2016), and ability to transform wastes into stable biofertilizer (i.e. vermicompost) for soil amendments (Chen *et al*., 2015a; Wei *et al*., 2017). Moreover, vermicomposting can apparently reduce greenhouse gases that might occur in landfills (Adhikari *et al*., 2009; Schott *et al*., 2016; Nigussie *et al*., 2017), and even process organic leachates (Popa and Green, 2012 ). Among the various types of vermicomposting, utilizing the black soldier fly (BSF) larvae (*Hermetia illucens*) as a bioreactor has been commonly adopted to treat a variety of organic materials, including animal manure, FW, distillers’ grains, and municipal slurry (Popa and Green, 2012; Cickova *et al*., 2015; Wang and Shelomi, 2017). The harvested larvae biomass is commonly regarded as a value‐added feed for livestock(Cickova *et al*., 2015). Interestingly, BSF can produce different antimicrobial peptides that exhibit diverse inhibitory effects on various pathogens (Boccazzi *et al*., 2017; Vogel *et al*., 2018). Previous studies have concluded that BSF vermicomposting (BC) has strong adaptability and is a robust option for value‐added bioconversion of organic matter.

Research on vermicomposting to date has generally focused on the effectiveness of treatment, properties and function of products, and change of microbial community, mainly for earthworms (Gomez‐Brandon and Dominguez, 2014; Alavi *et al*., 2017; Villar *et al*., 2017). Few studies have identified the biological reasons for these outcomes when using BSF. Organic matter composition was closely associated with the relative abundances of the microbial metabolic pathways(Li *et al*., 2018). Vermicomposting involves a complex ecosystem of waste biodegradation and/or bioconversion by the joint action of worms/larvae and microorganisms (Zhang *et al*., 2012). This bioprocess also promotes organic degradation mainly by modifying the physical and biochemical status of compost, including the fragmentation, turnover, aeration of worms/larvae, and enzymatic hydrolysis (Yadav and Garg, 2011). However, knowledge on the biological interrelationship between the gut microbiota of BSF larvae and the microbial community over the course of BC is extremely limited. Gut microbiota of insects have different effects on the host, including the provision of necessary nutrients, stimulation of the immune system, removal of pathogenic microorganisms, sex determination and hormonal signalling and behaviours (Wada‐Katsumata *et al*., 2015; Zheng *et al*., 2017). Moreover, the insect gut plays a critical role in degradation of organic material (Engel and Moran, 2013) and various refractory matter, such as lignin (Li *et al*., 2017a), polysaccharides (Yang *et al*., 2018), and resistance genes (Wang *et al*., 2017a). Gut microbiota can be considered an independent ‘organ’ with its own metabolism capable of degrading wastes and generating products that benefit the host (Ricigliano *et al*., 2017). Interestingly, the features of the bacterial community in the larvae gut can be modified by changing its diet (Boccazzi *et al*., 2017; Vogel *et al*., 2018). A prominent example is the gut microbiota of caterpillars that are fully dependent on their diet (Hammer *et al*., 2017). Meanwhile, symbiosis with hosts can change environmental microbial communities (Wong *et al*., 2015; Hammer *et al*., 2017). Obviously, the gut flora of many insects is an open system, where external microbes can colonize and interact with the internal gut microbiome.

In this study, it is hypothesized that the gut microbiome of BSF larvae can influence the microbial community in FW composting and promote degradation of organic material. To test this hypothesis, the sources of bacteria in the larvae‐treated samples and larvae guts were determined, and the differences in metabolic functional groups and functional genes under natural composting (NC; i.e. natural pilled‐up storage) and BC were examined, with the microbial community structure and function assessed using 16S ribsosomal RNA (rRNA) amplicon sequencing and Phylogenetic Investigation of Communities based on Reconstruction of Unobserved States (PICRUSt). In addition, co‐occurrence network analysis was used to unravel the potential interaction between the BSF gut and BC bacteria. The results are expected to provide useful information for advancing the waste‐to‐resource recycling economy.

## Results

### The physical and chemical indices after composting

**Figure 1:**
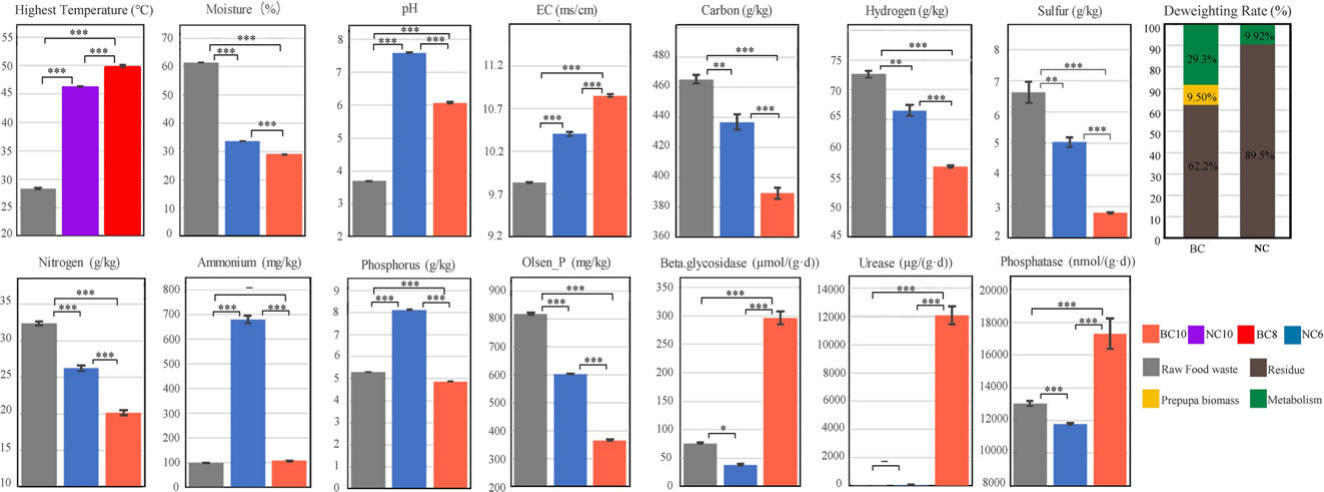
Physicochemical parameters, enzymatic activities and the weight of compost in food waste before and after black soldier fly larvae biodegradation. Natural composting (i.e. NC without BSF), BSF vermicomposting (i.g., BC). The Arabic number after NC and BC represents the days of processing. Residue represents the percentage of dry matter weight after NC (BC) in the total dry weight of added raw food waste. Metabolism represents the percentage of the weight metabolized by microorganisms in the total dry weight of added raw food waste. Error bars represent standard error of triplicate. ^–^ *p *>* *0.05; *p <* *0.05; **p <* *0.01; ***p <* *0.001.

The physicochemical parameters before and after BC and NC are presented in Fig. 1, and the associated dynamic features over time are shown in Fig. S1. During processing, the highest temperature in BC was 50.1°C, which was significantly higher (*P *<* *0.001) than that in NC. The moisture content in the raw FW prior to composting was significantly reduced (*P *<* *0.001) to 28.9% in BC10 samples, which was significantly lower (*P *<* *0.001) than that in the NC10 samples. The pH and conductivity rose from 3.68 and 9.83 ms cm^−1^ in the raw FW to 7.60 and 10.4 ms cm^−1^ in NC10 and 6.08 and 10.9 ms cm^−1^ in BC10, respectively. During BC, the total carbon, nitrogen, sulphur, and hydrogen content declined quickly (*P *<* *0.001) from 465, 32.4, 6.63, and 72.7 g kg^−1^ to 389, 20.2, 2.80, and 57.0 g kg^−1^ on day 10, respectively, and these values were significantly higher (*P *<* *0.001) than those in NC10. The total phosphorus content increased after NC, which was markedly higher (*P *<* *0.001) than in the BC10 and raw FW samples. β‐glucosidase (β‐GC), acid phosphatase (ACP), and urease activities of BC samples were significantly higher (*P *<* *0.001) than in the NC samples. On day 10, 9.50% of dry matter in the raw FW was transformed into prepupal biomass in the BC samples, and 29.3% of the dry matter was metabolized, which was obviously higher than in the NC samples.

### Change of bacterial community

**Figure 2:**
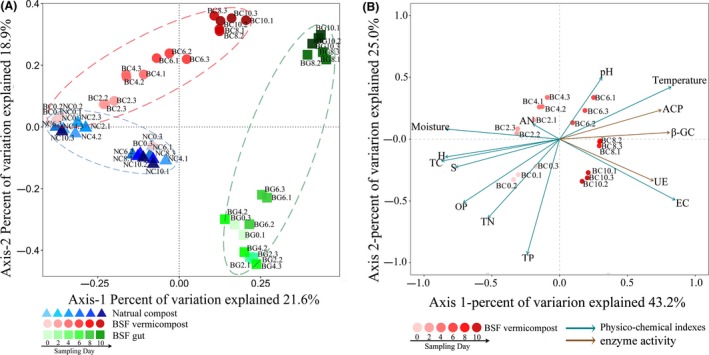
A. PCoA plots based on weighted UniFrac distances of samples collected from natural composting (i.e. NC without larvae inoculation), BSF vermicomposting (i.g., BC) and BSF gut samples (i.e. BG). The colour depth indicates the sampling days. Points of the same depth and colour represent three parallel samples taken on the same sampling day. B. Redundancy analysis (RDA) of environmental variables in BC. Abbreviations are shown as: ACP (Acid phosphatase), β‐GC (β‐glucosidase), UE (Urease), EC (Electric conductivity), TP (Total phosphorus), TN (Total nitrogen), OP (Olsen phosphorus), TC (Total carbon), AN (Ammonia nitrogen).

**Figure 3:**
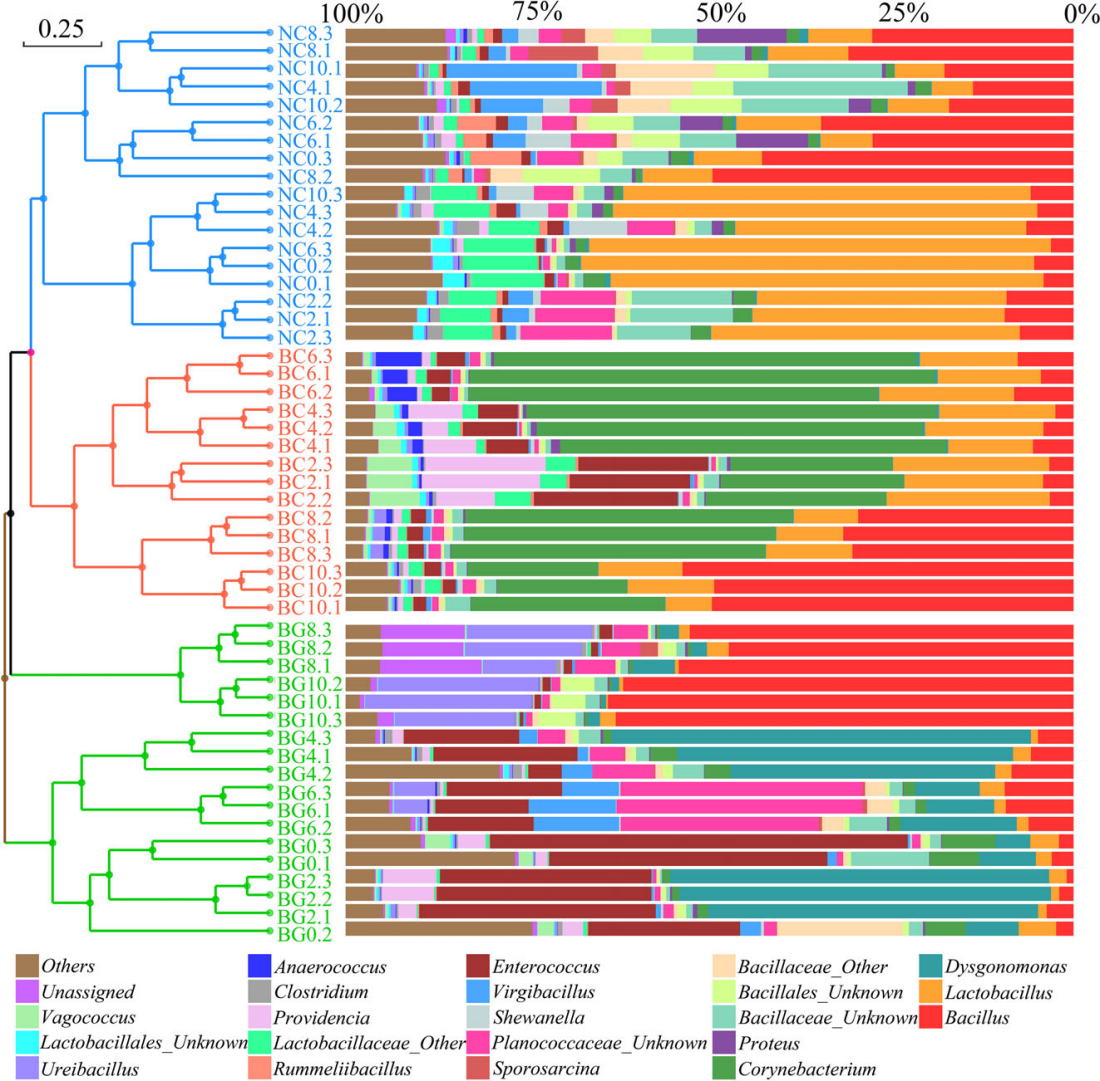
Relative abundance (%) of taxa at the genus level, clustered using UPGMA on the weighted UniFrac distances (Bray‐Curtis). Taxa with < 1% of reads were combined together as ‘Others’; while ‘Unassigned’ represents unclassified taxa at the genus level (Table S2).

The richness and diversity of bacterial species were significantly decreased in BC compared to NC (Fig. S2), and the latter had the lowest variance in the community structure (beta diversity). However, the BC samples were highly divergent and demonstrated distinct clusters, those of which formed during the first few days of BC were the closest in similarity to those in the raw FW, while those at the end of the process were more similar to the BG (BSF Gut)‐associated microbiota (Figs 2a and 3). The structure of the BG microbiome was also greatly changed. During the composting process, BC became significantly enriched with *Actinobacteria* but low in *Firmicutes* (*P *<* *0.05) compared to NC (Fig. S3A). The relative abundance of *Bacteroidetes* (20.4% of all sequences) in BG was significantly higher than in the BC and NC samples, which were mostly comprised of the *Porphyromonadaceae* family and *Dysgonomonas* genus bacteria (Fig. 3, Table S2) acting as the BG biomarkers (Fig. S4). However, *Corynebacteriaceae* (40.4%), rather than *Porphyromonadaceae*, was found dominant in the BC samples, which was classified as a BC biomarker and comprised mostly of *Corynebacterium,* 24 times more abundant than in the raw FW. Compared to the BC and BG samples, the *Lactobacillaceae* family bacteria were dominant in NC. The *Bacillaceae* family bacteria were dominant in all samples and largely comprised of genus *Bacillus* after processing (> 50%; Table S2).

**Figure 4:**
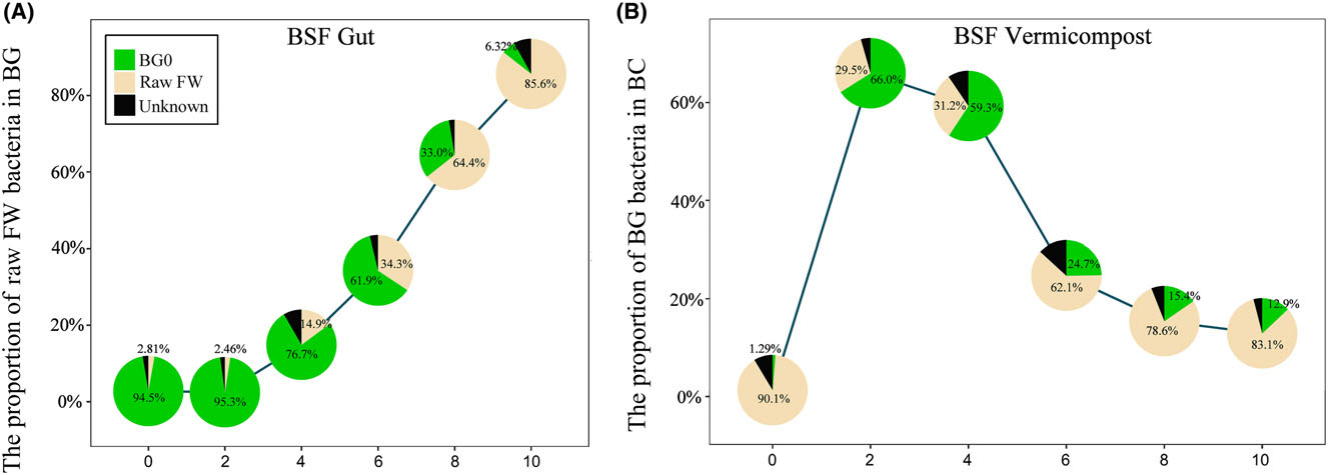
Source Tracking of microbial communities for BSF gut (A, i.e. BG) and BSF vermicompost (B, i.e. BC). ‘Unknown’ are taxa that could not be significantly sourced to either raw food waste (FW) or larvae gut. *X*‐axis indicates the day of sampling.

SourceTracker was applied to characterize the origin of taxa in the BC0‐BC10 and BG0‐BG10 samples (Fig. 4). The bacteria from the raw FW increased as a source to the BG microbiota, and gradually became a dominant contributor (85.6%) on day 10 (Fig. 4A). The bacteria in BC2 (66.0%) and BC4 (59.3%) were mainly derived from BG, while 24.7% of bacteria were derived from BG in BC6 and 12.9% occurred in BC10 (Fig. 4B). Figure S5 indicates that 401 kinds of OTUs were increased and 223 were decreased after 10 days of NC, while 263 were decreased and 155 were increased at the end of vermicomposting. The NC10 samples shared 399 kinds of OTUs (91.5% of all OTUs) with the raw FW samples, and the BC10 samples shared 189 kinds of OTUs (94.7% of all OTUs) with the raw FW and BG0 samples (Table S3).

### Metabolic function groups and functional genes

**Figure 5:**
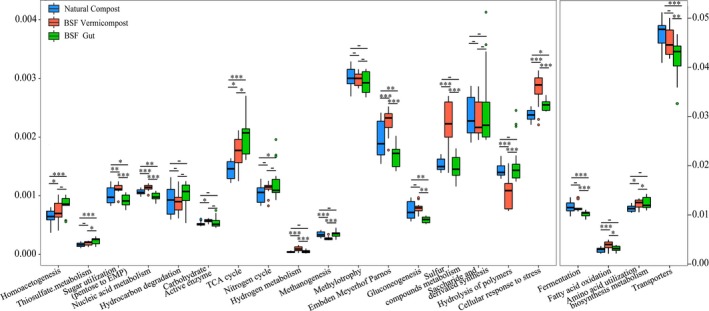
Box‐plots of relative abundance of functional groups based on FOAM database. ^–^ *p *>* *0.05; *p <* *0.05; **p <* *0.01; ***p <* *0.001.

**Figure 6:**
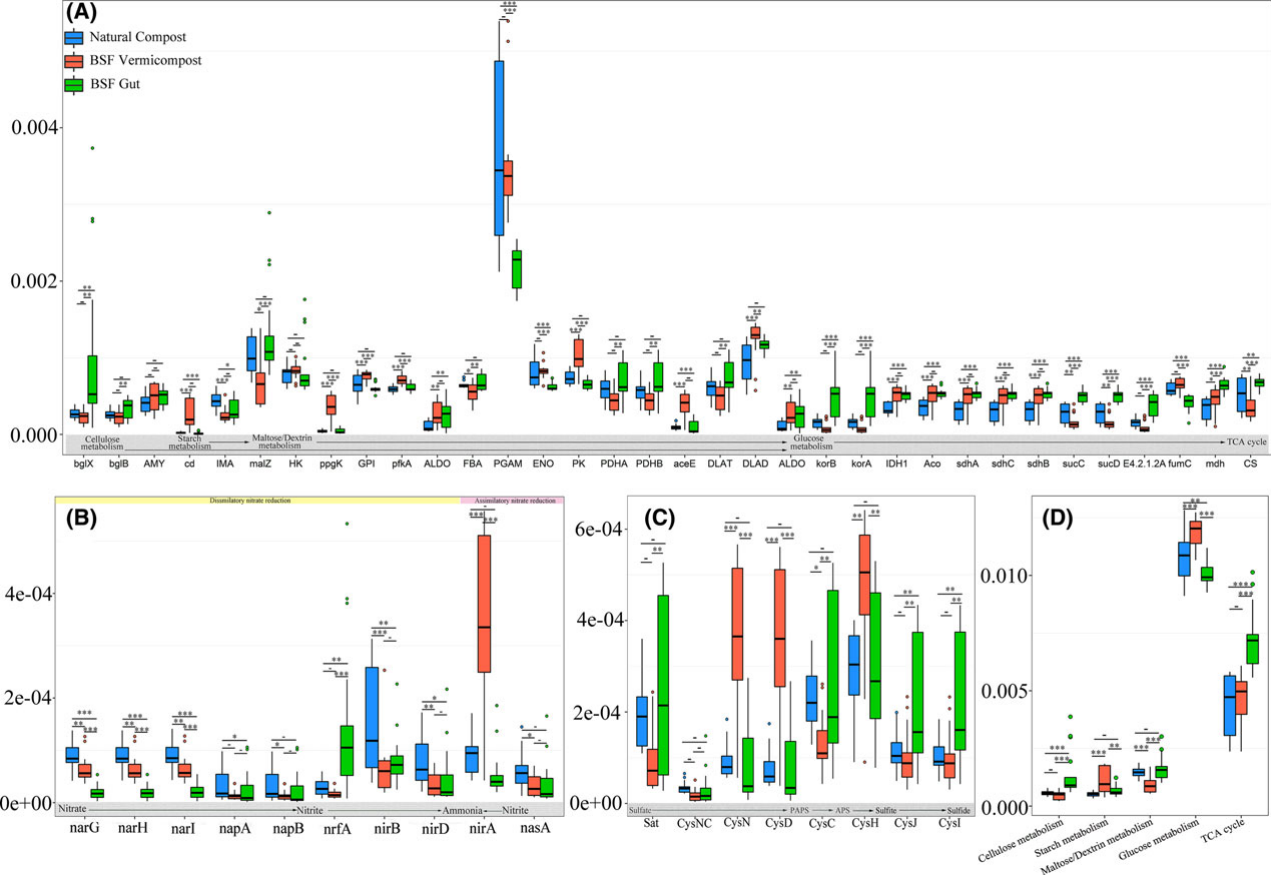
Box‐plots of relative genes abundance based on KEGG database. A. Functional genes related to carbon metabolism; (B) Functional genes related to nitrogen metabolism; (C) Functional genes related to sulphur metabolism; (D) Genes related to different steps of carbon metabolism. The circles above or below the box‐plots indicate Outlier. ^–^ *p *>* *0.05; *p <* *0.05; **p <* *0.01; ***p <* *0.001.

Eleven metabolic function groups were significantly strengthened (*P *<* *0.05) in the BC samples compared to those in NC, including carbohydrate‐active enzymes, tricarboxylic acid cycle, hydrogen metabolism, Embden‐Meyerhof‐Parnos, sulphur compound metabolism, homoacetogenesis and utilization of sugar, and ten were significantly higher than in the BG samples (Fig. 5). However, methanogenesis and hydrolysis of polymers under BC were significantly weakened (*P *<* *0.05) as opposed to those in NC. To better reveal the differences in metabolic functions of chemical elements, functional genes were identified associated with carbon, nitrogen, and sulphur metabolisms, as well as three kinds of enzymes, based on the KEGG database, and the most abundant genes were then retained (Fig. 6 and S6). Three genes (*CysNDH*) involved in sulphur metabolism in the BC samples were significantly higher than in NC and BG (Fig. 6C), while only one (*cysC*) was significantly lower than in NC. Fifteen of the 34 genes related to carbon metabolism showed higher abundance in BC compared to those in NC, and only three were significantly attenuated (Fig. 6A). Genes that functioned in the conversion of nitrate to nitrite (*narGHI*,* napAB*) were significantly lower in BC versus in NC (Fig. 6B). However, *nrfA* and *nirA* were the highest in BG and BC, respectively, compared to those in NC. The abundance of genes related to β‐GC (*bglB, E3.2.1.21*) was highest in BG, while genes related to urease (*ureABC*) were more abundant in BC than in BG and NC. Combining the BC and BG data showed that these selected genes and the associated metabolic functions were higher than in NC, except for *narGHI* and *nirB* (Table S4 and S5, Fig. S6 and S7).

**Figure 7:**
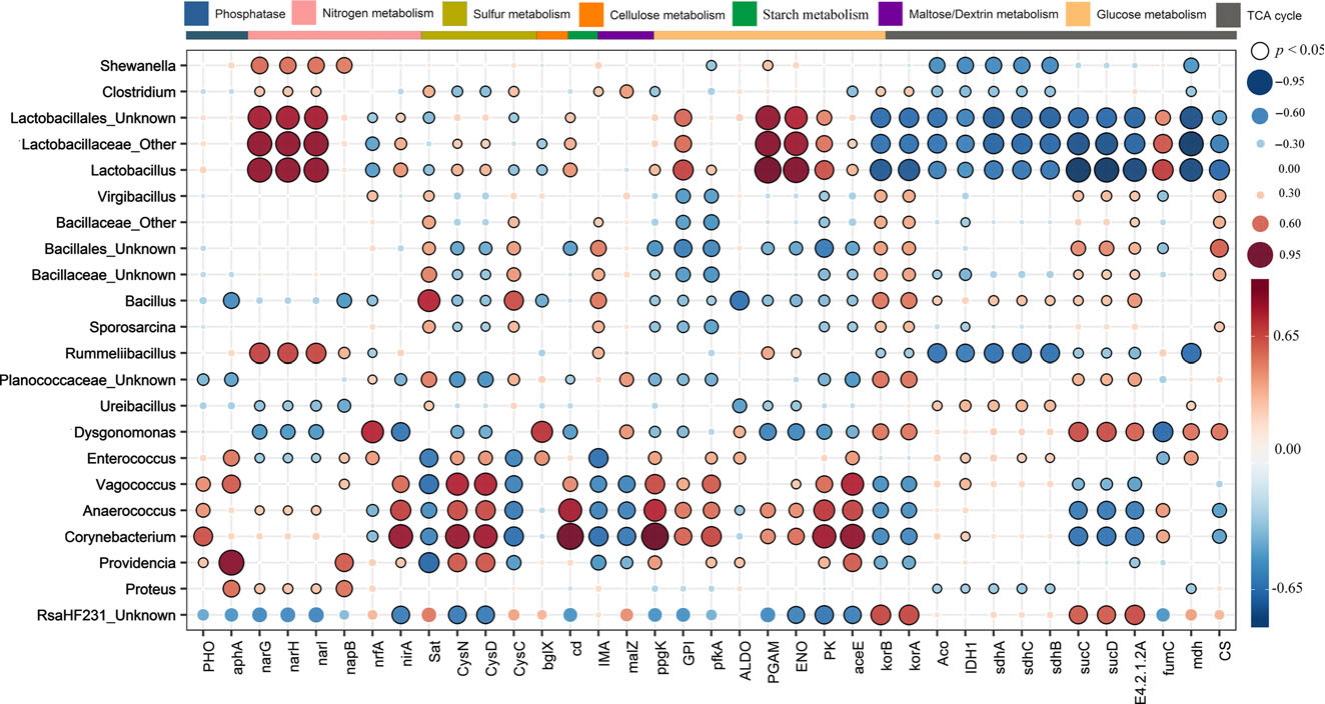
Spearman rank correlation between functional genes (rows) and genus (columns) across all groups. Blue and red colours represent positive and negative correlations, respectively. Circle size and colour saturation are proportional to the magnitude of the correlation. Statistically significant correlations (*p *<* *0.05) are indicated by black perimeters. Statistically significant correlations (*p *<* *0.05) are indicated by black perimeters. The different colour text on the *X*‐axis indicates number of genes in three groups higher than that of the other two groups (NC: Blue, BC: Orangered, BG: Green). Orangered or blue stars indicate the relative abundance of bacteria in NC significantly (*p *<* *0.05) higher or lower than BC.

To assess the potential relationships between the most abundant bacterial genus and the metabolic function genes, the Spearman rank correlations were calculated (Fig. 7), and the genes that were significantly related (*P *<* *0.05) to genus were retained. *Corynebacterium*,* Enterococcus*,* Vagococcus*,* Anaerococcus* and *Providencia*, whose relative abundances were higher in BC than in NC, correlated positively with *PHO*,* aphA*,* nirA*,* CysND*,* cd*,* ppgK*,* GPI*,* pfkA*,* ALDO*,* ENO*,* PK*,* aceE* and *fumC*. These genes were more abundant in BC than in NC and BG. The higher relative abundance of genes in BG, including *Sat*,* CysC*,* malZ*,* korAB* and *sucCD*, correlated positively with *Dysgonomonas*,* Ureibacillus*,* Enterococcus* and *RsaHF231*, which were also more abundant in BG than in BC and NC. *Dysgonomonas* was also positively correlated with *nrfA*,* bglX*,* mdh* and *CS*, which were enriched in BG as opposed to that in NC and BC. *Lactobacillaceae* that were more abundant in NC were positively correlated with *narGHI*,* GPI*,* PGAM*,* ENO*,* PK* and *fumC*, which were enriched in NC relative to BC. The bacteria belonging to *Bacillaceae* were positively correlated with *Sat*,* cysC*,* IMA*,* korAB*,* sucCD*,* E4.2.1.2A* and *CS*, while *Bacillus* was the predominant bacterium in all samples and had the highest abundance in BG samples.

Redundancy analysis was conducted to further identify the major environmental variables determining the microbial community structures (Fig. 2B). Axes‐1 and ‐2 explained 43.2% and 25.0% of the total variance observed in the analysis, respectively. Except for ammonia nitrogen (*r* = 0.15, *P *=* *0.11), the tested variables were significantly associated (*P *<* *0.05) with the BC bacterial communities (Table S6), especially sulphur (*r* = 0.84, *P *=* *0.001), carbon (*r* = 0.80, *P *=* *0.001), β‐GC (*r* = 0.80, *P *=* *0.001), and moisture content (*r* = 0.81, *P *=* *0.001).

### Co‐occurrence networks

**Figure 8:**
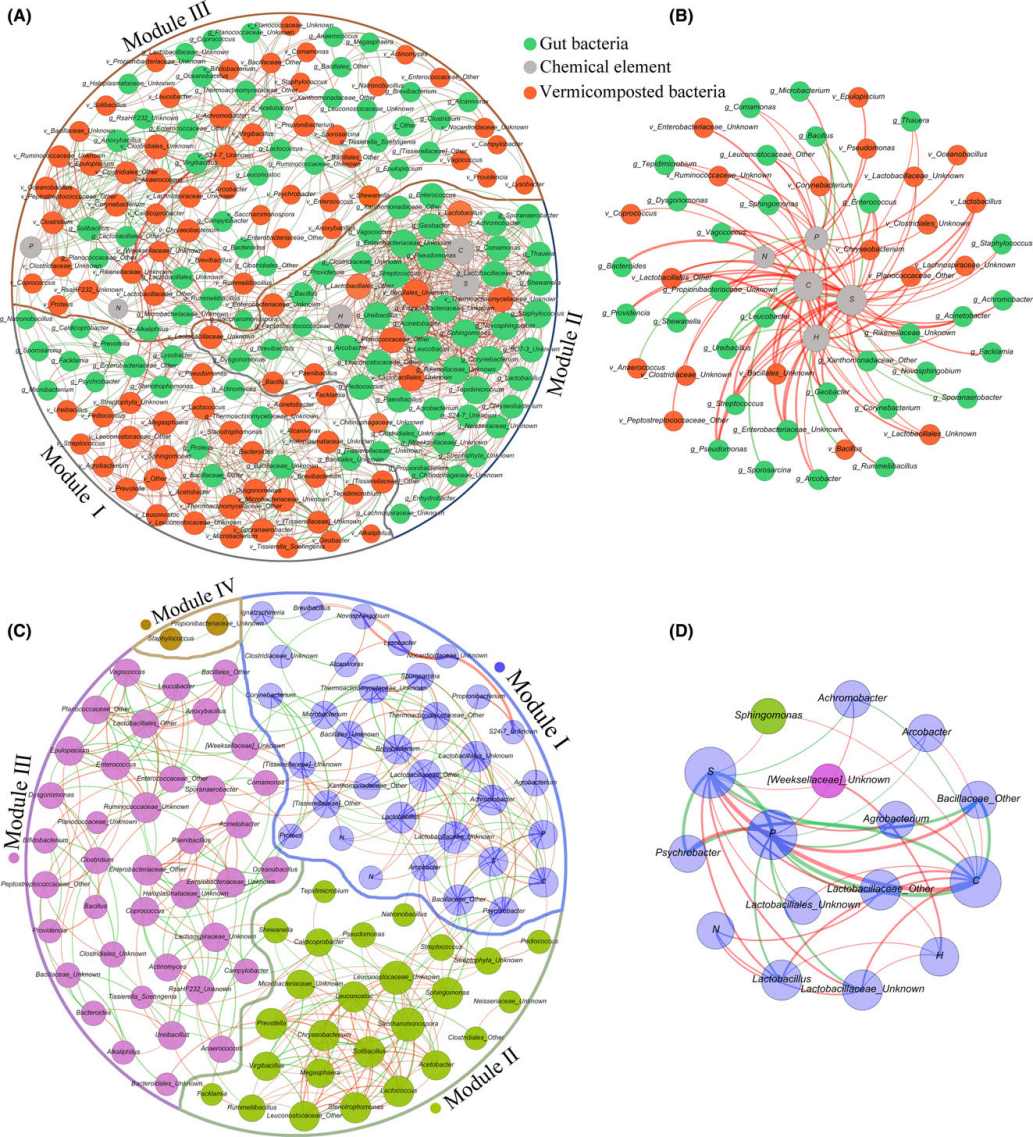
A. Network of co‐occurring bacterial genera and elements based on local similarity analyses in vermicomposting. The average degree of this network is 13.04; average path length is 3.62; network density is 0.066; and the clustering coefficient is 0.55. B. Co‐occurrence network visualize the environment‐microbe interactions among BC, BG and chemical elements. The name beginning with ‘v_’ and ‘g_’ indicate bacteria belong to BC and BG, respectively. C. Network of co‐occurring bacterial genera and elements based on local similarity analyses across all samples in NC. The average degree of this network is 8.20; average path length is 3.45; network density is 0.082; and the clustering coefficient is 0.50. D. Co‐occurrence network visualize the environment‐microbe interactions in NC. The same node colours represent nodes belonging to the same modules. The thickness of each edge is proportional to the value of the local similarity score. Green and red edges represent positive and negative correlations, respectively. Abbreviations are shown as: S (Sulphur), P (Phosphorus), N (Nitrogen), H (Hydrogen), and C (Carbon).

The co‐occurrence network was constructed to elucidate biotic interactions among BG microbiota, BC bacteria, and chemical elements (Fig. 8A). The network contains 200 nodes with 1304 edges. The modularity index in the present study was 0.57, indicating a valid modular structure in the network (Newman, 2006). The nodes were divided into three modules (I‐III) consisting of 38, 60 and 102 nodes (genera), respectively, with three different borders. The size of each node is proportional to the number of connections (degree). Bigger nodes indicate stronger and more significant correlations with other nodes and, therefore, may be important members in the community (Sun *et al*., 2017). The most densely connected node in each module is defined as a ‘hub’. If there were multiple nodes with the same degree in the same module, the nodes with higher betweenness centrality were chosen as hubs (Lupatini *et al*., 2014). Based on these criteria, *Thermoactinomycetaceae* and *Dysgonomonas* were identified as hubs for module I; *Anaerococcus* was identified as a hub for module III; and *Corynebacterium*,* Sphingomonas*, and *Staphylococcus* were identified as hubs for module II (Table S7). Most of the nodes in module I belonged to the BC taxa, which were part of the BG bacteria and had high degrees. The nodes in module II were mostly in the BG taxa, some of which belonged to the BC bacteria. Carbon, hydrogen, and sulphur were found in module II, and all had degrees > 20, especially sulphur, which had the same degree as the hub in module II. Nitrogen and phosphorus were evident in module III, and the nodes connected to these elements mainly belonged to BC (Fig. 8B). In addition, these chemical elements were associated with 32 kinds of bacterial families. At the same time, the co‐occurrence network of NC contained 101 nodes with 414 edges and a modularity index of 0.56, indicating a valid modular structure (Fig. 8C). The nodes were further divided into four modules as indicated by four different colours and borders in Fig. 8. Modules I‐IV had 34, 26, 39 and 2 nodes, respectively. Hubs and nodes with higher degrees in module I belonged to *Lactobacillaceae*, and all chemical elements were in this module. *Ruminococcaceae*,* Saccharomonospora*, and *Staphylococcus* were hubs in modules II‐IV, respectively (Table S8). The nodes connected to chemical elements were mainly *Lactobacillaceae* (Fig. 8D).

## Discussion

In the present study, it was found that the content of total carbon, hydrogen, nitrogen, and phosphorus after 10 days of BC was significantly lower (*P *<* *0.001) than those in NC (Fig. 1). This chemical element trend was similar to that reported previously in studies using housefly (Wang *et al*., 2013) and earthworm (Villar *et al*., 2016). The per cent reduction in dry matter by BC (38.8%) was also similar to those in the previous reports (Eghball *et al*., 1997; Diener *et al*., 2009). Some research indicated that microbes and their secreted enzymes played a critical role in biological and biochemical degradation of organic matter during composting (Pages *et al*., 2007; Guo *et al*., 2012). In our study, β‐GC, ACP and urease activity were significantly higher in BC than in NC at all time points (Fig. S1), which was consistent with the findings from the early studies (Villar *et al*., 2016; Sudkolai and Nourbakhsh, 2017). Since enzyme activity is closely related to the properties of microorganisms (Tiquia *et al*., 2002), our results suggested that the presence of BSF larvae increased FW degradation and could be a consequence of the differences in the microbial communities.

Previous studies have shown that earthworm guts and vermicompost generally have greater bacterial diversity than naturally piled up earthworms (Bernard *et al*., 2012; Chen *et al*., 2015b). In contrast, the BG and BC materials in the current study possessed lower bacterial species richness compared with NC (Fig. S2). This difference may be due to the physicochemical properties of the insect larvae gut that exerted strong pressure on bacteria from external environment, such as low pH, secreted antimicrobial substances and diverse proteolytic enzymes (Park *et al*., 2014; Wang *et al*., 2017a). Moreover, the microbial community of BG and BC samples changed greatly over time, becoming more and more similar (Figs 2A and 3), and the SourceTracker data indicated that some of the bacteria identified in BC came from BG. Similar studies have verified the dynamic community changes in a housefly larvae bioreactor fed with swine manure (Wang *et al*., 2017a). These findings suggest that BG can reshape the microbial community of vermicompost. In addition, as the composting time increased, more and more bacteria found in the raw FW colonized BG (Fig. 4). Interestingly, a previous study showed that the gut of caterpillars lacked any resident microbiome and was fully colonized (or invaded) by its food microbiome (Hammer *et al*., 2017). The BG was also significantly affected by exogenous bacteria, similar to the caterpillar gut reported above. This may explain why studies feeding BSF with different diets showed changes in the structure of gut bacteria (Jeon *et al*., 2011; Boccazzi *et al*., 2017). Besides, a larger part of OTUs in the NC10 samples came from the environment compared to those in the BC10 samples (Fig. S5, Table S3), indicating that BC was more robust than NC. Consequently, most of the metabolic function groups found in BC were higher than in NC, while the abundance of most genes related to carbon metabolism, enzymes, and all genes related to sulphur metabolism in BC was higher than in NC (Fig. 6 and S6). In the BC samples, the relative abundance of hydrolysis of polymers and methanogenesis was significantly lower than in NC, which might be because the naturally piled compost (NC) formed small anaerobic pockets (Fig. 5). Hence, our current results suggest that the metabolic functions in FW biodegradation were strengthened by BSF larvae relative to NC.

It is well‐known that the metabolic functions of bioprocesses are closely related to the features of microbes (Cusack *et al*., 2011; Knelman *et al*., 2012; Lopez‐Gonzalez *et al*., 2015; Li *et al*., 2017b; Wang *et al*., 2017b). It was found in this study that the functional genes and microbial taxa possessed significantly similar trends among all groups (Fig. 7). For example, *Corynebacterium*,* Enterococcus*,* Vagococcus*,* Anaerococcus* and *Providencia*, which were significantly enriched in BC samples versus in raw FW and NC (Fig. 3), had significantly positive correlations with the associated genes and metabolic function groups that had higher abundance in BC, such as carbohydrate‐active enzymes, hydrogen metabolism, nitrogen cycle, and sulphur compound metabolism (Fig. S8). *Corynebacterium‐*related bacteria have been found growing on a variety of sugars, organic acids, and alcohols as single or combined carbon and energy sources, acting as a workhorse for large‐scale production of amino acids (Eikmanns and Blombach, 2014). It was found in the present study that *Lactobacillales*‐related bacteria with higher abundance in raw FW and NC were significantly attenuated by BC (but still maintaining a high abundance), and positively correlated with the genes in fermentation that had high abundance in NC (Fig. S8). *Lactobacillales*‐related bacteria have been widely used in the fermentation industry due to the advantages in carbohydrate, peptide and lipid metabolism (Abdel‐Rahman *et al*., 2011 ). These results indicated that the significant change of bacteria after BG transformation caused an increase in the abundance of various metabolic genes in BC.

Co‐occurrence networks, whose nodes represent taxa and edges and reflect significant co‐occurrences between taxa (Barberan *et al*., 2012), are a powerful tool for exploring taxon coexistence in complex microbial communities (Eiler *et al*., 2012; Williams *et al*., 2014). A previous reconstructed network showed that the gut microbiome of housefly larvae altered the bacteria in vermicompost and depressed antibiotic ‘resistome’ hosts successfully (Wang *et al*., 2017a). Modularity has also been suggested to be an important attribute of community resilience and ecosystem stability (Olesen *et al*., 2007). While modules found in a co‐occurrence network can potentially indicate direct or indirect interactions among microbes, these interactions may also be due to the shared niches or a high level of phylogenetic relatedness (Faust and Raes, 2012). It is worth noting that many bacterial taxa with low abundance could also play disproportionately large roles in niches and functions (Van Goethem *et al*., 2017; Sun *et al*., 2018). Based on these theories, a network graph was constructed composed of three modules (Fig. 8A). Modules I and II were mainly composed of BG and BC bacteria, respectively, whose taxa might likely specialize in BC and BG (restricted to certain habitats). On the other hand, the two types of bacteria in module III may be regarded as generalists (broadly distributed across BC and BG). However, some BC bacteria are found in module II, which had high degrees (> 20) and connected nodes that mainly belonged to the BG bacteria. The same situation was also found in module I, and the relative abundance of these bacteria was low. Interestingly, the most dominant bacteria in BC (*Corynebacterium*) and BG [*Dysgonomonas*] (Fig. S4) knitted the hubs in modules II and I, respectively, and the relationship between these two bacteria might be regulated by the bacteria of less abundance (*Brevibacillus*; Fig. S9). The relative abundance of the other hubs in modules I and II was also low, such as *Thermoactinomycetaceae* in BC and *Sphingomonas* in BG. These results indicated that the bacteria in BC and BG can greatly influence each other, and were related to and regulated by low abundance taxa. Symbiotic microbes of insects could be released from their host, thus facilitating insect invasion (Traveset and Richardson, 2014). Those symbiotic interactions could trigger pronounced changes in ecosystems, including population decline, reduced biodiversity, alterations of structure and function of ecosystems (Traveset and Richardson, 2014), and even differentiation (Jackson and Britton, 2014). Symbiotic microbes can be considered invaders that adapt themselves to a new environment easier than the host insects due to their relatively shorter life cycles (Lu *et al*., 2016). In our study, chemical elements coexisted in module II, with all having very high degrees, meaning that they played crucial roles in the BG environment. Sulphur had the same degree as the hub in module II, suggesting that it is an indicator of co‐occurring taxa in this module. Furthermore, the redundancy analysis (Fig. 2B) demonstrated that carbon, nitrogen, and sulphur significantly influenced community structure in BC. There was a negative correlation between the microbial community structure of samples and the nutrient elements (C, P, H, etc.) over time. Also, as shown in Fig. 8B, there were major negative correlations between nutrient elements and bacteria with high abundance in BC, such as *Corynebacterium, Bacillus, Lactobacillus*. These results showed that with the decrease of nutrient elements in compost, the microbial community structure of BC changed greatly. As such, the reduction of nutrients were due to the acquisition of BSF as raw materials for growth and metabolism. These results suggest that BG might influence bacterial communities in BC by acquisition of chemical elements. Thus, the BG bacteria likely occupied the niche of BC bacteria (i.e. ecological invasion).

Initially, the BG0 bacteria and raw FW (BC0) with large weighted distances were considered as two independent niches (Figs 2A and 3). Accumulating evidence suggests that one of the main factors determining whether microbes have successfully invaded is whether they can occupy certain environments to impact microbial communities in native environments as a way of facilitating greater microbial invasion (Si *et al*., 2013; Xiao *et al*., 2014). In the present study, when raw FW was inoculated with BSF larvae, another new niche was formed via vermicomposting. Many of the bacteria with high abundance in BC were located in module III (Fig. 8A), including *Corynebacterium*,* Vagococcus*,* Enterococcus*,* Anaerococcus*, and *Providencia*. These bacteria had significant positive correlations with many functional genes (Fig. 7). Furthermore, some BC and BG bacteria also coexisted in each other's niches. These results suggested that the BG and BC bacteria were both successful invaders and could form an intermediate habitat between BG and BC. This coexistence, in turn, enhanced the intensity of organic matter degradation. Also, the correlations between the BG and BC bacteria were more negative (> 50%; Table S9), indicating competitive correlations that resulted in the decline of α‐diversity in the matrix during the vermicompost process. In addition, since communities with high species diversity were more stable with more functions (Schnitzer *et al*., 2011), the network of BC and subnetwork of chemical elements in this study were found to be more complicated than those of NC (Fig. 8), which explained why BC was more robust, faster, and had more diverse material degradation capabilities.

Our results suggest that the BG microbiome could invade and interact with the BC bacteria, altered the microbial community structure of BC, and built an intermediate habitat between the gut and vermicompost. This intermediate habitat might be able to promote reproduction of bacteria beneficial to metabolism, thereby improving FW biodegradation. These results are extremely useful for bioproducts regeneration from wastes for recycling economy development. This unique phenomenon have revealed the biological basis of the strong metabolic function of BC and provided perspectives for future studies on investigating interactions between the gut microbiomes and the external environment based on network and invasion hypotheses. Many studies have confirmed that some insect gut bacteria possess the ability to degrade refractory substances (Li *et al*., 2017a; Yang *et al*., 2018), and many bacteria can also enhance or alter their metabolic functions through genetic engineering (Vitorino and Bessa, 2017; Panda *et al*., 2018). Considering that approximately half of the raw FW dry matter remained after 10 days of BC in our study, the colonization attempts by other insect‐oriented or commercialized bacterial strains were worth further investigation. Furthermore, since fungal communities are also known to have a great impact on invasion and the environment (Lu *et al*., 2016), further work is also needed to determine the effects of insect gut fungi on the metabolic function and microbial communities of vermicompost.

## Experimental procedures

### Experimentation and sampling

The life cycle of BSF can be divided into four stages: egg, larva, pupa, and adult fly. During the later period of larval stage (approximately 10–15 days), the prepupae migrates to the dry and dark site and converts into pupa. Before converting, the prepupae can be harvested, which is commonly regarded as a value‐added feed for livestock (Cickova *et al*., 2015). A full‐scale farm using BSF larvae (*Hermetia illucens* L.) for FW biodegradation (BSF farm) was established by the Hangzhou GuSheng Biotechnology Co. Ltd (30°40′54.81″N, 120°17′31.23″E), HangZhou, ZheJiang Province, China. The raw FW was collected from the nearby villages, and was processed into slurry using a pulper to pass through a sieve with 3.5‐mm openings. To maintain a moisture content of around 70%, the FW slurry was mixed with rice husk powder. The FW samples obtained after the above steps were frozen at −20°C and thawed before use.

A continuous feeding strategy based on the farming experience of GuSheng accompany was applied to treat FW (Fig. S10). The BSF larvae of 5 days old (approximately 8000 larvae) obtained from BSF breeding room in GuSheng farm, were added to plastic containers (40 × 70 × 15 cm for each, fed with the prepared FW) as the BC bioreactor. The amount of inoculative larvae and FW were determined according to previous research (Diener *et al*., 2009 ) and the operational experience of GuSheng farm. BC samples were collected every other day for 10 days (BC0, BC2, BC4, BC6, BC8, and BC10) using the longitudinal time‐course sampling protocols (Fig. S11); BC0 represents the initial FW before larvae inoculation. Larvae were collected from the BC bioreactor every 2 days to analyse their gut microbiome [BSF larvae gut samples (BG): BG0, BG2, BG4, BG6, BG8, and BG10]. At the same time, samples from the natural composting without larvae were also collected every 2 days as a control (NC: NC0, NC2, NC4, NC6, NC8, and NC10). Each treatment was replicated three times. One sample was taken from each treatment at day of sampling for chemical analysis and DNA extraction, and all tests were done in triplicate. Since BC0 and NC0 samples are the same, only 51 samples had been tested in total, and six samples were obtained from each container. Fresh samples were either processed immediately or stored at −80°C prior to downstream analysis.

### Chemical analyses

Temperatures in the compost sites were measured by a kerosene thermometer. The moisture content was determined from the loss in the sample weight after drying at 105°C for 48 h. Electric conductivity and pH were measured as the ratios of biomass weight to deionized water suspension (1:2.5 and 1:5) using a conductivity meter (DDSJ‐308A, Hangzhou, China) and a digital pH meter (Leici PHB‐4, Shanghai, China), respectively. Total carbon, nitrogen, hydrogen and sulphur content were determined using an elemental analyzer (Elementar, Frankfurt, Germany). In accordance with the Standardization Administration of the People's Republic of China (http://www.sac.gov.cn/), standard methods coded as NY 525‐2002, LY/T1229‐1999, and GB/T8573‐1999 were used to analyse the levels of total phosphorus, ammonium nitrogen, and bioavailable phosphorus (Olsen‐P); urease activity, β‐glucosidase (β‐GC) and acid phosphatase activity (ACP) were quantified by measuring the breakdown rate of urea, *p*‐nitrophenyl β‐D‐glucopyranoside and *p*‐nitrophenyl‐phosphate using appropriate assay kits (Solarbio, Beijing, China) following the manufacture's protocols.

### DNA extraction and 16S rRNA sequencing

DNA of all samples was extracted using a DNeasy Power Soil kit (MO BIO Laboratories, Carlsbad, CA, USA) following the manufacturer's protocol and normalized to equal concentrations before downstream processing. Before BG sample DNA extraction, the collected larvae were starved for 8 h to empty their ingested contents. To meet the minimum amount of DNA, approximately 60–600 larvae were rinsed with sterile water after alcohol cleaning, then anatomized to extract total DNA from the midgut and hindgut (Wang *et al*., 2017a).

The DNA samples were amplified and sequenced on a 250‐bp pair‐end Illumina HiSeq sequencer (Novogene, Beijing, China). The V4–V5 region of the 16S rRNA gene was amplified in triplicate using the F515/R907 primer set (Jing *et al*., 2015). Raw reads were deposited into the DNA databank of Japan sequence read archive database (Accession no. SRP116776). Raw sequence data were processed using the qiime v1.9.1 pipeline (Caporaso *et al*., 2010). First, the forward and reverse Illumina reads were joined using the default setting. Then, the multilane fastq data were demultiplexed and quality filtered (Q30 ≥ 75% and Q20 = 100%). Chimeras were identified using the function ‘identify_chimeric_seqs.py ‘with ‘‐m usearch61’ and then removed. A total of 3 682 485 reads were obtained with each sample having over 45 403 reads. Filtered sequences were clustered into operational taxonomic units (OTUs) using the function ‘pick_open_reference_otu.py’ against the Greengenes reference database [13_8 release] (McDonald *et al*., 2012) based on a 97% consensus threshold. Taxonomic classification of the representative sequence for each OTU was done using the Ribosomal Database Project's classifier taxonomy after removing singleton reads. Functional profiling was inferred from the 16S rRNA marker gene sequences using PICRUSt on the Galaxy web platform (Blankenberg *et al*., 2010). The identified Kyoto Encyclopaedia of Genes and Genomes (KEGG) orthologs was used to map the metabolic function groups based on the Functional Ontology Assignments for Metagenomes [FOAM] (Prestat *et al*., 2014) by HMP Unified Metabolic Analysis Network 2 (Abubucker *et al*., 2012).

The reads were rarefied or normalized to correct for the differences in sequencing depth if needed before further analysis. QIIME (http://qiime.org.html) was used to analyse the alpha and beta diversity of bacterial community structures (Caporaso *et al*., 2010). Sequences were rarefied to 39 000 for doing the diversity analysis. The potential sources of microbial communities in a set of input samples (FW and BC) from the larvae gut‐associated microbiota was profiled using SourceTracker [http://qiime.org/tutorial/source_tracking.html] (Knights *et al*., 2011). The BC and BG samples were considered daily ‘variables,’ while the raw FW and BG0 were considered major known ‘sources’. The reads that were not mapped to our input sources were marked ‘unknown’. Veen was used to represent the shared and exclusively detected OTUs among the five groups (raw FW, BC10, NC10, BG0, and BG10).

### Statistical analyses

Statistical differences were assessed using the least significant difference test in the r package Agricolae (v.1.2.8). Standard deviation (SD) was calculated by r package Rmisc (v.1.5). Principal coordinate analysis of the weighted UniFrac distances, redundancy analysis, and mantel test (permutations = 999) was calculated in the r package Vegan [v.2.4.4] (Dixon, 2003). Spearman rank correlations were then calculated between the relative abundances of functional groups/genes and bacteria in the r package Psych (v.1.7.8) to assess the extent to which the different bacteria were related to the functional community structure (|*r*| > 0.6, *P *<* *0.05). Additional r packages that were used for statistical analyses included ggplot2 (v.2.2.1), plyr (v.1.8.4), Reshape (v.1.4.2) and rcolorbrewer (v. 1.1.2).

The linear discriminant analysis, Effect Size [LEfSe] [http://huttenhower.sph.harvard.edu/galaxy] (Segata *et al*., 2011), was used with the factorial Kruskal–Wallis sum‐rank test (α = 0.05) to identify the taxa with significant differential abundances between categories using one‐against‐all comparisons, and to estimate the effect size of each different abundant feature (the logarithmic linear discriminant analysis score > 2.0). The significant taxa were used to generate a taxonomic histogram illustrating the differences between the samples. Additionally, the taxonomic levels were limited from domain to genus in cases of distraction due to redundant data.

The local similarity analysis (Xia *et al*., 2011) was performed to assess the correlations between the relative abundances of bacterial genera and chemical elements in BC and BG samples during the vermicomposting process. To filter data and reduce network complexity, the taxa were further filtered to have a relative abundance of no < 0.1% of the total. The rest of the taxa were placed into the ‘other’. The connections between two nodes indicating a strong (local similarity| > 0.6) and significant (*P *<* *0.05) correlation were reserved. The size of each node was proportional to the number of significantly positive/negative correlations (degree), and the thickness of each connection between the two nodes (edge) was proportional to the correlation coefficient (local similarity), ranging from |0.6| to |1|. The networks were visualized using Cytoscape [v.3.6.0] (Kohl *et al*., 2011) and Gephi [v.0.9.1] (Bastian *et al*., 2009).

## Conflict of interest

None declared.

## Supporting information


**Fig. S1.** The dynamic change of physico‐chemical parameters during BSF vermicomposting (BC, Orangered in color) and natural composting (NC, Blue in color).
**Fig. S2.** Alpha‐diversity measurement among studied samples.
**Fig. S3.** Relative abundance (%) of taxa at the level (A) phylum and family level (B), The samples are arranged according to the clustering tree.
**Fig. S4.** the dynamic change of relative abundance (%) of taxa at genus level in NC (A), BC (B) and BG (C), Taxa with < 1% of reads were combined together as ‘Others’; while ‘Unassigned’ represents unclassified taxa at the genus level.
**Fig. S5.** Exclusive and shared OTUs (non‐singleton OTUs, based on 97% reads similarity) among raw FW, larvae gut (BG0 and BG10), NC10 and BC10, with number representing OTUs found in each segment (Table S3).
**Fig. S6.** Box‐plots of relative genes abundance about three kinds of enzyme based on KEGG database.
**Fig. S7.** (A) Box‐plots of relative abundance of functional groups based on FOAM database.
**Fig. S8.** Spearman rank correlation between metabolic functional group (rows) and bacteria(columns) from Lefse across all groups.
**Fig. S9.** Subnetwork organized between the top most abundant bacteria and other genus.
**Fig. S10.** A continuous‐feeding vermicomposting practice is applied to treat food waste samples with the aid of black soldier fly (*Hermetia illucens L*) larvae.
**Table S1.** The dynamic of Physico‐chemical parameters, enzymatic activities and prepupa weight.
**Table S2.** Detected microorganism at phylum, family, order, genus and species level (only list item whose average relative abundance is more than 1%) in raw food waste, BSF vermicompost, and BSF gut as determined by 16S rRNA sequence analyses.
**Table S3.** Numbers of normalized reads for each shared and exclusively detected OTU within the indicated segments of the Venn diagram.
**Table S4.** The average relative abundance of metabolic pathway based on FOAM database. The data are expressed as mean percent (standard error).
**Table S5.** The relative abundance of genes involved in the metabolism of carbon, nitrogen and sulfur in different groups, based on KEGG database.
**Table S6.** Statistic using mantel test to present the influence of environmental variables in RDA.
**Table S7.** Important coefficients for co‐occurrence network of BC and BG.
**Table S8**. Important coefficients for co‐occurrence network of NC.
**Table S9.** The numbers and properties of the nodes and edges of the co‐occurrence networks.
