# Supplementary material for: Black soldier fly larvae (Hermetia illucens) strengthen the metabolic function of food waste biodegradation by gut microbiome
Source: Microb Biotechnol. 2019 Mar 18;12(3):528–43. doi: 10.1111/1751-7915.13393 (PMC6465238; doi:10.1111/1751-7915.13393)
Supplement: Supplementary file 1 — Fig. S1. The dynamic change of physico‐chemical parameters during BSF vermicomposting (BC, Orangered in color) and natural composting (NC, Blue in color). Fig. S2. Alpha‐diversity measurement among studied samples. Fig. S3. Relative abundance (%) of taxa at the level (A) phylum and family level (B), The samples are arranged according to the clustering tree. Fig. S4. the dynamic change of relative abundance (%) of taxa at genus level in NC (A), BC (B) and BG (C), Taxa with < 1% of reads were combined together as ‘Others’; while ‘Unassigned’ represents unclassified taxa at the genus level. Fig. S5. Exclusive and shared OTUs (non‐singleton OTUs, based on 97% reads similarity) among raw FW, larvae gut (BG0 and BG10), NC10 and BC10, with number representing OTUs found in each segment (Table S3). Fig. S6. Box‐plots of relative genes abundance about three kinds of enzyme based on KEGG database. Fig. S7. (A) Box‐plots of relative abundance of functional groups based on FOAM database. Fig. S8. Spearman rank correlation between metabolic functional group (rows) and bacteria(columns) from Lefse across all groups. Fig. S9. Subnetwork organized between the top most abundant bacteria and other genus. Fig. S10. A continuous‐feeding vermicomposting practice is applied to treat food waste samples with the aid of black soldier fly (Hermetia illucens L) larvae. Table S1. The dynamic of Physico‐chemical parameters, enzymatic activities and prepupa weight. Table S2. Detected microorganism at phylum, family, order, genus and species level (only list item whose average relative abundance is more than 1%) in raw food waste, BSF vermicompost, and BSF gut as determined by 16S rRNA sequence analyses. Table S3. Numbers of normalized reads for each shared and exclusively detected OTU within the indicated segments of the Venn diagram. Table S4. The average relative abundance of metabolic pathway based on FOAM database. The data are expressed as mean percent (standard error). Table S5. The relati [file MBT2-12-528-s001.docx]

Supporting Information

Black Soldier Fly Larvae (*Hermetia illucens*) Strengthen the Metabolic Function of Food Waste Biodegradation by Gut Microbiome

Cheng-Liang Jiang^a^, Wei-Zheng Jin^b^, Xin-Hua Tao^a^, Qian Zhang^b^, Jun Zhu^d^, Shi-Yun Feng^a^, Xin-Hua Xu^a^, Hong-Yi Li^a^, Ze-Hua Wang^c^, Zhi-Jian Zhang^a,^*

^a^ College of Environmental and Resource Sciences, ZheJiang University, HangZhou 310058, China

^b^ HangZhou GuSheng Biotechnology Co. Ltd, HangZhou 311108, China

^c^ College of Agriculture and Biotechnology, ZheJiang University, HangZhou 310058, China

^d^ Department of Biological and Agricultural Engineering, University of Arkansas, Fayetteville, AR 72701, USA.

^*^Corresponding author: ZhiJian Zhang

*E-mail addresses:* [zhangzhijian@zju.edu.cn](mailto:zhangzhijian@zju.edu.cn)

Phone: +86 571 8898 2057

Fax: +86 571 8898 1719

**Table S1** The dynamic of Physico-chemical parameters, enzymatic activities and prepupa weight. The weight of compost before and after black solider fly vermicomposting(BC) and natural composting(NC). The data are expressed as mean percent (standard deviation). n represents the number of repetitions.

|  | Treatment/Day | 0(n=3) | 2(n=3) | 4(n=3) | 6(n=3) | 8(n=3) | 10(n=3) |
| --- | --- | --- | --- | --- | --- | --- | --- |
| Temperature（℃） | BSF Vermicompost | 28.3(0.26) | 38.3(0.31) | 43.4(0.36) | 47.0(0.66) | 49.9(0.25) | 43.6(0.38) |
|  | Natural Compst | 28.3(0.26) | 33.9(0.25) | 34.6(0.32) | 46.2(0.26) | 45.6(0.35) | 38.4(0.25) |
| Moisture（%） | BSF Vermicompost | 61.3(0.58) | 53.0(0.10) | 47.6(0.26) | 41.1(0.06) | 40.0(0.15) | 28.9(0.25) |
|  | Natural Compst | 61.3(0.58) | 58.4(0.12) | 56.6(0.15) | 55.1(0.10) | 41.2(0.06) | 33.6(0.06) |
| PH | BSF Vermicompost | 3.68(0.02) | 6.86(0.02) | 6.09(0.0058) | 5.90(0.020) | 6.17(0.017) | 6.08(0.030) |
|  | Natural Compst | 3.68(0.015) | 5.92(0.026) | 5.41(0.006) | 7.05(0.006) | 7.11(0.015) | 7.60(0.026) |
| EC (ms/cm) | BSF Vermicompost | 9.83(0.015) | 9.31(0.0025) | 9.56(0.042) | 10.20(0.053) | 10.54(0.021) | 10.86(0.025) |
|  | Natural Compst | 9.83(0.015) | 9.92(0.003) | 9.93(0.003) | 10.58(0.015) | 10.51(0.078) | 10.41(0.040) |
| Carbon(g/kg) | BSF Vermicompost | 465.3(4.75) | 443.3(2.73) | 410.2(4.08) | 403.1(8.24) | 394.0(1.21) | 389.3(6.44) |
|  | Natural Compst | 465.3(4.8) | 462.9(2.3) | 458.8(15.2) | 458.2(2.9) | 448.9(4.6) | 436.8(8.8) |
| Hydrogen(g/kg) | BSF Vermicompost | 72.7(0.98) | 67.8(0.80) | 61.1(0.84) | 60.6(1.23) | 56.9(4.05) | 57.0 (0.34) |
|  | Natural Compst | 72.7(0.98) | 74.2(0.28) | 73.3(0.53) | 73.8(0.53) | 69.9(0.59) | 66.5(1.60) |
| Nitrogen(g/kg) | BSF Vermicompost | 32.4(0.44) | 17.72(0.12) | 19.04(0.09) | 14.78(0.53) | 17.62(0.50) | 20.17(0.60) |
|  | Natural Compst | 32.4(0.44) | 35.3(0.21) | 34.8(0.32) | 30.9(0.28) | 29.7(0.34) | 26.2(0.61) |
| Ammonium(mg/kg) | BSF Vermicompost | 99.22(1.03) | 299.9(0.28) | 66.49(0.08) | 135.0(0.49) | 171.4(0.08) | 108.5(0.41) |
|  | Natural Compst | 99.2(1.03) | 121.6(0.47) | 539.6(10.60) | 311.6(0.27) | 622.2(7.37) | 679.8(25.53) |
| Sulfur (g/kg) | BSF Vermicompost | 6.63(0.59) | 4.40(0.13) | 4.02(0.03) | 3.47(0.27) | 3.20(0.50) | 2.80(0.04) |
|  | Natural Compst | 6.63(0.591) | 6.60(0.192) | 6.15(0.432) | 5.61(0.081) | 5.27(0.023) | 5.05(0.265) |
| Phosphorus(g/kg) | BSF Vermicompost | 5.31(0.020) | 4.11(0.02) | 3.73(0.03) | 3.63(0.03) | 4.15(0.0077) | 4.9(0.020) |
|  | Natural Compst | 5.31(0.020) | 5.51(0.043) | 5.73(0.073) | 6.61(0.008) | 7.03(0.061) | 8.11(0.043) |
| Available phosphorus(mg/kg) | BSF Vermicompost | 816.8(5.27) | 512.0(2.27) | 390.5(2.00) | 266.4(3.30) | 387.4(1.31) | 366.0(4.21) |
|  | Natural Compst | 816.8(5.27) | 507.0(1.52) | 613.2(4.02) | 608.8(2.63) | 607.0(1.52) | 603.5(2.63) |
| Beta glycosidase(U/g) | BSF Vermicompost | 76.09(2.51) | 163.7(17.5) | 193.1(3.29) | 223.8(10.2) | 235.0(11.5) | 295.8(19.6) |
|  | Natural Compst | 76.09(2.51) | 69.27(2.71) | 78.96(2.49) | 54.78(2.39) | 48.80(5.31) | 38.62(2.99) |
| Urease(U/g) | Natural Compst | 7.35(1.64) | 276.7(55.1) | 1962(46.6) | 5508(407) | 3564(174) | 12105(1064) |
|  | BSF Vermicompost | 7.35(1.64) | 5.39(1.76) | 9.03(0.56) | 69.58(8.00) | 56.35(2.87) | 78.19(19.16) |
| Phosphatase(U/g) | BSF Vermicompost | 13028(267) | 13639(2508) | 15339(1974) | 19250(2159) | 17144(558) | 17322(1626) |
|  | Natural Compst | 13028(267) | 10900(50.0) | 12161(404) | 11133(1603) | 10950(117) | 11800(100) |
| The weight of compost (kg, dry) | BSF Vermicompost | 9.65(0) | - | - | - | - | 5.91(0.22) |
|  | Natural Compst | 9.65(0) | - | - | - | - | 8.68(0.10) |
| Prepupal weight (mg) | BSF Vermicompost | 1.16(0.016) | 26.1(1.53) | 63.1(2.65) | 117.7(4.16) | 176(2.08) | 215.2(7.50) |

**Table S2** Detected [microorganism](javascript:;) at phylum, family, order, genus and species level (only list item whose average relative abundance is more than 1%) in raw food waste, BSF vermicompost, and BSF gut as determined by 16S rRNA sequence analyses. The data are expressed as mean percent (standard error). Values followed with different letters within the same column are significantly different at p < 0.05 by Fisher's Least Significant Difference (LSD) test. Orangered and green block correspond indicate the relative abundance of bacteria in NC is significantly under BC and BG, respectively. Blue block represents the relative abundance of bacteria in NC higher than BC. n represents the number of repetitions

| Phylum | NC(BC0)(n=3) | NC(n=15) | BC(n=15) | BG(n=18) |
| --- | --- | --- | --- | --- |
| Unassigned | 0.27(0.13)^a^ | 0.38(0.12)^a^ | 0.18(0.05)^a^ | 2.51(1.95)^a^ |
| Actinobacteria | 3.48(0.5)^b^ | 2.7(0.33)^b^ | 41.4(7.72)^a^ | 3.76(1.24)^b^ |
| Bacteroidetes | 2.1(0.55)^b^ | 0.69(0.19)^b^ | 0.26(0.07)^b^ | 20.4(9.04)^a^ |
| Firmicutes | 91.2(1.39)^a^ | 88.3(1.46^a^) | 52.3(7.21)^c^ | 68(8.96)^b^ |
| Proteobacteria | 2.52(0.56)^a^ | 7.64(1.59)^a^ | 5.72(2.58)^a^ | 4.23(1.85)^a^ |
| Tenericutes | 0.15(0.06)^b^ | 0.17(0.03)^b^ | 0.11(0.02)^b^ | 0.76(0.29)^a^ |
| Others | 0.26(0.11)^a^ | 0.09(0.02)^ab^ | 0.03(0.01)^b^ | 0.3(0.21)^a^ |

| Class | Order | NC(BC) 0 (n=3) | NC(n=15) | BC(n=15) | BG(n=18) |
| --- | --- | --- | --- | --- | --- |
| Actinobacteria | Actinomycetales | 3.43(0.46)b | 2.68(0.33)b | 41.36(7.72)a | 3.66(1.24)b |
| Bacteroidia | Bacteroidales | 1.85(0.47)b | 0.62(0.18)b | 0.24(0.07)b | 20.20(9.04)a |
| Bacilli | Bacillales | 31.27(21.83)ab | 49.09(5.91)a | 23.92(9.95)b | 46.52(14.15)a |
| Bacilli | Lactobacillales | 56.63(21.52)a | 34.07(5.90)ab | 25.85(6.70)b | 20.14(7.17)b |
| Clostridia | Clostridiales | 3.23(0.70)ab | 5.03(0.42)a | 2.52(0.67)b | 1.28(0.17)c |
| Gammaproteobacteria | Alteromonadales | 0.17(0.04)b | 2.50(0.62)a | 0.26(0.06)b | 0.13(0.03)b |
| Gammaproteobacteria | Enterobacteriales | 0.51(0.05)a | 3.56(1.23)a | 4.90(2.59)a | 2.11(0.95)a |
| Others | Others | 2.64(0.76)ab | 2.07(0.15)ab | 0.76(0.16)b | 3.44(1.54)a |
| Unassigned | Unassigned | 0.27(0.13)b | 0.38(0.12)b | 0.18(0.05)b | 2.53(1.94)a |

| Order | Family | NC(BC)0(n=3) | NC(n=15) | BC(n=15) | BG(n=18) |
| --- | --- | --- | --- | --- | --- |
| Actinomycetales | Corynebacteriaceae | 2.37 (0.32)^b^ | 1.66 (0.26)^b^ | 40.4 (7.78)^a^ | 2.14 (0.95)^b^ |
| Bacteroidales | Porphyromonadaceae | 0.60 (0.15)^b^ | 0.34 (0.11)^b^ | 0.21 (0.06)^b^ | 19.8 (9.07)^a^ |
| Bacillales | Bacillales_Unknown | 1.43 (0.98)^b^ | 4.21 (1.25)^a^ | 0.40 (0.03)^b^ | 1.48 (0.65)^b^ |
| Bacillales | Bacillaceae | 21.83 (15.09)^a^ | 35.1 (4.32)^a^ | 21.2 (9.75)^a^ | 29.2 (10.0)^a^ |
| Bacillales | Planococcaceae | 6.90 (4.95)^ab^ | 8.67 (1.21)^a^ | 2.06 (0.37)^b^ | 14.6 (5.59)^a^ |
| Lactobacillales | Lactobacillales_Unknown | 2.03 (0.77)^a^ | 0.86 (0.16)^b^ | 0.54 (0.13)^b^ | 0.19 (0.05)^c^ |
| Lactobacillales | Enterococcaceae | 1.03 (0.37)^c^ | 1.35 (0.35)^c^ | 8.57 (4.16)^b^ | 17.13 (6.59)^a^ |
| Lactobacillales | Lactobacillaceae | 51.43 (20.59)^a^ | 30.9 (5.74)^b^ | 16.3 (2.53)^c^ | 2.31 (0.42)^d^ |
| Clostridiales | [Tissierellaceae] | 0.93 (0.38)^ab^ | 1.12 (0.25)^ab^ | 1.62 (0.82)^a^ | 0.43 (0.08)^b^ |
| Clostridiales | Clostridiaceae | 1.03 (0.38)^ab^ | 1.51 (0.20)^a^ | 0.37 (0.11)^b^ | 0.51 (0.16)^b^ |
| Clostridiales | Lachnospiraceae | 0.53 (0.09)^b^ | 1.60 (0.34)^a^ | 0.33 (0.15)^b^ | 0.21 (0.05)^b^ |
| Alteromonadales | Shewanellaceae | 0.13 (0.03)^b^ | 2.49 (0.63)^a^ | 0.25 (0.06)^b^ | 0.12 (0.03)^b^ |
| Enterobacteriales | Enterobacteriaceae | 0.53 (0.07)^b^ | 3.55 (1.23)^a^ | 4.89 (2.60)^a^ | 2.11 (0.95)^a^ |
| Others | Others | 7.70 (1.78)^a^ | 5.10 (0.27)^ab^ | 2.46 (0.53)^b^ | 4.68 (2.52)^ab^ |

| Family | Genus | NC(BC)0(n=3) | NC(n=15) | BC(n=15) | BG(n=18) |
| --- | --- | --- | --- | --- | --- |
| Corynebacteriaceae | Corynebacterium | 2.38(0.31 )^b^ | 1.66(0.25 )^b^ | 40.4(7.78 )^a^ | 2.15(0.95 )^b^ |
| Porphyromonadaceae | Dysgonomonas | 0.59(0.15 )^b^ | 0.34(0.11 )^b^ | 0.21(0.07 )^b^ | 19.8(9.07 )^a^ |
| Bacillales_Unknown_f | Bacillales_Unknown_g | 1.43(1.00 )^b^ | 4.20(1.24 )^a^ | 0.40(0.03 )^b^ | 1.48(0.65 )^b^ |
| Bacillaceae | Bacillaceae_Unknown | 2.90(1.71 )^b^ | 8.81(1.47 )^a^ | 1.18(0.31 )^b^ | 2.32(0.64 )^b^ |
| Bacillaceae | Bacillus | 17.4(12.7 )^a^ | 17.7(5.19 )^a^ | 19.2(9.38 )^a^ | 22.3(11.2 )^a^ |
| Bacillaceae | Virgibacillus | 0.42(0.15 )^c^ | 4.56(1.41 )^a^ | 0.35(0.09 )^c^ | 2.60(1.63 )^ab^ |
| Bacillaceae | Bacillaceae_Other | 0.93(0.50 )^ab^ | 3.62(1.13 )^a^ | 0.39(0.07 )^b^ | 1.86(0.94 )^ab^ |
| Planococcaceae | Planococcaceae_Unknown | 2.62(1.56 )^ab^ | 4.78(1.61 )^ab^ | 0.96(0.21 )^b^ | 7.55(4.85 )^a^ |
| Planococcaceae | Rummeliibacillus | 2.45(2.28 )^a^ | 1.34(0.40 )^a^ | 0.21(0.06 )^b^ | 0.07(0.01 )^b^ |
| Planococcaceae | Sporosarcina | 0.35(0.17 )^ab^ | 1.58(0.80 )^a^ | 0.06(0.02 )^b^ | 0.27(0.13 )^b^ |
| Planococcaceae | Ureibacillus | 0.38(0.20 )^b^ | 0.35(0.06 )^c^ | 0.69(0.25 )^b^ | 6.56(3.57 )^a^ |
| Lactobacillales_Unknown | Lactobacillales_Unknown | 2.04(0.77 )^a^ | 0.86(0.16 )^b^ | 0.54(0.13 )^b^ | 0.19(0.05 )^c^ |
| Enterococcaceae | Enterococcus | 0.93(0.34 )^c^ | 1.17(0.27 )^c^ | 6.30(3.04 )^b^ | 16.5(6.28 )^a^ |
| Enterococcaceae | Vagococcus | 0.08(0.02 )^b^ | 0.18(0.09 )^b^ | 2.25(1.14 )^a^ | 0.56(0.40 )^b^ |
| Lactobacillaceae | Lactobacillus | 43.7(17.18 )^a^ | 26.6(4.8 )^b^ | 14.2(2.05 )^c^ | 2.02(0.39 )d |
| Lactobacillaceae | Lactobacillaceae_Other | 7.03(3.11 )^a^ | 4.14(0.90 )^b^ | 2.02(0.56 )^c^ | 0.28(0.04 )d |
| [Tissierellaceae] | Anaerococcus | 0.30(0.10 )^ab^ | 0.13(0.04 )^b^ | 1.48(0.80 )^a^ | 0.09(0.03 )^b^ |
| Clostridiaceae | Clostridium | 0.50(0.02 )^ab^ | 1.11(0.22 )^a^ | 0.29(0.10 )^b^ | 0.41(0.13 )^b^ |
| Shewanellaceae | Shewanella | 0.14(0.04 )^b^ | 2.48(0.63 )^a^ | 0.25(0.06 )^b^ | 0.13(0.03 )^b^ |
| Enterobacteriaceae | Proteus | 0.14(0.09 )^b^ | 2.63(1.02 )^a^ | 0.31(0.14 )^b^ | 0.21(0.03 )^b^ |
| Enterobacteriaceae | Providencia | 0.06(0.02 )^b^ | 0.68(0.30 )^b^ | 4.42(2.51 )^a^ | 1.70(0.89 )^b^ |
| RsaHF231_Unknown_f | RsaHF231_Unknown_g | 0.14(0.02)^b^ | 0.15(0.03)^b^ | 0.11(0.05)^b^ | 0.70(0.03)^a^ |
| Unassigned | Unassigned | 0.27(0.13 )^b^ | 0.38(0.12 )^b^ | 0.18(0.05 )^b^ | 2.53(1.94 )^a^ |

| Genus | Species | NC(BC)0(n=3) | NC(n=15) | BC(n=15) | BG(n=18) |
| --- | --- | --- | --- | --- | --- |
| Corynebacterium | Corynebacterium_Unknown | 2.24 (0.28)^b^ | 1.35 (0.30)^b^ | 34.2 (7.40)^a^ | 1.38 (0.45)^b^ |
| Corynebacterium | variabile | 0.15 (0.04)^b^ | 0.31 (0.07)^b^ | 6.14 (2.76)^a^ | 0.77 (0.59)^b^ |
| Dysgonomonas | Dysgonomonas_Unknown | 0.59 (0.15)^b^ | 0.34 (0.11)^b^ | 0.21 (0.07)^b^ | 19.8 (9.07)^a^ |
| Bacillales_Unknown | Bacillales_Unknown_Unknown | 1.43 (1.00)^b^ | 4.20 (1.24)^a^ | 0.40 (0.03)^b^ | 1.48 (0.65)^b^ |
| Bacillaceae_Unknown | Bacillaceae_Unknown | 2.90 (1.71)^b^ | 8.81 (1.47)^a^ | 1.18 (0.31)^b^ | 2.32 (0.64)^b^ |
| Bacillus | Bacillus_Other | 1.49 (1.10)^a^ | 1.28 (0.43)^a^ | 1.06 (0.49)^a^ | 0.94 (0.39)^a^ |
| Bacillus | Bacillus_Unknown | 12.1 (10.2)^a^ | 12.1 (4.62)^a^ | 1.18 (0.24)^b^ | 2.17 (0.63)^b^ |
| Bacillus | coagulans | 1.38 (0.72)^b^ | 2.07 (0.20)^b^ | 5.50 (2.83)^a^ | 3.02 (1.64)^ab^ |
| Bacillus | thermoamylovorans | 2.04 (0.34)^c^ | 1.78 (0.20)^c^ | 11.4 (6.21)^b^ | 16.1 (9.11)^a^ |
| Virgibacillus | Virgibacillus_Other | 0.06 (0.03)^b^ | 2.74 (1.31)^a^ | 0.11 (0.03)^b^ | 0.29 (0.17)^b^ |
| Virgibacillus | Virgibacillus_Unknown | 0.35 (0.12)^b^ | 1.82 (0.35)^ab^ | 0.24 (0.07)^b^ | 2.31 (1.65)^a^ |
| Bacillaceae_Other | Bacillaceae_Other | 0.93 (0.50)^b^ | 3.62 (1.13)^a^ | 0.39 (0.07)^b^ | 1.86 (0.94)^ab^ |
| Planococcaceae_Unknown | Planococcaceae_Unknown | 2.62 (1.56)^b^ | 4.78 (1.61)^ab^ | 0.96 (0.21)^b^ | 7.55 (4.85)^a^ |
| Rummeliibacillus | Rummeliibacillus_Unknown | 2.45 (2.28)^a^ | 1.34 (0.40)^a^ | 0.21 (0.06)^b^ | 0.07 (0.01)^b^ |
| Ureibacillus | Ureibacillus_Unknown | 0.38 (0.20)^b^ | 0.35 (0.06)^b^ | 0.69 (0.25)^b^ | 6.56 (3.57)^a^ |
| Lactobacillales_Unknown | Lactobacillales_Unknown | 2.04 (0.77)^a^ | 0.86 (0.16)^b^ | 0.54 (0.13)^b^ | 0.19 (0.05)^c^ |
| Enterococcus | Enterococcus_Other | 0.51 (0.22)^c^ | 0.84 (0.20)^c^ | 3.35 (1.74)^b^ | 15.4 (6.13)^a^ |
| Enterococcus | Enterococcus_Unknown | 0.23 (0.07)^b^ | 0.28 (0.08)^b^ | 2.70 (1.32)^a^ | 0.90 (0.29)^ab^ |
| Vagococcus | Vagococcus_Unknown | 0.08 (0.02)^a^ | 0.17 (0.09)^a^ | 2.25 (1.14)^a^ | 0.56 (0.40)^a^ |
| Lactobacillus | Lactobacillus_Unknown | 39.6 (15.5)^a^ | 24.0 (4.29)^b^ | 12.4 (1.73)^c^ | 1.80 (0.34)^d^ |
| Lactobacillus | brevis | 3.49 (1.51)^a^ | 2.16 (0.51)^a^ | 0.94 (0.28)^b^ | 0.14 (0.02)^b^ |
| Lactobacillaceae_Other | Lactobacillaceae_Other | 7.03 (3.11)^a^ | 4.14 (0.90)^a^ | 2.02 (0.56)^b^ | 0.28 (0.04)^c^ |
| Anaerococcus | Anaerococcus_Unknown | 0.30 (0.10)^ab^ | 0.13 (0.04)^b^ | 1.48 (0.80)^a^ | 0.09 (0.03)^b^ |
| Shewanella | algae | 0.13 (0.04)^b^ | 2.44 (0.62)^a^ | 0.25 (0.06)^b^ | 0.12 (0.03)^b^ |
| Proteus | Proteus_Unknown | 0.14 (0.09)^b^ | 2.63 (1.02)^a^ | 0.31 (0.14)^b^ | 0.21 (0.03)^b^ |
| Providencia | Providencia_Other | 0.06 (0.02)^b^ | 0.67 (0.30)^b^ | 4.41 (2.50)^a^ | 1.67 (0.88)^b^ |
| Unassigned | Unassigned | 0.27 (0.13)^b^ | 0.38 (0.12)^b^ | 0.18 (0.05)^b^ | 2.53 (1.94)^a^ |
| Others | Others | 15.1 (1.02)^a^ | 14.3 (1.29)^a^ | 5.25 (0.63)^b^ | 9.48 (2.64)^ab^ |

**Table S3** Numbers of normalized reads for each shared and exclusively detected OTU within the indicated segments of the Venn diagram.

| Treatment -Day | Total number  (OTUs kind) | Shared Type (kind of OTUs) | Co-shared OTUs number  (proportion of the total OTUs number) | Family | Number(percent) |
| --- | --- | --- | --- | --- | --- |
| RFW | 50950(622) | Co-shared by RFW, BG0 and BC10 （189） | 43430(84.6%) | Bacillaceae | 7894(18.2%) |
|  |  |  |  | Lactobacillaceae | 27882(64.2%) |
| BG0 | 53627(370) |  | 45964(85.7%) | Bacillaceae | 7158(15.6%) |
|  |  |  |  | Enterococcaceae | 23235(50.1%) |
| BC10 | 52168(514) |  | 49383(94.7%) | Corynebacteriaceae | 10957(22.2%) |
|  |  |  |  | Bacillaceae | 27890(56.5%) |
|  |  |  |  | Lactobacillaceae | 6041(12.2%) |
| Exclusively detected in BC samples compared to RFW and BG0(129) | | | 1798(3.4%) | Corynebacteriaceae | 1490(82.9%) |
| RFW | 50950(622) | Co-shared by RFW, BG0 and BG10 （128） | 36699(72.3%) | Bacillaceae | 7463(20.3%) |
|  |  |  |  | Lactobacillaceae | 24254(66.1%) |
| BG0 | 53627(370) |  | 42193(78.7%) | Enterococcaceae | 22026(52.2%) |
|  |  |  |  | Bacillaceae | 5822(13.8%) |
| BG10 | 57989(514) |  | 53063(91.5%) | Corynebacteriaceae | 10598(22.4%) |
|  |  |  |  | Bacillaceae | 27750(58.5%) |
|  |  |  |  | Lactobacillaceae | 4990(10.5%) |
| Exclusively detected in BG10 samples compared to RFW and BG0(84) | | | 1259(2.2%) | Bacillaceae | 939(74.6%) |
|  |  |  |  | Planococcaceae | 123(9.8%) |
| RFW | 50950(622) | Co-shared by RFW and NC10（299） | 49441(97.0%) | Bacillaceae | 9710（19.6%） |
|  |  |  |  | Lactobacillaceae | 28841（58.3%） |
| NC10 | 50944(800) |  | 46520(91.3%) | Bacillaceae | 18607（40.0%） |
|  |  |  |  | Lactobacillaceae | 13643（29.3%） |
| Exclusively detected in NC samples compared to RFW (401) | | | 4424(8.7%) | Bacillaceae | 3037（68.6%） |
|  |  |  |  | Planococcaceae | 215（4.90%） |
| RFW | 50950(622) | Co-shared by BC10, BG0, RFW and NC10（174） | 43139（84.7%） | Bacillaceae | 8391（19.5%） |
|  |  |  |  | Lactobacillaceae | 27882（64.6%） |
| NC10 | 50944(800) |  | 34400（68.1%） | Bacillaceae | 13789（40.1%） |
|  |  |  |  | Lactobacillaceae | 13299（38.7） |
| BC10 | 52168(514) |  | 49090（94.1%） | Bacillaceae | 27928（56.9%） |
|  |  |  |  | Corynebacteriaceae | 10895（22.2%） |

**Table S4** The average relative abundance of metabolic pathway based on FOAM database. The data are expressed as mean percent (standard error). Values followed with different letters within the same column are significantly different at *p* < 0.05 by Fisher's Least Significant Difference (LSD) test. Orangered and green block correspond indicate the relative abundance of bacteria in NC is significantly lower than BC and BG, respectively. Blue block represents the relative abundance of bacteria in NC higher than BC. Yellow represents the relative abundance of bacteria combined BC with BG is higher than NC. n represents the number of repetitions.

| Pathway (Level 1) | NC(n=18) | BC(n=18) | BG(n=18) | BC+BG(n=18) |
| --- | --- | --- | --- | --- |
| Fermentation | 1.16(0.0224)b | 1.14(0.0226)b | 1.02(0.0230)c | 2.16(0.0340)a |
| Homoacetogenesis | 0.06(0.0031)c | 0.07(0.0059)b | 0.08(0.0064)b | 0.16(0.0042)a |
| Thiosulfate metabolism | 0.017(0.0010)c | 0.019(0.0013)c | 0.023(0.0023)b | 0.04(0.0028)a |
| Utililization of sugar (pentose to EMP) | 0.10(0.0032)c | 0.11(0.0017)b | 0.09(0.0044)d | 0.21(0.0048)a |
| Fatty acid oxidation | 0.29(0.0072)c | 0.38(0.034)b | 0.32(0.021)c | 0.70(0.045)a |
| Amino acid utilization biosynthesis metabolism | 1.13(0.0083)c | 1.22(0.038)b | 1.23(0.050)b | 2.46(0.080)a |
| Nucleic acid metabolism | 0.107(0.0010)c | 0.115(0.0019)b | 0.099(0.0025)d | 0.21(0.0032)a |
| Hydrocarbon degradation | 0.094(0.0031)b | 0.091(0.0063)b | 0.10(0.0089)b | 0.19(0.0129)a |
| Carbohydrate Active enzyme | 0.052(0.0011)c | 0.057(0.0010)b | 0.054(0.0037)bc | 0.11(0.0044)a |
| NCA cycle | 0.14(0.0034)d | 0.17(0.0116)c | 0.20(0.0135)b | 0.37(0.0221)a |
| Nitrogen cycle | 0.10(0.0049)c | 0.11(0.0037)bc | 0.12(0.0114)b | 0.23(0.0129)a |
| Transporters | 4.68(0.0895)b | 4.52(0.1125)b | 4.20(0.1521)c | 8.72(0.1791)a |
| Hydrogen metabolism | 0.0045(0.0003)c | 0.0097(0.0019)b | 0.0050(0.00088)c | 0.015(0.0019)a |
| Methanogenesis | 0.0341(0.0012)b | 0.028(0.0011)c | 0.0344(0.0019)b | 0.062(0.0023)a |
| Methylotrophy | 0.302(0.0015)b | 0.299(0.0038)b | 0.293(0.0079)b | 0.59(0.0108)a |
| Embden Meyerhof - Parnos | 0.20(0.0064)c | 0.23(0.0071)b | 0.17(0.0083)d | 0.40(0.0114)a |
| Gluconeogenesis | 0.075(0.0032)b | 0.079(0.0022)b | 0.059(0.0021)c | 0.14(0.0037)a |
| Sulfur compounds metabolism | 0.15(0.0031)c | 0.22(0.0183)b | 0.15(0.0068)c | 0.37(0.0193)a |
| Saccharide and derivated synthesis | 0.24(0.0071)b | 0.23(0.0130)b | 0.25(0.0272)b | 0.48(0.0312)a |
| Hydrolysis of polymers | 0.14(0.0022)b | 0.11(0.0108)c | 0.15(0.0153)b | 0.26(0.0167)a |
| Cellular.response.to.stress | 0.24(0.0010)d | 0.28(0.0111)b | 0.26(0.0039)c | 0.54(0.0112)a |

**Table S5** The relative abundance of genes involved in the metabolism of carbon, nitrogen and sulfur in different groups, based on KEGG database. The data are expressed as mean percent (standard error). Values followed with different letters within the same column are significantly different at p < 0.05 by Fisher's Least Significant Difference (LSD) test. Orangered and green block correspond indicate the relative abundance of bacteria in NC is significantly lower than BC and BG, respectively. Blue block represents the relative abundance of bacteria in NC higher than BC. Yellow represents the relative abundance of bacteria combined BC with BG is higher than NC. n represents the number of repetitions.

| Gene | NC(n=18) | BC(n=18) | BG(n=18) | BC+BG(n=18) |
| --- | --- | --- | --- | --- |
| Enzyme activity of gene | | | | |
| PHO | 8.18E-04(6.31E-05)c | 1.55E-03(4.14E-04)b | 8.78E-04(3.96E-04)bc | 2.43E-03(4.40E-04)a |
| appA | 1.19E-03(1.50E-04)ab | 6.75E-04(2.40E-04)b | 9.49E-04(3.65E-04)b | 1.62E-03(5.27E-04)a |
| aphA | 2.40E-03(7.99E-04)b | 3.03E-03(1.42E-03)ab | 2.26E-03(1.36E-03)b | 5.29E-03(2.64E-03)a |
| phoN | 6.94E-04(1.44E-04)ab | 4.07E-04(1.85E-04)b | 4.81E-04(2.10E-04)ab | 8.89E-04(3.67E-04)a |
| bglB | 0.026(1.59E-03)bc | 0.023(3.44E-03)c | 0.034(5.43E-03)b | 0.057(7.66E-03)a |
| E3.2.1.21 | 0.027(2.77E-03)c | 0.024(5.10E-03)c | 0.054(0.011)b | 0.078(0.010)a |
| ureC | 3.93E-03(6.71E-04)b | 4.83E-03(1.56E-03)b | 4.13E-03(1.20E-03)b | 8.96E-03(1.58E-03)a |
| ureB | 3.57E-03(6.92E-04)b | 2.05E-03(4.79E-04)b | 3.68E-03(9.96E-04)b | 5.74E-03(1.20E-03)a |
| ureAB | 1.25E-04(2.46E-05)b | 6.77E-05(3.07E-05)b | 1.57E-04(1.06E-04)b | 2.25E-04(1.35E-04)a |
| The gene of nitrogen metabolism | | | | |
| narG | 8.85E-03(4.89E-04)a | 6.41E-03(8.43E-04)b | 1.93E-03(5.17E-04)c | 8.35E-03(1.29E-03)a |
| narH | 8.87E-03(4.89E-04)a | 6.42E-03(8.44E-04)c | 1.96E-03(5.18E-04)d | 8.38E-03(1.30E-03)a |
| narI | 8.93E-03(4.97E-04)a | 6.46E-03(8.47E-04)b | 2.08E-03(5.34E-04)c | 8.55E-03(1.34E-03)a |
| napA | 3.26E-03(8.92E-04)a | 1.33E-03(2.64E-04)b | 2.48E-03(1.30E-03)ab | 3.81E-03(1.33E-03)a |
| napB | 3.18E-03(9.09E-04)a | 1.28E-03(2.80E-04)b | 2.40E-03(1.30E-03)ab | 3.68E-03(1.33E-03)a |
| nrfA | 2.88E-03(4.69E-04)b | 1.65E-03(2.28E-04)b | 1.47E-02(6.15E-03)a | 1.63E-02(0.0063)a |
| nirB | 1.50E-02(3.23E-03)a | 6.51E-03(1.61E-03)c | 8.02E-03(1.83E-03)c | 1.45E-02(3.02E-03)ab |
| nirD | 8.11E-03(1.47E-03)a | 3.50E-03(8.58E-04)b | 4.61E-03(2.20E-03)b | 8.11E-03(2.62E-03)a |
| nirA | 8.82E-03(9.53E-04)b | 3.37E-02(7.13E-03)a | 5.30E-03(1.62E-03)b | 3.90E-02(0.0059)a |
| nasA | 6.06E-03(7.33E-04)ab | 3.19E-03(8.25E-04)c | 3.68E-03(1.64E-03)bc | 6.87E-03(2.09E-03)a |
| The gene of sulfur metabolism | | | | |
| Sat | 0.020(1.50E-03)bc | 9.73E-03(3.07E-03)c | 0.025(8.06E-03)ab | 0.035(0.010)a |
| CysNC | 3.45E-03(2.38E-04)ab | 1.75E-03(5.34E-04)b | 3.01E-03(1.40E-03)ab | 4.77E-03(1.83E-03)a |
| CysN | 9.33E-03(8.87E-04)c | 0.036(7.09E-03)b | 8.26E-03(3.32E-03)c | 0.044(4.86E-03)a |
| CysD | 7.73E-03(9.77E-04)c | 0.035(7.32E-03)b | 7.43E-03(3.01E-03)c | 0.043(5.30E-03)a |
| CysC | 0.022(1.73E-03)b | 0.013(2.67E-03)c | 0.026(7.40E-03)b | 0.039(8.90E-03)a |
| CysH | 0.029(3.12E-03)c | 0.047(6.01E-03)b | 0.031(6.22E-03)c | 0.078(0.010)a |
| CysJ | 0.011(1.16E-03)c | 9.85E-03(2.40E-03)c | 0.022(6.13E-03)b | 0.032(7.79E-03)a |
| CysI | 0.010(9.29E-04)c | 9.69E-03(2.40E-03)c | 0.022(6.07E-03)b | 0.032(7.74E-03)a |
| The gene of carbon metabolism(grouping according to the metabolic pathway in KEGG) | | | | |
| Cellulose-cellobiose | 0.056(0.0028)c | 0.047(0.0069）c | 0.14(0.040)b | 0.18(0.041)a |
| Starch/detrin/maltodexin-maltose | 0.053(0.0017)c | 0.11(0.023)b | 0.067(0.0099)c | 0.18(0.028)a |
| Detrin/maltodexin/maltose-glucose | 0.15(0.0052）b | 0.093(0.015)c | 0.16(0.022)b | 0.26(0.020)a |
| Glucose-NCA | 1.09(0.028)c | 1.19(0.017)b | 1.01(0.020)d | 2.20(0.024)a |
| NCA | 0.44(0.028)c | 0.46(0.038)c | 0.72(0.055)b | 1.18(0.079)a |
| The gene of carbon metabolism | | | | |
| bglX | 2.69E-02(1.90E-03)b | 2.29E-02(3.75E-03)b | 1.00E-01(4.47E-02)a | 1.23E-01(4.55E-02)a |
| bglB | 2.61E-02(1.59E-03)bc | 2.26E-02(3.44E-03)c | 3.41E-02(5.43E-03)b | 5.67E-02(7.66E-03)a |
| AMY | 4.11E-02(1.64E-03)b | 4.81E-02(7.05E-03)b | 4.86E-02(4.51E-03)b | 9.67E-02(1.07E-02)a |
| cd | 2.21E-03(3.12E-04)b | 2.61E-02(8.09E-03)a | 1.43E-03(5.10E-04)b | 2.75E-02(7.88E-03)a |
| IMA | 4.38E-02(2.62E-03)b | 2.52E-02(3.43E-03)c | 3.21E-02(6.07E-03)c | 5.73E-02(7.60E-03)a |
| malZ | 1.02E-01(6.02E-03)b | 6.74E-02(1.23E-02)c | 1.31E-01(2.40E-02)b | 1.98E-01(2.23E-02)a |
| HK | 7.87E-02(3.46E-03)b | 8.29E-02(3.72E-03)b | 8.42E-02(1.50E-02)b | 1.67E-01(1.57E-02)a |
| ppgK | 4.33E-03(5.39E-04)b | 3.40E-02(7.42E-03)a | 4.67E-03(1.38E-03)b | 3.87E-02(6.42E-03)a |
| GPI | 6.52E-02(2.87E-03)c | 7.67E-02(1.62E-03)b | 5.99E-02(2.27E-03)c | 1.37E-01(2.80E-03)a |
| pfkA | 5.92E-02(8.14E-04)c | 7.13E-02(2.76E-03)b | 6.12E-02(2.31E-03)c | 1.33E-01(2.94E-03)a |
| ALDO | 1.06E-02(2.18E-03)c | 2.71E-02(6.77E-03)b | 2.48E-02(7.23E-03)b | 5.20E-02(7.18E-03)a |
| FBA | 6.48E-02(1.09E-03)b | 5.41E-02(5.61E-03)c | 6.81E-02(4.05E-03)b | 1.22E-01(9.42E-03)a |
| PGAM | 3.62E-01(2.51E-02)b | 3.50E-01(2.12E-02)b | 2.19E-01(1.09E-02)c | 5.69E-01(2.84E-02)a |
| ENO | 8.08E-02(4.01E-03)b | 8.27E-02(1.90E-03)b | 6.25E-02(2.33E-03)c | 1.45E-01(3.55E-03)a |
| PK | 7.37E-02(1.62E-03)c | 1.03E-01(8.12E-03)b | 6.57E-02(2.78E-03)c | 1.69E-01(7.47E-03)a |
| PDHA | 5.88E-02(1.61E-03)bc | 4.52E-02(5.86E-03)c | 6.98E-02(1.05E-02)b | 1.15E-01(1.33E-02)a |
| PDHB | 5.69E-02(1.05E-03)bc | 4.52E-02(5.82E-03)c | 6.98E-02(1.07E-02)b | 1.15E-01(1.35E-02)a |
| aceE | 9.72E-03(7.43E-04)c | 3.82E-02(7.10E-03)b | 9.16E-03(3.45E-03)c | 4.73E-02(4.68E-03)a |
| DLAT | 6.11E-02(1.58E-03)bc | 4.83E-02(6.39E-03)c | 7.30E-02(1.01E-02)b | 1.21E-01(1.30E-02)a |
| DLAD | 9.33E-02(4.23E-03)c | 1.25E-01(8.60E-03)b | 1.17E-01(2.74E-03)b | 2.41E-01(9.10E-03)a |
| ALDO | 1.06E-02(2.18E-03)c | 2.71E-02(6.77E-03)b | 2.48E-02(7.23E-03)b | 5.20E-02(7.18E-03)a |
| korB | 1.55E-02(1.70E-03)b | 8.57E-03(3.10E-03)b | 4.91E-02(1.21E-02)a | 5.76E-02(1.22E-02)a |
| korA | 1.55E-02(1.70E-03)b | 8.56E-03(3.10E-03)b | 4.90E-02(1.21E-02)a | 5.76E-02(1.22E-02)a |
| IDH1 | 3.35E-02(2.27E-03)c | 5.08E-02(5.26E-03)b | 5.17E-02(3.02E-03)b | 1.03E-01(6.74E-03)a |
| Aco | 3.49E-02(2.17E-03)c | 5.08E-02(5.95E-03)b | 5.40E-02(2.78E-03)b | 1.05E-01(7.24E-03)a |
| sdhA | 3.07E-02(2.58E-03)c | 4.73E-02(6.08E-03)b | 5.34E-02(2.88E-03)b | 1.01E-01(7.61E-03)a |
| sdhC | 2.99E-02(2.63E-03)c | 4.67E-02(6.18E-03)b | 5.26E-02(2.96E-03)b | 9.93E-02(7.97E-03)a |
| sdhB | 3.03E-02(2.61E-03)c | 4.69E-02(6.11E-03)b | 5.32E-02(2.89E-03)b | 1.00E-01(7.67E-03)a |
| sucC | 2.78E-02(2.76E-03)c | 1.61E-02(3.32E-03)d | 4.98E-02(3.36E-03)b | 6.60E-02(4.92E-03)a |
| sucD | 2.78E-02(2.76E-03)c | 1.61E-02(3.31E-03)d | 4.99E-02(3.38E-03)b | 6.61E-02(4.92E-03)a |
| E4.2.1.2A | 1.54E-02(1.49E-03)c | 9.27E-03(2.97E-03)c | 3.92E-02(6.35E-03)b | 4.85E-02(7.91E-03)a |
| fumC | 5.91E-02(1.63E-03)b | 6.47E-02(3.51E-03)b | 4.15E-02(4.32E-03)c | 1.06E-01(5.69E-03)a |
| mdh | 3.38E-02(2.82E-03)d | 4.59E-02(6.01E-03)c | 6.43E-02(3.55E-03)b | 1.10E-01(7.85E-03)a |
| CS | 5.16E-02(3.99E-03)c | 3.52E-02(4.62E-03)d | 6.73E-02(2.25E-03)b | 1.03E-01(5.90E-03)a |

**Table S6** Statistic using mantel test to present the influence of environmental variables in RDA.

| Variables | r | Significance |
| --- | --- | --- |
| Temperature | 0.77 | 0.001 |
| PH | 0.72 | 0.001 |
| Moisture | 0.80 | 0.001 |
| EC | 0.43 | 0.001 |
| Ammonium | 0.15 | 0.11 |
| Nitrogen | 0.61 | 0.001 |
| Carbon | 0.80 | 0.001 |
| Hydrogen | 0.75 | 0.001 |
| Sulfur | 0.84 | 0.001 |
| Phosphorus | 0.56 | 0.001 |
| Olsen phosphorus | 0.78 | 0.001 |
| Urease | 0.343 | 0.012 |
| Phosphatase | 0.29 | 0.006 |
| Beta glycosidase | 0.81 | 0.001 |

Permutation: free; Number of permutations: 999.

**Table S7** Important coefficients for co-occurrence network of BC and BG.

| phylum | class | order | family | name | Modularity  class | Degree | Betweenness  Centrality | Topological  Coefficient |
| --- | --- | --- | --- | --- | --- | --- | --- | --- |
| p__Firmicutes | c__Clostridia | o__Clostridiales | f__Clostridiaceae | v_Alkaliphilus | 0 | 1 | 0 | 0 |
| p__Firmicutes | c__Bacilli | o__Bacillales | f__Bacillaceae | g_Natronobacillus | 0 | 3 | 6.22E-04 | 0.527778 |
| p__Bacteroidetes | c__Bacteroidia | o__Bacteroidales | f__Prevotellaceae | g_Prevotella | 0 | 5 | 0.007697 | 0.347368 |
| p__Actinobacteria | c__Actinobacteria | o__Actinomycetales | f__Microbacteriaceae | g_Microbacterium | 0 | 6 | 9.18E-04 | 0.378205 |
| p__Proteobacteria | c__Gammaproteobacteria | o__Pseudomonadales | f__Moraxellaceae | g_Psychrobacter | 0 | 6 | 0.003473 | 0.381944 |
| p__Firmicutes | c__Clostridia | o__Clostridiales | f__Caldicoprobacteraceae | g_Caldicoprobacter | 0 | 6 | 0.02428 | 0.314103 |
| p__Firmicutes | c__Bacilli | o__Lactobacillales | f__Aerococcaceae | g_Facklamia | 0 | 7 | 0.002791 | 0.322751 |
| p__Firmicutes | c__Bacilli | o__Bacillales | f__Planococcaceae | g_Sporosarcina | 0 | 7 | 0.002791 | 0.322751 |
| p__Firmicutes | c__Clostridia | o__Clostridiales | f__[Tissierellaceae] | v_Tepidimicrobium | 0 | 7 | 0.011955 | 0.315126 |
| p__Actinobacteria | c__Actinobacteria | o__Actinomycetales | f__Actinomycetaceae | g_Actinomyces | 0 | 7 | 0.01808 | 0.28125 |
| p__Firmicutes | c__Bacilli | o__Bacillales | Bacillales_Unknown_f | g_Bacillales_Unknown | 0 | 8 | 0.004955 | 0.219907 |
| p__Firmicutes | c__Bacilli | o__Bacillales | f__Planococcaceae | v_Ureibacillus | 0 | 8 | 0.005628 | 0.335 |
| p__Firmicutes | c__Bacilli | o__Lactobacillales | f__Aerococcaceae | v_Facklamia | 0 | 8 | 0.009515 | 0.276515 |
| p__Proteobacteria | c__Deltaproteobacteria | o__Desulfuromonadales | f__Geobacteraceae | v_Geobacter | 0 | 9 | 0 | 0.563218 |
| p__Firmicutes | c__Bacilli | o__Lactobacillales | f__Streptococcaceae | v_Streptococcus | 0 | 9 | 9.63E-04 | 0.560185 |
| p__Proteobacteria | c__Alphaproteobacteria | o__Rhizobiales | f__Rhizobiaceae | v_Agrobacterium | 0 | 10 | 0.001492 | 0.496296 |
| p__Firmicutes | c__Bacilli | o__Bacillales | f__Thermoactinomycetaceae | g_Thermoactinomycetaceae_Unknown | 0 | 10 | 0.00749 | 0.439394 |
| p__Proteobacteria | c__Gammaproteobacteria | o__Oceanospirillales | f__Alcanivoracaceae | v_Alcanivorax | 0 | 10 | 0.00749 | 0.439394 |
| p__Actinobacteria | c__Actinobacteria | o__Actinomycetales | f__Brevibacteriaceae | v_Brevibacterium | 0 | 10 | 0.008275 | 0.258929 |
| p__Proteobacteria | c__Gammaproteobacteria | o__Xanthomonadales | f__Xanthomonadaceae | g_Lysobacter | 0 | 10 | 0.028221 | 0.231915 |
| p__Firmicutes | c__Bacilli | o__Lactobacillales | f__Lactobacillaceae | v_Pediococcus | 0 | 11 | 0.022881 | 0.310023 |
| p__Firmicutes | c__Clostridia | o__Clostridiales | f__[Tissierellaceae] | v_Tissierella_Soehngenia | 0 | 12 | 0.001037 | 0.436275 |
| p__Proteobacteria | c__Gammaproteobacteria | o__Xanthomonadales | f__Xanthomonadaceae | g_Stenotrophomonas | 0 | 12 | 0.012113 | 0.251938 |
| p__Proteobacteria | c__Gammaproteobacteria | o__Pseudomonadales | f__Moraxellaceae | v_Acinetobacter | 0 | 12 | 0.02953 | 0.200758 |
| p__Cyanobacteria | c__Chloroplast | o__Streptophyta | Streptophyta_Unknown_f | v_Streptophyta_Unknown | 0 | 12 | 0.03723 | 0.253401 |
| p__Proteobacteria | c__Gammaproteobacteria | o__Pseudomonadales | f__Pseudomonadaceae | v_Pseudomonas | 0 | 12 | 0.058897 | 0.201058 |
| Other | Other | Other | Other | v_Other | 0 | 13 | 0.001573 | 0.456731 |
| p__Firmicutes | c__Bacilli | o__Lactobacillales | f__Leuconostocaceae | v_Leuconostoc | 0 | 13 | 0.004749 | 0.426374 |
| p__Proteobacteria | c__Gammaproteobacteria | o__Enterobacteriales | f__Enterobacteriaceae | g_Proteus | 0 | 13 | 0.011881 | 0.448718 |
| p__Firmicutes | c__Clostridia | o__Clostridiales | f__[Tissierellaceae] | v_[Tissierellaceae]_Unknown | 0 | 14 | 0.004189 | 0.368421 |
| p__Bacteroidetes | c__Bacteroidia | o__Bacteroidales | f__Prevotellaceae | v_Prevotella | 0 | 14 | 0.007299 | 0.409664 |
| p__Firmicutes | c__Bacilli | o__Lactobacillales | f__Leuconostocaceae | v_Leuconostocaceae_Other | 0 | 14 | 0.007896 | 0.413866 |
| p__Bacteroidetes | c__Bacteroidia | o__Bacteroidales | f__Bacteroidaceae | v_Bacteroides | 0 | 14 | 0.01613 | 0.312925 |
| p__Proteobacteria | c__Gammaproteobacteria | o__Enterobacteriales | f__Enterobacteriaceae | g_Enterobacteriaceae_Other | 0 | 14 | 0.017039 | 0.231366 |
| p__Firmicutes | c__Bacilli | o__Haloplasmatales | f__Haloplasmataceae | v_Haloplasmataceae_Unknown | 0 | 14 | 0.026388 | 0.315714 |
| p__Firmicutes | c__Clostridia | o__Clostridiales | f__[Tissierellaceae] | v_[Tissierellaceae]_Other | 0 | 15 | 0.030689 | 0.31634 |
| p__Firmicutes | c__Bacilli | o__Bacillales | f__Bacillaceae | g_Bacillaceae_Other | 0 | 15 | 0.032513 | 0.29881 |
| p__Proteobacteria | c__Alphaproteobacteria | o__Rhodospirillales | f__Acetobacteraceae | v_Acetobacter | 0 | 16 | 0.00433 | 0.454861 |
| p__Firmicutes | c__Bacilli | o__Lactobacillales | f__Leuconostocaceae | v_Leuconostocaceae_Unknown | 0 | 17 | 0.004447 | 0.447712 |
| p__Actinobacteria | c__Actinobacteria | o__Actinomycetales | f__Microbacteriaceae | v_Microbacterium | 0 | 17 | 0.004811 | 0.387632 |
| p__Firmicutes | c__Clostridia | o__Clostridiales | f__Veillonellaceae | v_Megasphaera | 0 | 17 | 0.021026 | 0.304575 |
| p__Proteobacteria | c__Alphaproteobacteria | o__Sphingomonadales | f__Sphingomonadaceae | v_Sphingomonas | 0 | 17 | 0.021026 | 0.304575 |
| p__Proteobacteria | c__Gammaproteobacteria | o__Xanthomonadales | f__Xanthomonadaceae | v_Stenotrophomonas | 0 | 17 | 0.025794 | 0.381636 |
| p__Firmicutes | c__Bacilli | o__Lactobacillales | f__Streptococcaceae | v_Lactococcus | 0 | 17 | 0.037349 | 0.408669 |
| p__Firmicutes | c__Bacilli | o__Bacillales | f__Bacillaceae | g_Bacillaceae_Unknown | 0 | 18 | 0.006603 | 0.357143 |
| p__Actinobacteria | c__Actinobacteria | o__Actinomycetales | f__Microbacteriaceae | v_Microbacteriaceae_Unknown | 0 | 18 | 0.006603 | 0.357143 |
| p__Firmicutes | c__Clostridia | o__Clostridiales | f__[Tissierellaceae] | v_Sporanaerobacter | 0 | 18 | 0.006603 | 0.357143 |
| p__Bacteroidetes | c__Bacteroidia | o__Bacteroidales | f__Porphyromonadaceae | v_Dysgonomonas | 0 | 19 | 0.008613 | 0.337321 |
| p__Firmicutes | c__Bacilli | o__Bacillales | f__Thermoactinomycetaceae | v_Thermoactinomycetaceae_Other | 0 | 19 | 0.008613 | 0.337321 |
| p__Firmicutes | c__Clostridia | o__Clostridiales | f__Lachnospiraceae | g_Lachnospiraceae_Unknown | 1 | 5 | 1.83E-04 | 0.68 |
| p__Bacteroidetes | c__[Saprospirae] | o__[Saprospirales] | f__Chitinophagaceae | v_Chitinophagaceae_Unknown | 1 | 5 | 0.003353 | 0.511111 |
| p__Firmicutes | c__Clostridia | o__Clostridiales | f__Peptostreptococcaceae | g_Peptostreptococcaceae_Other | 1 | 5 | 0.014255 | 0.253846 |
| p__Firmicutes | c__Clostridia | o__Clostridiales | f__[Tissierellaceae] | g_[Tissierellaceae]_Unknown | 1 | 8 | 0.008265 | 0.409091 |
| p__Firmicutes | c__Clostridia | o__Clostridiales | Clostridiales_Unknown_f | g_Clostridiales_Unknown | 1 | 8 | 0.008265 | 0.409091 |
| p__Firmicutes | c__Bacilli | o__Bacillales | f__Paenibacillaceae | g_Brevibacillus | 1 | 8 | 0.027841 | 0.351415 |
| p__Bacteroidetes | c__[Saprospirae] | o__[Saprospirales] | f__Chitinophagaceae | g_Chitinophagaceae_Unknown | 1 | 9 | 0.002468 | 0.440613 |
| p__Firmicutes | c__Bacilli | o__Bacillales | f__Paenibacillaceae | v_Paenibacillus | 1 | 9 | 0.027846 | 0.37037 |
| p__Proteobacteria | c__Gammaproteobacteria | o__Pseudomonadales | f__Moraxellaceae | g_Enhydrobacter | 1 | 10 | 0.00536 | 0.416667 |
| p__Actinobacteria | c__Actinobacteria | o__Actinomycetales | f__Propionibacteriaceae | g_Propionibacterium | 1 | 10 | 0.00536 | 0.416667 |
| p__Firmicutes | c__Clostridia | o__Clostridiales | f__Clostridiaceae | g_Clostridiaceae_Unknown | 1 | 11 | 0.045812 | 0.316116 |
| p__Firmicutes | c__Bacilli | o__Bacillales | f__Bacillaceae | v_Bacillus | 1 | 12 | 0.0052 | 0.268519 |
| p__Bacteroidetes | c__Bacteroidia | o__Bacteroidales | f__Porphyromonadaceae | g_Dysgonomonas | 1 | 13 | 0.004936 | 0.273954 |
| p__Bacteroidetes | c__Flavobacteriia | o__Flavobacteriales | f__[Weeksellaceae] | g_[Weeksellaceae]_Unknown | 1 | 13 | 0.013795 | 0.389423 |
| p__Cyanobacteria | c__Chloroplast | o__Streptophyta | Streptophyta_Unknown_f | g_Streptophyta_Unknown | 1 | 13 | 0.013795 | 0.389423 |
| p__Proteobacteria | c__Alphaproteobacteria | o__Rhizobiales | f__Rhizobiaceae | g_Agrobacterium | 1 | 14 | 0.017058 | 0.448328 |
| p__Proteobacteria | c__Gammaproteobacteria | o__Xanthomonadales | f__Xanthomonadaceae | g_Xanthomonadaceae_Other | 1 | 16 | 0.002421 | 0.522837 |
| p__Bacteroidetes | c__Bacteroidia | o__Bacteroidales | f__S24-7 | g_S24-7_Unknown | 1 | 16 | 0.010147 | 0.35636 |
| p__Firmicutes | c__Bacilli | o__Lactobacillales | f__Enterococcaceae | g_Vagococcus | 1 | 17 | 0.005573 | 0.501665 |
| p__Bacteroidetes | c__Flavobacteriia | o__Flavobacteriales | f__[Weeksellaceae] | g_Chryseobacterium | 1 | 17 | 0.011262 | 0.366357 |
| p__Proteobacteria | c__Betaproteobacteria | o__Neisseriales | f__Neisseriaceae | g_Neisseriaceae_Unknown | 1 | 17 | 0.011262 | 0.366357 |
| p__Proteobacteria | c__Gammaproteobacteria | o__Enterobacteriales | f__Enterobacteriaceae | v_Enterobacteriaceae_Unknown | 1 | 17 | 0.023893 | 0.270588 |
| p__Proteobacteria | c__Gammaproteobacteria | o__Enterobacteriales | f__Enterobacteriaceae | g_Providencia | 1 | 18 | 0.035589 | 0.273273 |
| p__Firmicutes | c__Bacilli | o__Bacillales | f__Bacillaceae | g_Bacillus | 1 | 18 | 0.037014 | 0.271021 |
| p__Firmicutes | c__Clostridia | o__Clostridiales | f__[Tissierellaceae] | g_Sporanaerobacter | 1 | 20 | 0.006546 | 0.483333 |
| p__Proteobacteria | c__Betaproteobacteria | o__Burkholderiales | f__Alcaligenaceae | g_Achromobacter | 1 | 21 | 0.006878 | 0.488536 |
| p__Proteobacteria | c__Betaproteobacteria | o__Burkholderiales | f__Comamonadaceae | g_Comamonas | 1 | 22 | 0.007191 | 0.491582 |
| p__Proteobacteria | c__Gammaproteobacteria | o__Alteromonadales | f__Shewanellaceae | g_Shewanella | 1 | 22 | 0.007191 | 0.491582 |
| p__Proteobacteria | c__Betaproteobacteria | o__Rhodocyclales | f__Rhodocyclaceae | g_Thauera | 1 | 22 | 0.007191 | 0.491582 |
| p__Firmicutes | c__Bacilli | o__Bacillales | f__Thermoactinomycetaceae | v_Thermoactinomycetaceae_Unknown | 1 | 22 | 0.012167 | 0.481818 |
| p__Firmicutes | c__Bacilli | o__Bacillales | f__Paenibacillaceae | g_Paenibacillus | 1 | 22 | 0.02638 | 0.371843 |
| p__Proteobacteria | c__Alphaproteobacteria | o__BD7-3 | BD7-3_Unknown_f | g_BD7-3_Unknown | 1 | 22 | 0.042152 | 0.324198 |
| p__Firmicutes | c__Bacilli | o__Lactobacillales | f__Lactobacillaceae | g_Lactobacillus | 1 | 22 | 0.042152 | 0.324198 |
| p__Firmicutes | c__Bacilli | o__Lactobacillales | f__Enterococcaceae | g_Enterococcus | 1 | 23 | 0.004463 | 0.495311 |
|  |  |  |  | H | 1 | 23 | 0.01352 | 0.431835 |
| p__Firmicutes | c__Bacilli | o__Lactobacillales | f__Lactobacillaceae | g_Pediococcus | 1 | 23 | 0.035801 | 0.350242 |
| p__Firmicutes | c__Bacilli | o__Lactobacillales | Lactobacillales_Other_f | v_Lactobacillales_Other | 1 | 24 | 0.019588 | 0.40793 |
| p__Proteobacteria | c__Deltaproteobacteria | o__Desulfuromonadales | f__Geobacteraceae | g_Geobacter | 1 | 25 | 0.00564 | 0.494902 |
| p__Firmicutes | c__Bacilli | o__Lactobacillales | f__Lactobacillaceae | v_Lactobacillus | 1 | 25 | 0.00564 | 0.494902 |
| p__Firmicutes | c__Bacilli | o__Bacillales | f__Planococcaceae | v_Planococcaceae_Other | 1 | 25 | 0.013348 | 0.425 |
| p__Firmicutes | c__Bacilli | o__Lactobacillales | f__Lactobacillaceae | g_Lactobacillaceae_Other | 1 | 25 | 0.018461 | 0.432 |
| p__Firmicutes | c__Bacilli | o__Lactobacillales | f__Leuconostocaceae | g_Leuconostocaceae_Other | 1 | 26 | 0.014039 | 0.426683 |
| p__Firmicutes | c__Bacilli | o__Lactobacillales | Lactobacillales_Unknown_f | v_Lactobacillales_Unknown | 1 | 26 | 0.014039 | 0.426683 |
| p__Proteobacteria | c__Epsilonproteobacteria | o__Campylobacterales | f__Campylobacteraceae | g_Arcobacter | 1 | 26 | 0.017476 | 0.415264 |
| p__Firmicutes | c__Bacilli | o__Bacillales | f__Planococcaceae | g_Ureibacillus | 1 | 30 | 0.003811 | 0.46954 |
| p__Actinobacteria | c__Actinobacteria | o__Actinomycetales | f__Microbacteriaceae | g_Leucobacter | 1 | 30 | 0.003912 | 0.467816 |
| p__Proteobacteria | c__Gammaproteobacteria | o__Pseudomonadales | f__Moraxellaceae | g_Acinetobacter | 1 | 30 | 0.004136 | 0.468966 |
| p__Actinobacteria | c__Actinobacteria | o__Actinomycetales | f__Propionibacteriaceae | g_Propionibacteriaceae_Unknown | 1 | 31 | 0.004372 | 0.466073 |
| p__Firmicutes | c__Bacilli | o__Lactobacillales | f__Streptococcaceae | g_Streptococcus | 1 | 31 | 0.004372 | 0.466073 |
| p__Firmicutes | c__Bacilli | o__Bacillales | Bacillales_Unknown_f | v_Bacillales_Unknown | 1 | 31 | 0.004372 | 0.466073 |
| p__Proteobacteria | c__Alphaproteobacteria | o__Sphingomonadales | f__Sphingomonadaceae | g_Novosphingobium | 1 | 31 | 0.006872 | 0.448925 |
|  |  |  |  | C | 1 | 31 | 0.00737 | 0.483284 |
| p__Bacteroidetes | c__Bacteroidia | o__Bacteroidales | f__Rikenellaceae | g_Rikenellaceae_Unknown | 1 | 32 | 0.008387 | 0.443229 |
| p__Firmicutes | c__Clostridia | o__Clostridiales | f__[Tissierellaceae] | g_Tepidimicrobium | 1 | 32 | 0.008387 | 0.443229 |
| p__Proteobacteria | c__Gammaproteobacteria | o__Enterobacteriales | f__Enterobacteriaceae | g_Enterobacteriaceae_Unknown | 1 | 33 | 0.008094 | 0.480992 |
| p__Proteobacteria | c__Gammaproteobacteria | o__Pseudomonadales | f__Pseudomonadaceae | g_Pseudomonas | 1 | 33 | 0.008094 | 0.480992 |
|  |  |  |  | S | 1 | 33 | 0.008094 | 0.480992 |
| p__Actinobacteria | c__Actinobacteria | o__Actinomycetales | f__Corynebacteriaceae | g_Corynebacterium | 1 | 33 | 0.008858 | 0.439394 |
| p__Proteobacteria | c__Alphaproteobacteria | o__Sphingomonadales | f__Sphingomonadaceae | g_Sphingomonas | 1 | 33 | 0.008858 | 0.439394 |
| p__Firmicutes | c__Bacilli | o__Bacillales | f__Staphylococcaceae | g_Staphylococcus | 1 | 33 | 0.008858 | 0.439394 |
| p__Proteobacteria | c__Epsilonproteobacteria | o__Campylobacterales | f__Campylobacteraceae | v_Arcobacter | 2 | 1 | 0 | 0 |
| p__Proteobacteria | c__Gammaproteobacteria | o__Xanthomonadales | f__Xanthomonadaceae | v_Lysobacter | 2 | 2 | 0.00109 | 0.5 |
| p__Actinobacteria | c__Actinobacteria | o__Actinomycetales | f__Pseudonocardiaceae | g_Saccharomonospora | 2 | 2 | 0.002796 | 0.5 |
| p__Proteobacteria | c__Epsilonproteobacteria | o__Campylobacterales | f__Campylobacteraceae | v_Campylobacter | 2 | 2 | 0.005598 | 0.5 |
| p__Actinobacteria | c__Actinobacteria | o__Actinomycetales | f__Actinomycetaceae | v_Actinomyces | 2 | 3 | 0 | 0.708333 |
| p__Proteobacteria | c__Gammaproteobacteria | o__Enterobacteriales | f__Enterobacteriaceae | v_Providencia | 2 | 3 | 0 | 0.555556 |
| p__Firmicutes | c__Bacilli | o__Lactobacillales | f__Enterococcaceae | v_Vagococcus | 2 | 3 | 0 | 0.555556 |
| p__Proteobacteria | c__Betaproteobacteria | o__Burkholderiales | f__Comamonadaceae | v_Comamonas | 2 | 4 | 0 | 0.434211 |
| p__Firmicutes | c__Bacilli | o__Bacillales | f__Staphylococcaceae | v_Staphylococcus | 2 | 4 | 0 | 0.434211 |
| p__Proteobacteria | c__Gammaproteobacteria | o__Xanthomonadales | f__Xanthomonadaceae | v_Xanthomonadaceae_Other | 2 | 4 | 0 | 0.434211 |
| p__Firmicutes | c__Bacilli | o__Bacillales | f__Bacillaceae | v_Natronobacillus | 2 | 4 | 0.003482 | 0.375 |
| p__Firmicutes | c__Bacilli | o__Lactobacillales | f__Enterococcaceae | v_Enterococcaceae_Other | 2 | 4 | 0.005442 | 0.53125 |
| p__Proteobacteria | c__Gammaproteobacteria | o__Pseudomonadales | f__Moraxellaceae | v_Psychrobacter | 2 | 4 | 0.008726 | 0.359375 |
| p__Firmicutes | c__Bacilli | o__Lactobacillales | f__Enterococcaceae | v_Enterococcus | 2 | 4 | 0.026767 | 0.3125 |
| p__Firmicutes | c__Clostridia | o__Clostridiales | f__Lachnospiraceae | g_Coprococcus | 2 | 5 | 0 | 0.644444 |
| p__Firmicutes | c__Bacilli | o__Lactobacillales | f__Lactobacillaceae | g_Lactobacillaceae_Unknown | 2 | 5 | 0 | 0.644444 |
| p__Firmicutes | c__Bacilli | o__Bacillales | f__Planococcaceae | g_Planococcaceae_Unknown | 2 | 5 | 0 | 0.568421 |
| p__Firmicutes | c__Bacilli | o__Bacillales | f__Planococcaceae | v_Planococcaceae_Unknown | 2 | 5 | 0 | 0.568421 |
| p__Actinobacteria | c__Actinobacteria | o__Actinomycetales | f__Propionibacteriaceae | v_Propionibacteriaceae_Unknown | 2 | 5 | 0 | 0.644444 |
| p__Firmicutes | c__Bacilli | o__Bacillales | Bacillales_Other_f | v_Bacillales_Other | 2 | 5 | 0.00186 | 0.426667 |
|  |  |  |  | N | 2 | 5 | 0.003533 | 0.29375 |
| p__Firmicutes | c__Clostridia | o__Clostridiales | f__Veillonellaceae | g_Megasphaera | 2 | 5 | 0.006863 | 0.3625 |
| p__Firmicutes | c__Clostridia | o__Clostridiales | f__Lachnospiraceae | v_Coprococcus | 2 | 5 | 0.016128 | 0.368 |
| p__Actinobacteria | c__Actinobacteria | o__Actinomycetales | f__Pseudonocardiaceae | v_Saccharomonospora | 2 | 5 | 0.024847 | 0.238095 |
| p__Proteobacteria | c__Gammaproteobacteria | o__Enterobacteriales | f__Enterobacteriaceae | v_Enterobacteriaceae_Other | 2 | 5 | 0.028588 | 0.2625 |
| p__Proteobacteria | c__Gammaproteobacteria | o__Oceanospirillales | f__Alcanivoracaceae | g_Alcanivorax | 2 | 6 | 5.74E-04 | 0.569444 |
| p__Actinobacteria | c__Actinobacteria | o__Actinomycetales | f__Nocardioidaceae | v_Nocardioidaceae_Unknown | 2 | 6 | 5.74E-04 | 0.569444 |
| p__Proteobacteria | c__Gammaproteobacteria | o__Enterobacteriales | f__Enterobacteriaceae | v_Proteus | 2 | 6 | 8.14E-04 | 0.372549 |
| p__Actinobacteria | c__Actinobacteria | o__Actinomycetales | f__Brevibacteriaceae | g_Brevibacterium | 2 | 6 | 0.001435 | 0.425926 |
| p__Firmicutes | c__Bacilli | o__Lactobacillales | f__Leuconostocaceae | g_Leuconostocaceae_Unknown | 2 | 6 | 0.004333 | 0.404762 |
| p__Firmicutes | c__Bacilli | o__Bacillales | f__Planococcaceae | v_Sporosarcina | 2 | 6 | 0.008173 | 0.316667 |
| p__Firmicutes | c__Bacilli | o__Bacillales | f__Planococcaceae | g_Planococcaceae_Other | 2 | 6 | 0.008618 | 0.318182 |
| p__Bacteroidetes | c__Bacteroidia | o__Bacteroidales | f__Bacteroidaceae | g_Bacteroides | 2 | 6 | 0.012244 | 0.263441 |
| p__Firmicutes | c__Bacilli | o__Bacillales | f__Bacillaceae | v_Bacillaceae_Other | 2 | 6 | 0.016016 | 0.42029 |
| p__Firmicutes | c__Bacilli | o__Bacillales | Bacillales_Other_f | g_Bacillales_Other | 2 | 6 | 0.016041 | 0.263889 |
| p__Firmicutes | c__Clostridia | o__Clostridiales | f__Ruminococcaceae | g_Ruminococcaceae_Unknown | 2 | 6 | 0.016335 | 0.315789 |
| p__Firmicutes | c__Bacilli | o__Bacillales | f__Planococcaceae | v_Solibacillus | 2 | 6 | 0.016973 | 0.423077 |
| p__Firmicutes | c__Bacilli | o__Haloplasmatales | f__Haloplasmataceae | g_Haloplasmataceae_Unknown | 2 | 6 | 0.017282 | 0.404762 |
| p__Firmicutes | c__Clostridia | o__Clostridiales | Clostridiales_Other_f | g_Clostridiales_Other | 2 | 6 | 0.0297 | 0.293103 |
| p__Firmicutes | c__Clostridia | o__Clostridiales | f__Lachnospiraceae | g_Epulopiscium | 2 | 6 | 0.035408 | 0.22 |
| p__Firmicutes | c__Clostridia | o__Clostridiales | f__Clostridiaceae | g_Clostridium | 2 | 7 | 0.001731 | 0.412698 |
| Other | Other | Other | Other | g_Other | 2 | 7 | 0.003801 | 0.403361 |
| p__Firmicutes | c__Bacilli | o__Bacillales | f__Bacillaceae | v_Bacillaceae_Unknown | 2 | 7 | 0.008293 | 0.361111 |
| p__Firmicutes | c__Clostridia | o__Clostridiales | f__[Tissierellaceae] | g_Tissierella_Soehngenia | 2 | 7 | 0.012721 | 0.353383 |
| p__Actinobacteria | c__Actinobacteria | o__Bifidobacteriales | f__Bifidobacteriaceae | v_Bifidobacterium | 2 | 7 | 0.019194 | 0.366071 |
| p__Firmicutes | c__Clostridia | o__Clostridiales | f__[Tissierellaceae] | g_Anaerococcus | 2 | 7 | 0.02259 | 0.244048 |
| p__Firmicutes | c__Bacilli | o__Bacillales | f__Thermoactinomycetaceae | g_Thermoactinomycetaceae_Other | 2 | 7 | 0.023039 | 0.21645 |
| p__Tenericutes | c__Mollicutes | o__RsaHF231 | RsaHF231_Unknown_f | g_RsaHF232_Unknown | 2 | 7 | 0.029714 | 0.199134 |
| p__Actinobacteria | c__Actinobacteria | o__Actinomycetales | f__Microbacteriaceae | v_Leucobacter | 2 | 8 | 0.001262 | 0.410714 |
| p__Firmicutes | c__Bacilli | o__Lactobacillales | f__Streptococcaceae | g_Lactococcus | 2 | 8 | 0.002418 | 0.371795 |
| p__Firmicutes | c__Bacilli | o__Lactobacillales | f__Leuconostocaceae | g_Leuconostoc | 2 | 8 | 0.002418 | 0.371795 |
| p__Firmicutes | c__Clostridia | o__Clostridiales | f__Caldicoprobacteraceae | v_Caldicoprobacter | 2 | 8 | 0.007682 | 0.28125 |
| p__Bacteroidetes | c__Flavobacteriia | o__Flavobacteriales | f__[Weeksellaceae] | v_[Weeksellaceae]_Unknown | 2 | 8 | 0.008165 | 0.271739 |
| p__Firmicutes | c__Clostridia | o__Clostridiales | f__Clostridiaceae | v_Clostridiaceae_Unknown | 2 | 8 | 0.010969 | 0.334459 |
| p__Actinobacteria | c__Actinobacteria | o__Actinomycetales | f__Microbacteriaceae | g_Microbacteriaceae_Unknown | 2 | 8 | 0.012249 | 0.237245 |
| p__Firmicutes | c__Bacilli | o__Lactobacillales | f__Enterococcaceae | g_Enterococcaceae_Other | 2 | 8 | 0.017024 | 0.2125 |
| p__Proteobacteria | c__Epsilonproteobacteria | o__Campylobacterales | f__Campylobacteraceae | g_Campylobacter | 2 | 8 | 0.021827 | 0.212766 |
| p__Firmicutes | c__Clostridia | o__Clostridiales | f__[Tissierellaceae] | g_[Tissierellaceae]_Other | 2 | 8 | 0.025753 | 0.326389 |
| p__Firmicutes | c__Bacilli | o__Bacillales | f__Bacillaceae | v_Anoxybacillus | 2 | 8 | 0.030004 | 0.255 |
| p__Tenericutes | c__Mollicutes | o__RsaHF231 | RsaHF231_Unknown_f | v_RsaHF232_Unknown | 2 | 9 | 0.006773 | 0.297158 |
| p__Firmicutes | c__Bacilli | o__Lactobacillales | Lactobacillales_Other_f | g_Lactobacillales_Other | 2 | 10 | 0.008336 | 0.245652 |
| p__Firmicutes | c__Bacilli | o__Bacillales | f__Bacillaceae | g_Oceanobacillus | 2 | 10 | 0.011854 | 0.316667 |
| p__Proteobacteria | c__Gammaproteobacteria | o__Alteromonadales | f__Shewanellaceae | v_Shewanella | 2 | 10 | 0.082888 | 0.265574 |
| p__Firmicutes | c__Clostridia | o__Clostridiales | f__Lachnospiraceae | v_Lachnospiraceae_Unknown | 2 | 11 | 0.010367 | 0.253119 |
| p__Firmicutes | c__Bacilli | o__Bacillales | f__Planococcaceae | v_Rummeliibacillus | 2 | 11 | 0.010936 | 0.266925 |
|  |  |  |  | P | 2 | 11 | 0.012292 | 0.334928 |
| p__Firmicutes | c__Bacilli | o__Lactobacillales | f__Lactobacillaceae | v_Lactobacillaceae_Other | 2 | 12 | 0.006443 | 0.231481 |
| p__Bacteroidetes | c__Bacteroidia | o__Bacteroidales | f__Rikenellaceae | v_Rikenellaceae_Unknown | 2 | 12 | 0.042453 | 0.193989 |
| p__Actinobacteria | c__Actinobacteria | o__Actinomycetales | f__Propionibacteriaceae | v_Propionibacterium | 2 | 12 | 0.051196 | 0.215278 |
| p__Firmicutes | c__Bacilli | o__Bacillales | f__Bacillaceae | g_Virgibacillus | 2 | 12 | 0.051394 | 0.223485 |
| p__Firmicutes | c__Clostridia | o__Clostridiales | f__Lachnospiraceae | v_Epulopiscium | 2 | 13 | 0.004053 | 0.321368 |
| p__Firmicutes | c__Clostridia | o__Clostridiales | f__Ruminococcaceae | v_Ruminococcaceae_Unknown | 2 | 13 | 0.004053 | 0.321368 |
| p__Firmicutes | c__Bacilli | o__Lactobacillales | Lactobacillales_Unknown_f | g_Lactobacillales_Unknown | 2 | 13 | 0.010886 | 0.204694 |
| p__Firmicutes | c__Clostridia | o__Clostridiales | f__Clostridiaceae | g_Alkaliphilus | 2 | 13 | 0.01327 | 0.22929 |
| p__Firmicutes | c__Bacilli | o__Bacillales | f__Bacillaceae | v_Virgibacillus | 2 | 13 | 0.014252 | 0.26454 |
| p__Bacteroidetes | c__Bacteroidia | o__Bacteroidales | f__S24-7 | v_S24-7_Unknown | 2 | 13 | 0.037386 | 0.225071 |
| p__Firmicutes | c__Clostridia | o__Clostridiales | f__Clostridiaceae | v_Clostridium | 2 | 14 | 0.003697 | 0.331502 |
| p__Firmicutes | c__Bacilli | o__Bacillales | f__Bacillaceae | v_Oceanobacillus | 2 | 14 | 0.010461 | 0.291793 |
| p__Firmicutes | c__Clostridia | o__Clostridiales | f__Peptostreptococcaceae | v_Peptostreptococcaceae_Other | 2 | 14 | 0.010461 | 0.291793 |
| p__Firmicutes | c__Bacilli | o__Bacillales | f__Bacillaceae | g_Anoxybacillus | 2 | 14 | 0.017096 | 0.282857 |
| p__Firmicutes | c__Clostridia | o__Clostridiales | Clostridiales_Other_f | v_Clostridiales_Other | 2 | 14 | 0.017096 | 0.282857 |
| p__Proteobacteria | c__Alphaproteobacteria | o__Rhodospirillales | f__Acetobacteraceae | g_Acetobacter | 2 | 14 | 0.018464 | 0.245847 |
| p__Firmicutes | c__Bacilli | o__Bacillales | f__Planococcaceae | g_Rummeliibacillus | 2 | 14 | 0.0208 | 0.225446 |
| p__Bacteroidetes | c__Flavobacteriia | o__Flavobacteriales | f__[Weeksellaceae] | v_Chryseobacterium | 2 | 14 | 0.030777 | 0.19697 |
| p__Actinobacteria | c__Actinobacteria | o__Actinomycetales | f__Corynebacteriaceae | v_Corynebacterium | 2 | 15 | 0.014437 | 0.242767 |
| p__Firmicutes | c__Bacilli | o__Bacillales | f__Paenibacillaceae | v_Brevibacillus | 2 | 15 | 0.021771 | 0.201093 |
| p__Firmicutes | c__Bacilli | o__Bacillales | f__Planococcaceae | g_Solibacillus | 2 | 16 | 0.010126 | 0.237981 |
| p__Proteobacteria | c__Betaproteobacteria | o__Burkholderiales | f__Alcaligenaceae | v_Achromobacter | 2 | 16 | 0.040443 | 0.258523 |
| p__Firmicutes | c__Bacilli | o__Lactobacillales | f__Lactobacillaceae | v_Lactobacillaceae_Unknown | 2 | 17 | 0.035293 | 0.197712 |
| p__Firmicutes | c__Clostridia | o__Clostridiales | Clostridiales_Unknown_f | v_Clostridiales_Unknown | 2 | 21 | 0.015913 | 0.238095 |
| p__Firmicutes | c__Clostridia | o__Clostridiales | f__[Tissierellaceae] | v_Anaerococcus | 2 | 22 | 0.027607 | 0.214176 |

**Table S8**. Important coefficients for co-occurrence network of NC.

| Phylum | Class | Order | Family | Name | Modularity Class | degree | Betweenness  Centrality | Topological  Coefficient |
| --- | --- | --- | --- | --- | --- | --- | --- | --- |
| p__Firmicutes | c__Clostridia | o__Clostridiales | f__Clostridiaceae | Clostridiaceae_Unknown | 0 | 2 | 0.006235 | 0.545455 |
| p__Firmicutes | c__Bacilli | o__Bacillales | f__Paenibacillaceae | Brevibacillus | 0 | 3 | 0.00173 | 0.611111 |
| p__Actinobacteria | c__Actinobacteria | o__Actinomycetales | f__Nocardioidaceae | Nocardioidaceae_Unknown | 0 | 3 | 0.020231 | 0.52381 |
| p__Proteobacteria | c__Gammaproteobacteria | o__Xanthomonadales | f__Xanthomonadaceae | Ignatzschineria | 0 | 3 | 0.020898 | 0.433333 |
| p__Bacteroidetes | c__Bacteroidia | o__Bacteroidales | f__S24-7 | S24-7_Unknown | 0 | 3 | 0.035065 | 0.393939 |
| p__Proteobacteria | c__Gammaproteobacteria | o__Xanthomonadales | f__Xanthomonadaceae | Lysobacter | 0 | 4 | 0.002902 | 0.46875 |
| p__Proteobacteria | c__Gammaproteobacteria | o__Oceanospirillales | f__Alcanivoracaceae | Alcanivorax | 0 | 4 | 0.01884 | 0.395833 |
| p__Actinobacteria | c__Actinobacteria | o__Actinomycetales | f__Propionibacteriaceae | Propionibacterium | 0 | 4 | 0.026937 | 0.291667 |
| p__Actinobacteria | c__Actinobacteria | o__Actinomycetales | f__Corynebacteriaceae | Corynebacterium | 0 | 5 | 0.003284 | 0.365217 |
| p__Proteobacteria | c__Alphaproteobacteria | o__Sphingomonadales | f__Sphingomonadaceae | Novosphingobium | 0 | 5 | 0.005447 | 0.428571 |
| p__Proteobacteria | c__Gammaproteobacteria | o__Enterobacteriales | f__Enterobacteriaceae | Proteus | 0 | 5 | 0.023343 | 0.326316 |
| p__Firmicutes | c__Clostridia | o__Clostridiales | f__[Tissierellaceae] | [Tissierellaceae]_Unknown | 0 | 7 | 0.017698 | 0.316964 |
| p__Proteobacteria | c__Gammaproteobacteria | o__Xanthomonadales | f__Xanthomonadaceae | Xanthomonadaceae_Other | 0 | 7 | 0.039895 | 0.357143 |
| p__Proteobacteria | c__Epsilonproteobacteria | o__Campylobacterales | f__Campylobacteraceae | Arcobacter | 0 | 7 | 0.041136 | 0.354286 |
| p__Proteobacteria | c__Alphaproteobacteria | o__Rhizobiales | f__Rhizobiaceae | Agrobacterium | 0 | 7 | 0.047794 | 0.357143 |
| p__Firmicutes | c__Bacilli | o__Bacillales | f__Thermoactinomycetaceae | Thermoactinomycetaceae_Other | 0 | 9 | 0.015241 | 0.374074 |
| p__Firmicutes | c__Bacilli | o__Bacillales | f__Planococcaceae | Sporosarcina | 0 | 9 | 0.015815 | 0.374074 |
| p__Firmicutes | c__Clostridia | o__Clostridiales | f__[Tissierellaceae] | [Tissierellaceae]_Other | 0 | 9 | 0.044697 | 0.292063 |
| p__Actinobacteria | c__Actinobacteria | o__Actinomycetales | f__Microbacteriaceae | Microbacterium | 0 | 9 | 0.076902 | 0.333333 |
| p__Firmicutes | c__Bacilli | o__Bacillales | Bacillales_Unknown_f | Bacillales_Unknown | 0 | 10 | 0.011288 | 0.436 |
| p__Firmicutes | c__Bacilli | o__Bacillales | f__Thermoactinomycetaceae | Thermoactinomycetaceae_Unknown | 0 | 11 | 0.036492 | 0.338843 |
| p__Actinobacteria | c__Actinobacteria | o__Actinomycetales | f__Brevibacteriaceae | Brevibacterium | 0 | 12 | 0.044248 | 0.348485 |
| p__Proteobacteria | c__Gammaproteobacteria | o__Pseudomonadales | f__Moraxellaceae | Psychrobacter | 0 | 12 | 0.048323 | 0.347917 |
| p__Proteobacteria | c__Betaproteobacteria | o__Burkholderiales | f__Alcaligenaceae | Achromobacter | 0 | 13 | 0.022818 | 0.432361 |
| p__Firmicutes | c__Bacilli | o__Lactobacillales | Lactobacillales_Unknown_f | Lactobacillales_Unknown | 0 | 14 | 0.033902 | 0.418367 |
| p__Firmicutes | c__Bacilli | o__Bacillales | f__Bacillaceae | Bacillaceae_Other | 0 | 14 | 0.069677 | 0.333929 |
| p__Firmicutes | c__Bacilli | o__Lactobacillales | f__Lactobacillaceae | Lactobacillus | 0 | 15 | 0.036913 | 0.331313 |
| p__Firmicutes | c__Bacilli | o__Lactobacillales | f__Lactobacillaceae | Lactobacillaceae_Unknown | 0 | 16 | 0.045501 | 0.335227 |
| p__Firmicutes | c__Bacilli | o__Lactobacillales | f__Lactobacillaceae | Lactobacillaceae_Other | 0 | 16 | 0.048266 | 0.327652 |
| p__Firmicutes | c__Bacilli | o__Lactobacillales | f__Aerococcaceae | Facklamia | 1 | 1 | 0 | 0 |
| p__Proteobacteria | c__Betaproteobacteria | o__Neisseriales | f__Neisseriaceae | Neisseriaceae_Unknown | 1 | 1 | 0 | 0 |
| p__Firmicutes | c__Clostridia | o__Clostridiales | f__[Tissierellaceae] | Tepidimicrobium | 1 | 1 | 0 | 0 |
| p__Firmicutes | c__Bacilli | o__Bacillales | f__Bacillaceae | Natronobacillus | 1 | 2 | 0.009846 | 0.5 |
| p__Firmicutes | c__Clostridia | o__Clostridiales | Clostridiales_Other_f | Clostridiales_Other | 1 | 2 | 0.020408 | 0.5 |
| p__Firmicutes | c__Bacilli | o__Lactobacillales | f__Lactobacillaceae | Pediococcus | 1 | 3 | 0.001536 | 0.491228 |
| p__Proteobacteria | c__Gammaproteobacteria | o__Alteromonadales | f__Shewanellaceae | Shewanella | 1 | 5 | 0.013436 | 0.327273 |
| p__Proteobacteria | c__Gammaproteobacteria | o__Pseudomonadales | f__Pseudomonadaceae | Pseudomonas | 1 | 5 | 0.020408 | 0.51 |
| p__Firmicutes | c__Bacilli | o__Lactobacillales | f__Streptococcaceae | Streptococcus | 1 | 7 | 0.014987 | 0.336406 |
| p__Cyanobacteria | c__Chloroplast | o__Streptophyta | Streptophyta_Unknown_f | Streptophyta_Unknown | 1 | 8 | 0.016861 | 0.33871 |
| p__Firmicutes | c__Bacilli | o__Bacillales | f__Planococcaceae | Rummeliibacillus | 1 | 8 | 0.032581 | 0.459821 |
| p__Firmicutes | c__Clostridia | o__Clostridiales | f__Caldicoprobacteraceae | Caldicoprobacter | 1 | 9 | 0.048901 | 0.333333 |
| p__Firmicutes | c__Clostridia | o__Clostridiales | f__Veillonellaceae | Megasphaera | 1 | 11 | 0.002585 | 0.503247 |
| p__Firmicutes | c__Bacilli | o__Bacillales | f__Bacillaceae | Virgibacillus | 1 | 11 | 0.00556 | 0.463636 |
| p__Actinobacteria | c__Actinobacteria | o__Actinomycetales | f__Microbacteriaceae | Microbacteriaceae_Unknown | 1 | 11 | 0.021287 | 0.43007 |
| p__Proteobacteria | c__Alphaproteobacteria | o__Rhodospirillales | f__Acetobacteraceae | Acetobacter | 1 | 12 | 0.003384 | 0.469444 |
| p__Firmicutes | c__Bacilli | o__Lactobacillales | f__Leuconostocaceae | Leuconostocaceae_Other | 1 | 13 | 0.003768 | 0.478022 |
| p__Bacteroidetes | c__Flavobacteriia | o__Flavobacteriales | f__[Weeksellaceae] | Chryseobacterium | 1 | 14 | 0.005729 | 0.447619 |
| p__Firmicutes | c__Bacilli | o__Lactobacillales | f__Streptococcaceae | Lactococcus | 1 | 14 | 0.005729 | 0.447619 |
| p__Firmicutes | c__Bacilli | o__Lactobacillales | f__Leuconostocaceae | Leuconostoc | 1 | 14 | 0.005729 | 0.447619 |
| p__Firmicutes | c__Bacilli | o__Bacillales | f__Planococcaceae | Solibacillus | 1 | 14 | 0.005729 | 0.447619 |
| p__Proteobacteria | c__Gammaproteobacteria | o__Xanthomonadales | f__Xanthomonadaceae | Stenotrophomonas | 1 | 14 | 0.005729 | 0.447619 |
| p__Proteobacteria | c__Alphaproteobacteria | o__Sphingomonadales | f__Sphingomonadaceae | Sphingomonas | 1 | 15 | 0.069945 | 0.347748 |
| p__Bacteroidetes | c__Bacteroidia | o__Bacteroidales | f__Prevotellaceae | Prevotella | 1 | 15 | 0.110495 | 0.343434 |
| p__Firmicutes | c__Bacilli | o__Lactobacillales | f__Leuconostocaceae | Leuconostocaceae_Unknown | 1 | 18 | 0.06489 | 0.31339 |
| p__Actinobacteria | c__Actinobacteria | o__Actinomycetales | f__Pseudonocardiaceae | Saccharomonospora | 1 | 18 | 0.06489 | 0.31339 |
| p__Firmicutes | c__Bacilli | o__Bacillales | f__Planococcaceae | Planococcaceae_Unknown | 2 | 1 | 0 | 0 |
| p__Bacteroidetes | c__Bacteroidia | o__Bacteroidales | Bacteroidales__Unknown_f | Bacteroidales_Unknown | 2 | 2 | 0 | 0.552632 |
| p__Firmicutes | c__Clostridia | o__Clostridiales | f__[Tissierellaceae] | Tissierella_Soehngenia | 2 | 2 | 0.003638 | 0.5 |
| p__Firmicutes | c__Clostridia | o__Clostridiales | f__Clostridiaceae | Alkaliphilus | 2 | 3 | 0 | 0.555556 |
| p__Proteobacteria | c__Gammaproteobacteria | o__Enterobacteriales | f__Enterobacteriaceae | Providencia | 2 | 3 | 5.06E-04 | 0.466667 |
| p__Firmicutes | c__Bacilli | o__Bacillales | f__Bacillaceae | Bacillus | 2 | 3 | 0.005271 | 0.444444 |
| p__Firmicutes | c__Bacilli | o__Bacillales | f__Bacillaceae | Bacillaceae_Unknown | 2 | 5 | 0.005784 | 0.388235 |
| p__Bacteroidetes | c__Bacteroidia | o__Bacteroidales | f__Bacteroidaceae | Bacteroides | 2 | 5 | 0.005784 | 0.388235 |
| p__Actinobacteria | c__Actinobacteria | o__Bifidobacteriales | f__Bifidobacteriaceae | Bifidobacterium | 2 | 5 | 0.019356 | 0.237037 |
| p__Proteobacteria | c__Epsilonproteobacteria | o__Campylobacterales | f__Campylobacteraceae | Campylobacter | 2 | 5 | 0.024536 | 0.365217 |
| p__Firmicutes | c__Bacilli | o__Bacillales | f__Paenibacillaceae | Paenibacillus | 2 | 6 | 0 | 0.617647 |
| p__Proteobacteria | c__Betaproteobacteria | o__Burkholderiales | f__Comamonadaceae | Comamonas | 2 | 6 | 0.061352 | 0.282828 |
| p__Firmicutes | c__Clostridia | o__Clostridiales | Clostridiales_Unknown_f | Clostridiales_Unknown | 2 | 6 | 0.065213 | 0.265432 |
| p__Firmicutes | c__Bacilli | o__Bacillales | Bacillales_Other_f | Bacillales_Other | 2 | 7 | 0.01153 | 0.457143 |
| p__Firmicutes | c__Clostridia | o__Clostridiales | f__Clostridiaceae | Clostridium | 2 | 7 | 0.023429 | 0.383117 |
| p__Firmicutes | c__Bacilli | o__Lactobacillales | f__Enterococcaceae | Enterococcaceae_Other | 2 | 7 | 0.024771 | 0.390977 |
| p__Bacteroidetes | c__Bacteroidia | o__Bacteroidales | f__Porphyromonadaceae | Dysgonomonas | 2 | 7 | 0.028528 | 0.371429 |
| p__Proteobacteria | c__Gammaproteobacteria | o__Pseudomonadales | f__Moraxellaceae | Acinetobacter | 2 | 8 | 0.020763 | 0.395 |
| p__Firmicutes | c__Bacilli | o__Bacillales | f__Bacillaceae | Oceanobacillus | 2 | 8 | 0.02596 | 0.339286 |
| p__Firmicutes | c__Clostridia | o__Clostridiales | f__Peptostreptococcaceae | Peptostreptococcaceae_Other | 2 | 8 | 0.026534 | 0.315 |
| p__Firmicutes | c__Bacilli | o__Bacillales | f__Bacillaceae | Anoxybacillus | 2 | 8 | 0.031938 | 0.414474 |
| p__Firmicutes | c__Clostridia | o__Clostridiales | f__[Tissierellaceae] | Anaerococcus | 2 | 8 | 0.036052 | 0.292857 |
| p__Actinobacteria | c__Actinobacteria | o__Actinomycetales | f__Microbacteriaceae | Leucobacter | 2 | 9 | 0.011312 | 0.434343 |
| p__Firmicutes | c__Bacilli | o__Lactobacillales | f__Enterococcaceae | Vagococcus | 2 | 9 | 0.011312 | 0.434343 |
| p__Actinobacteria | c__Actinobacteria | o__Actinomycetales | f__Actinomycetaceae | Actinomyces | 2 | 9 | 0.011864 | 0.361111 |
| p__Bacteroidetes | c__Flavobacteriia | o__Flavobacteriales | f__[Weeksellaceae] | [Weeksellaceae]_Unknown | 2 | 9 | 0.074812 | 0.307692 |
| p__Firmicutes | c__Bacilli | o__Lactobacillales | Lactobacillales_Other_f | Lactobacillales_Other | 2 | 10 | 0.029507 | 0.361538 |
| p__Firmicutes | c__Bacilli | o__Bacillales | f__Planococcaceae | Planococcaceae_Other | 2 | 10 | 0.029507 | 0.361538 |
| p__Tenericutes | c__Mollicutes | o__RsaHF231 | RsaHF231_Unknown_f | RsaHF232_Unknown | 2 | 10 | 0.032874 | 0.288235 |
| p__Firmicutes | c__Bacilli | o__Bacillales | f__Planococcaceae | Ureibacillus | 2 | 10 | 0.046408 | 0.236667 |
| p__Firmicutes | c__Bacilli | o__Haloplasmatales | f__Haloplasmataceae | Haloplasmataceae_Unknown | 2 | 11 | 0.017699 | 0.318182 |
| p__Firmicutes | c__Clostridia | o__Clostridiales | f__Lachnospiraceae | Coprococcus | 2 | 11 | 0.030513 | 0.331169 |
| p__Firmicutes | c__Clostridia | o__Clostridiales | f__Lachnospiraceae | Epulopiscium | 2 | 11 | 0.030873 | 0.28877 |
| p__Firmicutes | c__Clostridia | o__Clostridiales | f__Lachnospiraceae | Lachnospiraceae_Unknown | 2 | 11 | 0.036189 | 0.309091 |
| p__Firmicutes | c__Bacilli | o__Lactobacillales | f__Enterococcaceae | Enterococcus | 2 | 11 | 0.049803 | 0.316614 |
| p__Firmicutes | c__Clostridia | o__Clostridiales | f__[Tissierellaceae] | Sporanaerobacter | 2 | 11 | 0.056373 | 0.284091 |
| p__Proteobacteria | c__Gammaproteobacteria | o__Enterobacteriales | f__Enterobacteriaceae | Enterobacteriaceae_Unknown | 2 | 12 | 0.027515 | 0.286765 |
| p__Proteobacteria | c__Gammaproteobacteria | o__Enterobacteriales | f__Enterobacteriaceae | Enterobacteriaceae_Other | 2 | 13 | 0.040189 | 0.28733 |
| p__Firmicutes | c__Clostridia | o__Clostridiales | f__Ruminococcaceae | Ruminococcaceae_Unknown | 2 | 13 | 0.054213 | 0.291855 |
| p__Actinobacteria | c__Actinobacteria | o__Actinomycetales | f__Propionibacteriaceae | Propionibacteriaceae_Unknown | 3 | 1 | 0 | 0 |
| p__Firmicutes | c__Bacilli | o__Bacillales | f__Staphylococcaceae | Staphylococcus | 3 | 1 | 0 | 0 |

**Table S9** The numbers and [properties](file:///D:\Program%20Files%20(x86)\Youdao\Dict\7.2.0.0703\resultui\dict\?keyword=property) of the nodes and edges of the co-occurrence networks

| Correlation | Total number | Properties | Number | Notes |
| --- | --- | --- | --- | --- |
| vermicomposte bacteria-vermicomposte bacteria | 339 | + | 265 |  |
|  |  | - | 74 |  |
| gut bacteria -vermicomposte bacteria | 384 | + | 161 |  |
|  |  | - | 223 |  |
| gut bacteria - gut bacteria | 481 | + | 388 |  |
|  |  | - | 93 |  |
| vermicomposte bacteria -chemical elements | 29 | + | 26 |  |
|  |  | - | 3 | *v_Corynebacterium; v_Bacillus; v_Anaerococcus* |
| gut bacteria-chemical elements | 68 | + | 60 |  |
|  |  | - | 8 | *g_Ureibacillus; g_Sporosarcina; g_Bacillus; g_Sporanaerobacter* |
| chemical elements-chemical elements | 3 | + | 3 |  |
|  |  | - | 0 |  |


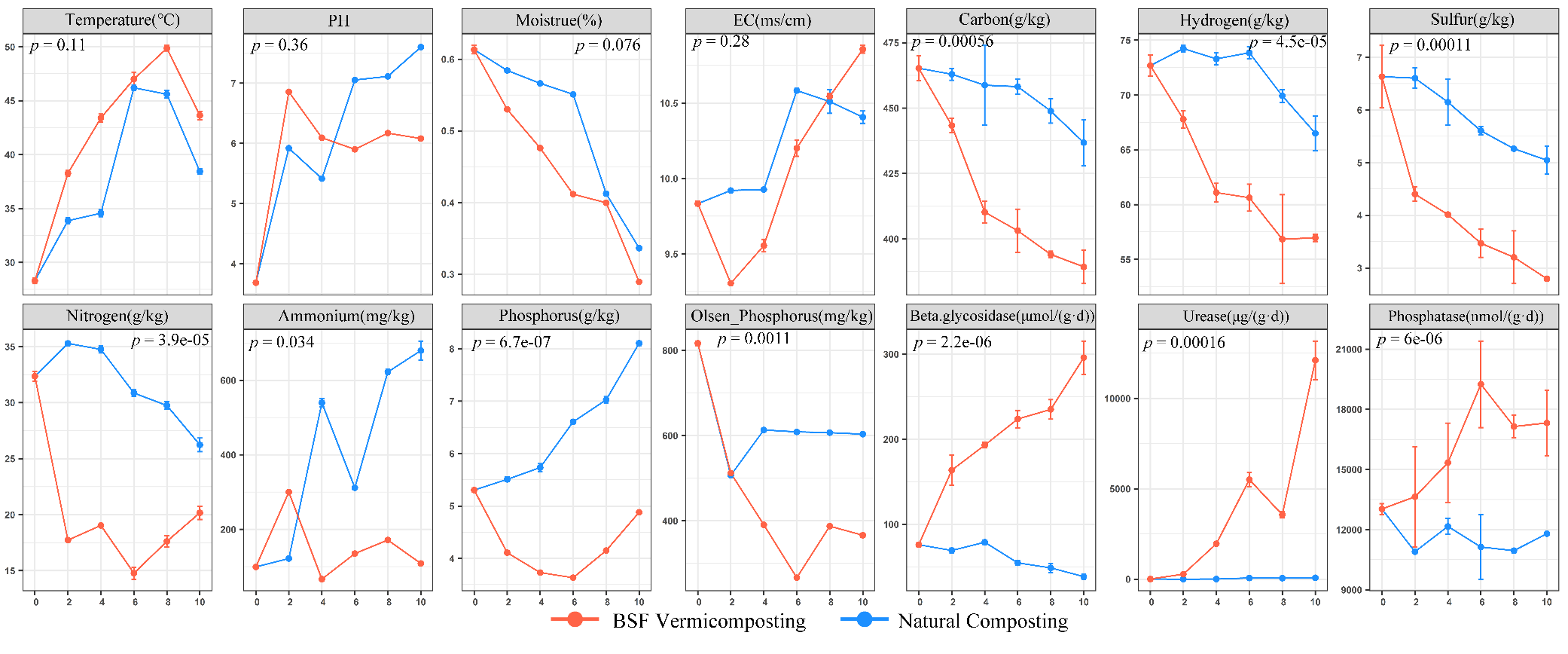


**Fig.S1** The dynamic change of physico-chemical parameters during BSF vermicomposting (BC, Orangered in color) and natural composting (NC, Blue in color). X-axis indicates the day of sampling. *p* value represents the extent of the overall difference. Error bars represent SD of triplicate.


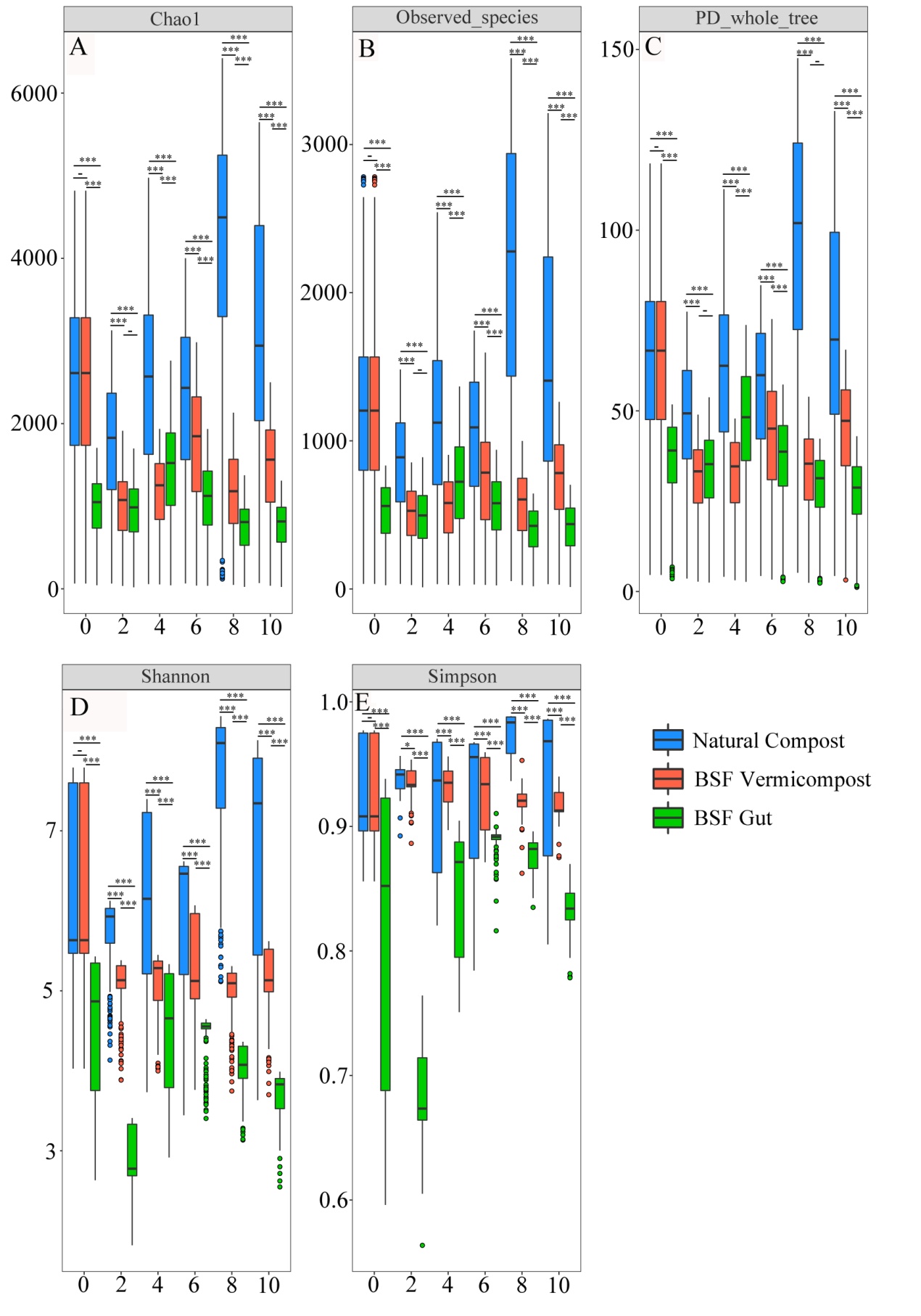


**Fig.S2** Alpha-diversity measurement among studied samples. Five calculated indices include A) Chao1, B) Observed_species, C) PD_whole_tree, D) Shannon, and E) Simpson for samples collected under natural composting (Blue in color), under vermicomposting processes using BSF larvae (Orange red in color), and larval gut samples (Green in color). X-axis indicates the day of sampling. In the box plots, the symbols indicate the following: box, 25th to 75th percentile; horizontal line, mean values; whiskers, 10th and 90th percentile. The circles above or below the box plots indicate Outlier. Each box is calculated from three samples using ggplot2. Statistical differences were assessed using the least significant difference test in the R package Agricolae. **–** *p* >0.05; * *p* < 0.05; ** *p* < 0.01; *** *p* < 0.001.


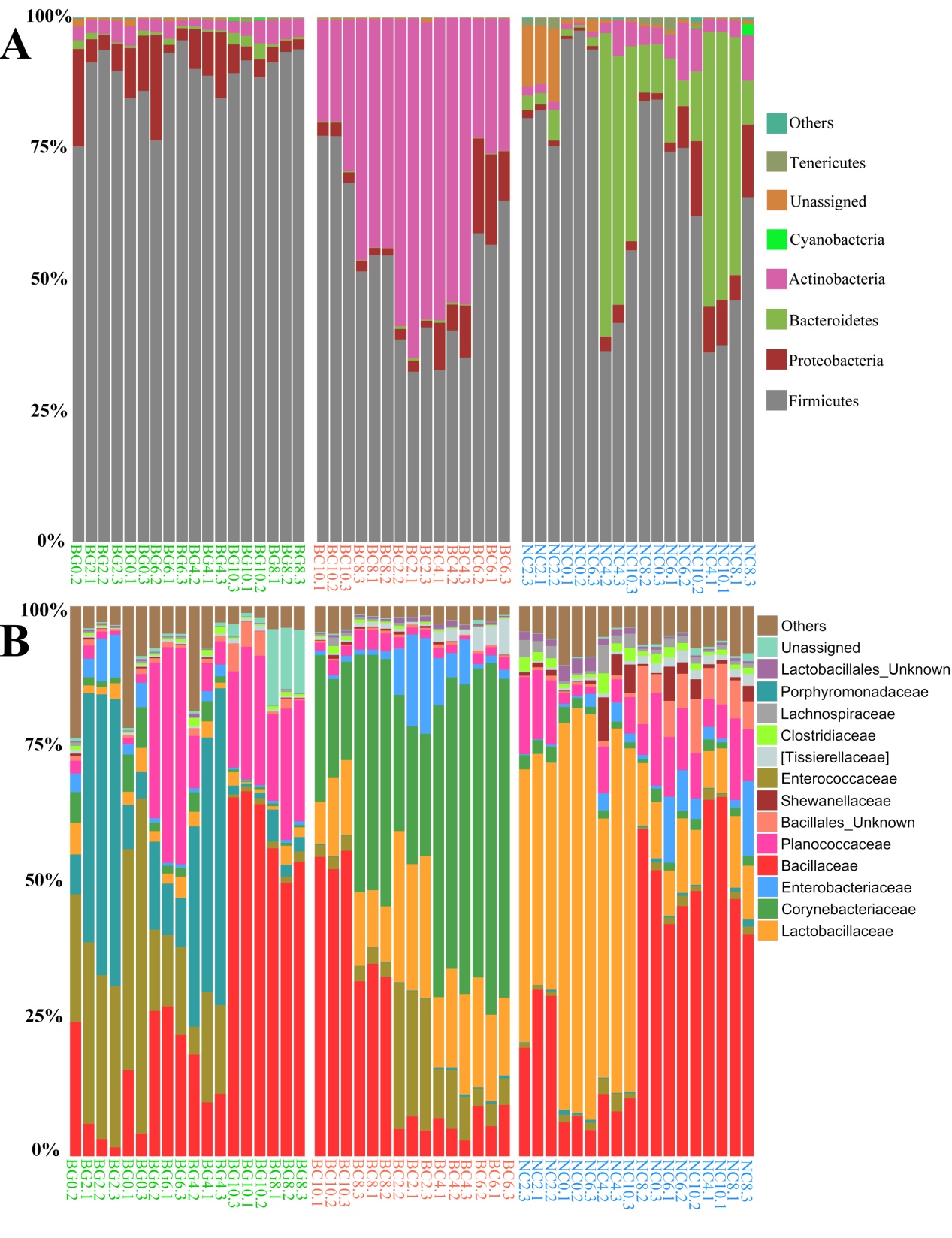


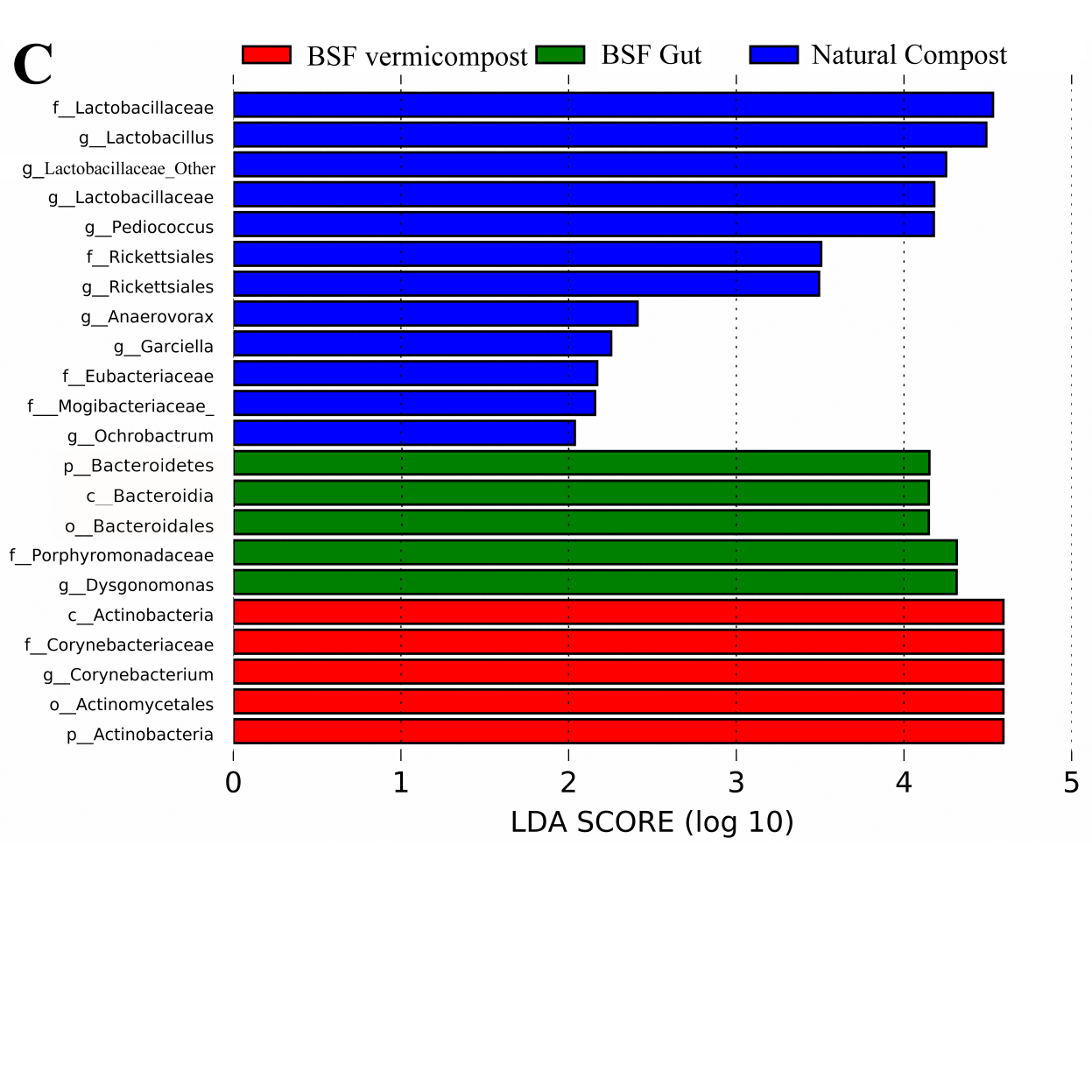


**Fig.S3** Relative abundance (%) of taxa at the level (A) phylum and family level (B), The samples are arranged according to the clustering tree. Taxa with <1% of reads were combined together as ‘Others’; while ‘Unassigned’ represents unclassified taxa. (C) LEfSe analyses among BC, BG and NC. NC enriched bacteria in blue; BG enriched bacteria in green; BC enriched bacteria in red. Only discriminant analysis score threshold >2 are shown.


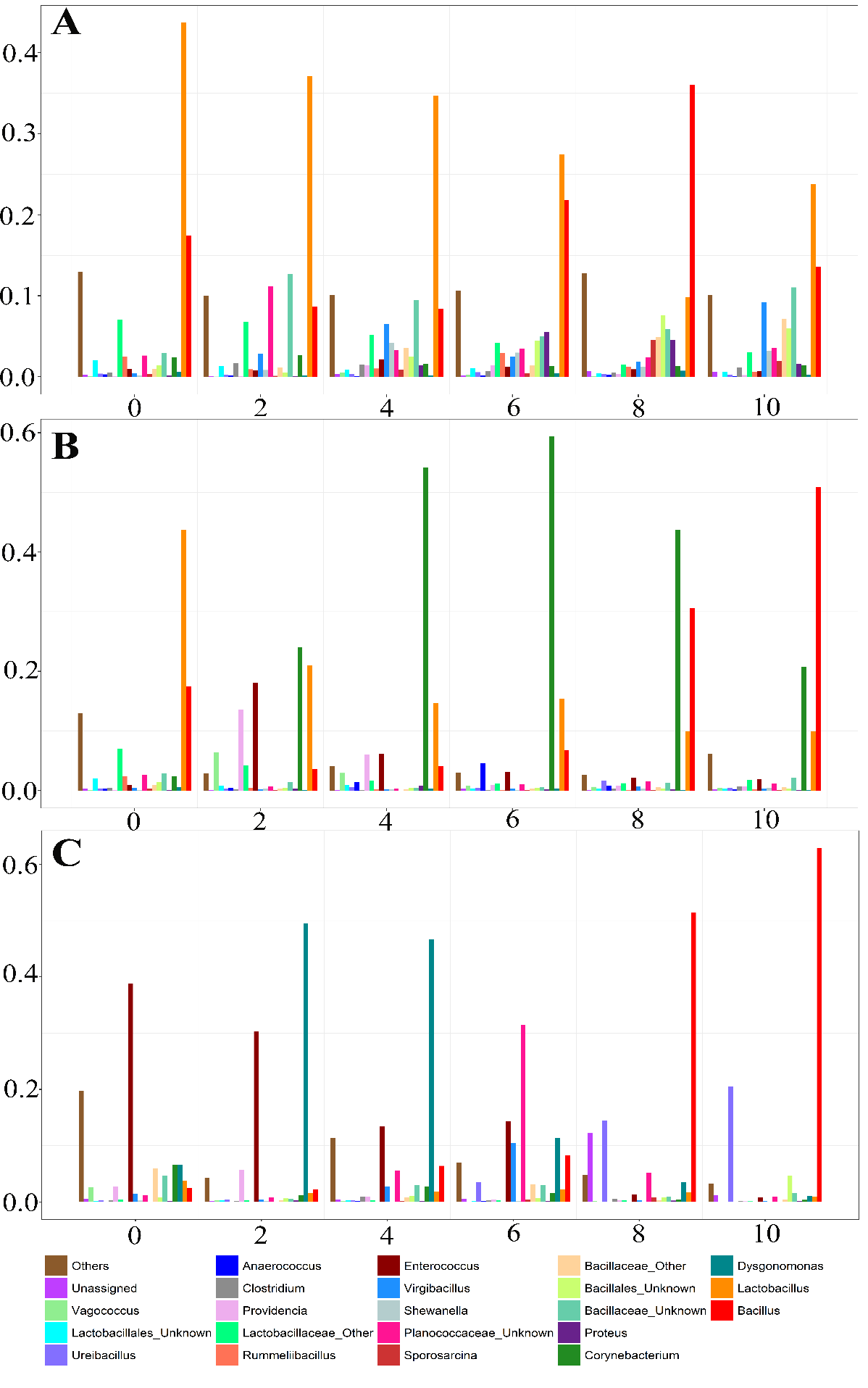


**Fig.S4** the dynamic change of relative abundance (%) of taxa at genus level in NC (A), BC (B) and BG(C), Taxa with <1% of reads were combined together as ‘Others’; while ‘Unassigned’ represents unclassified taxa at the genus level.

_
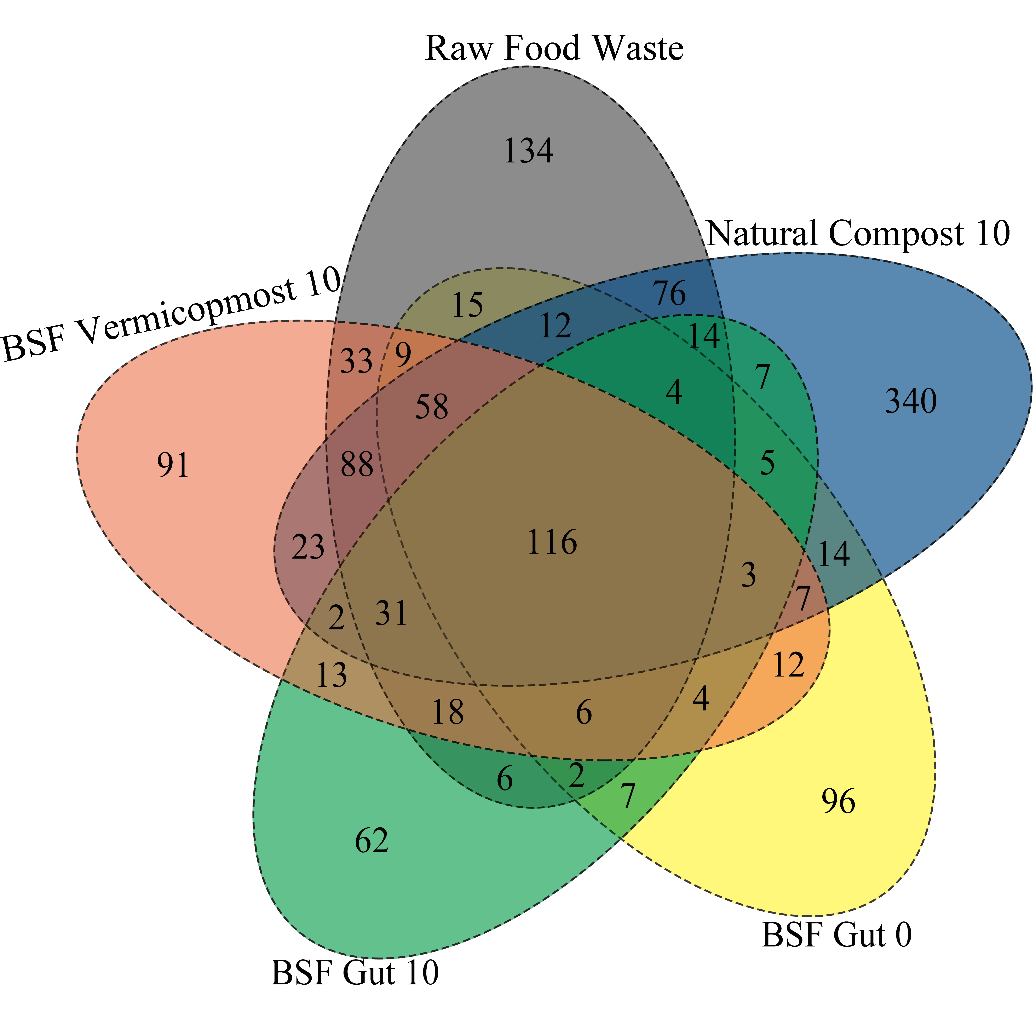
_

**Fig.S5** Exclusive and shared OTUs (non-singleton OTUs, based on 97% reads similarity) among raw FW, larvae gut (BG0 and BG10), NC10 and BC10, with number representing OTUs found in each segment (Table S3).

_
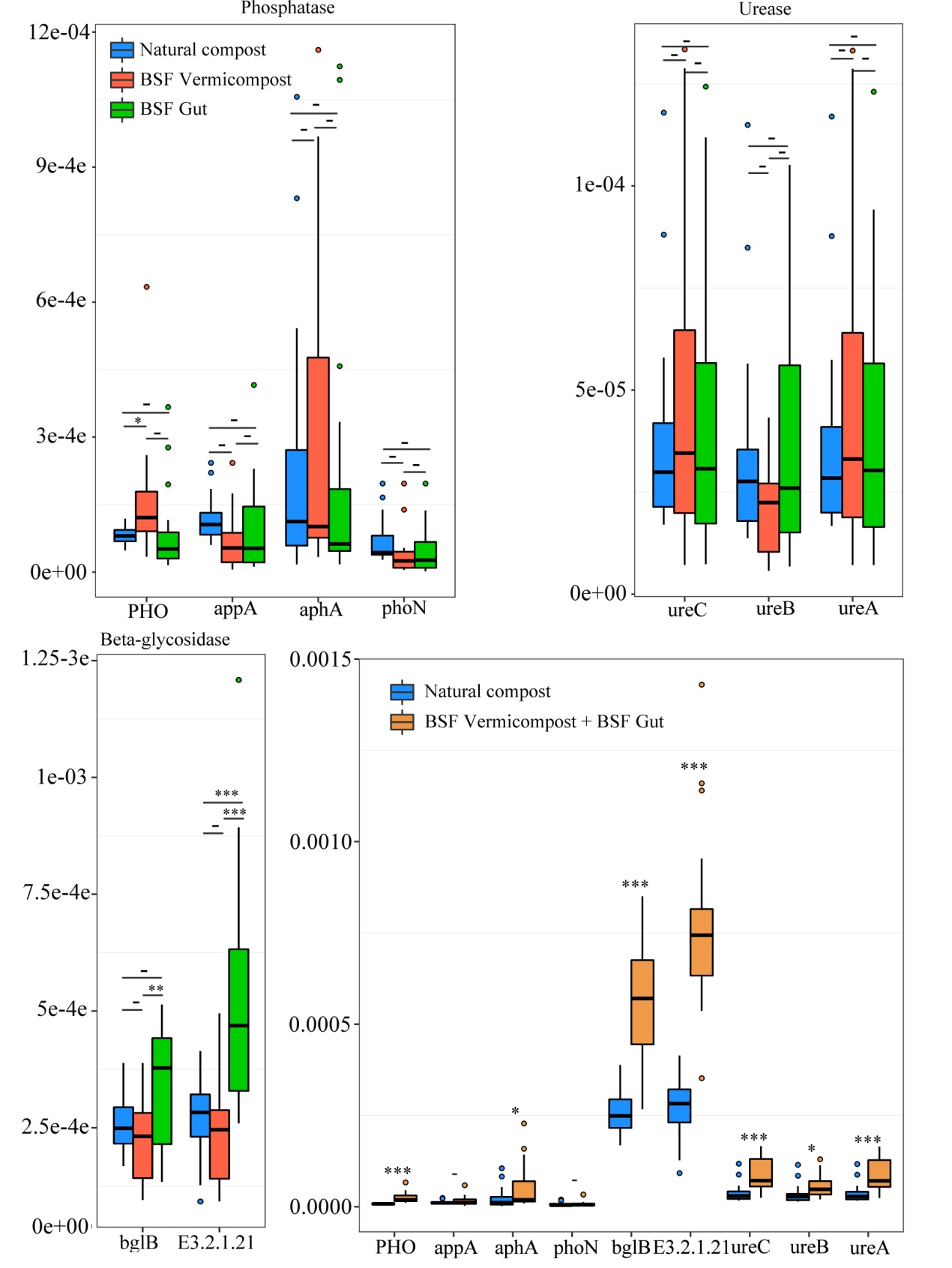
_

**Fig.S6** Box-plots of relative genes abundance about three kinds of enzyme based on K­EGG database. The different color text on the X-axis indicates that the number of genes is higher than that of the other groups (NC:Blue, BC:Orangered, BG:Green, BC+BG:Orange). The circles above or below the box plots indicate Outlier. - *p*>0.05; * *p* < 0.05; ** *p* < 0.01; *** *p* < 0.001.

_
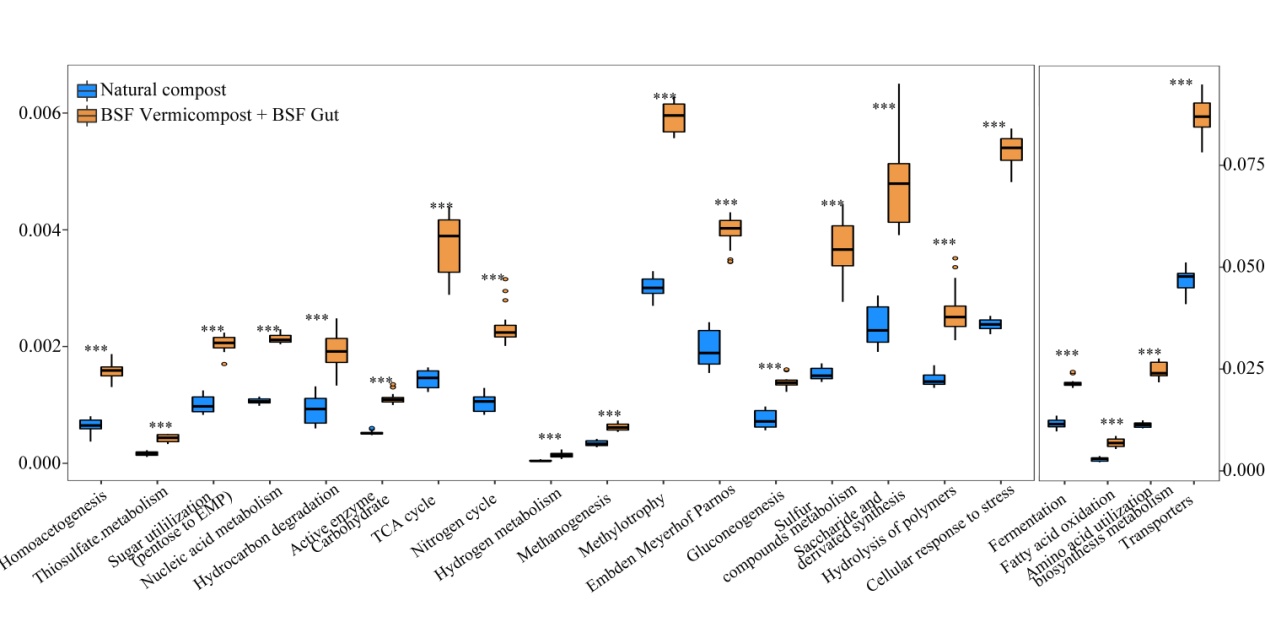

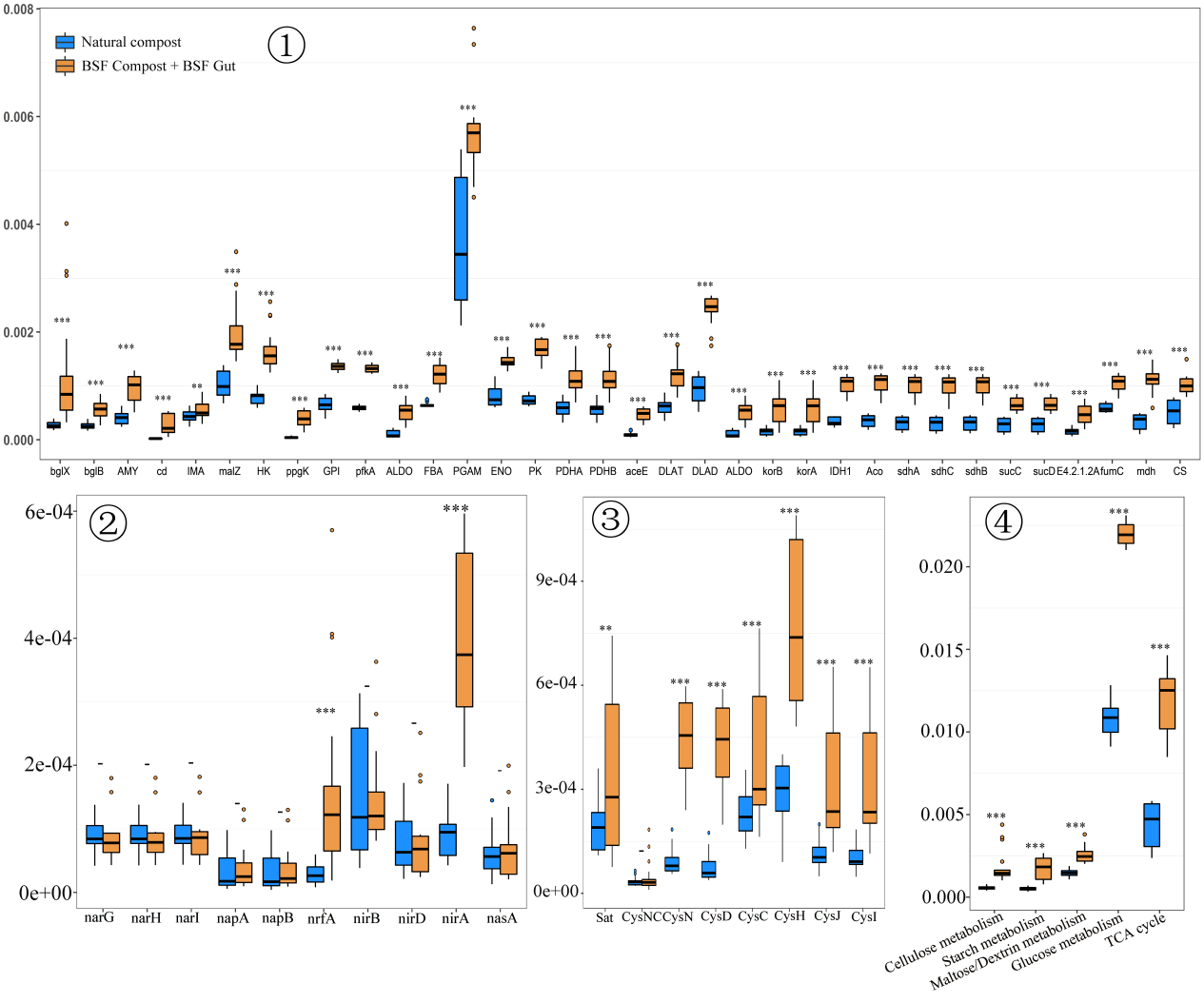
_

B

A

**Fig.S7** A) Box-plots of relative abundance of functional groups based on FOAM database. B) Box-plots of relative abundance of functional genes (B1: Genes are classified according to different steps of carbon metabolism in KEGG, B2: Genes about nitrogen metabolism, B3: Genes about sulfur metabolism, B4: Genes about carbon metabolism).

_
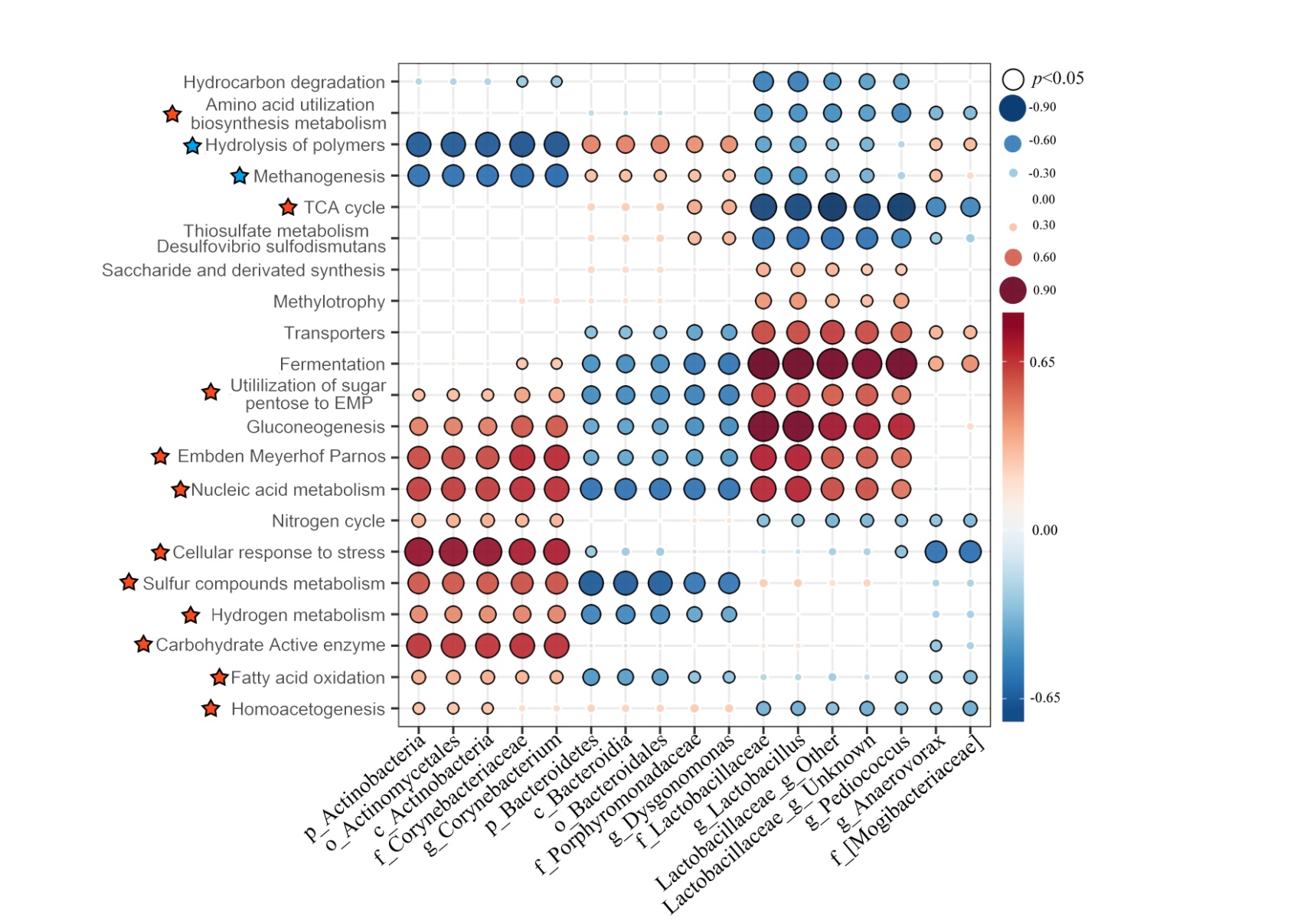
_

**Fig.S8** Spearman rank correlation between metabolic functional group (rows) and bacteria(columns) from Lefse across all groups. Blue and red colors represent positive and negative correlations, respectively. Circle size and color saturation are proportional to the magnitude of the correlation. Statistically significant correlations (*p* <0.05) are indicated by black perimeters. The different color text on the X-axis indicates that bacteria was biomarker in different group (NC:Blue, BC:Orangered, BG:Green). Orangered and blue stars indicate the relative abundance of metabolic functional group in NC are significantly (*p* < 0.05) higher and lower than BC, respectively.


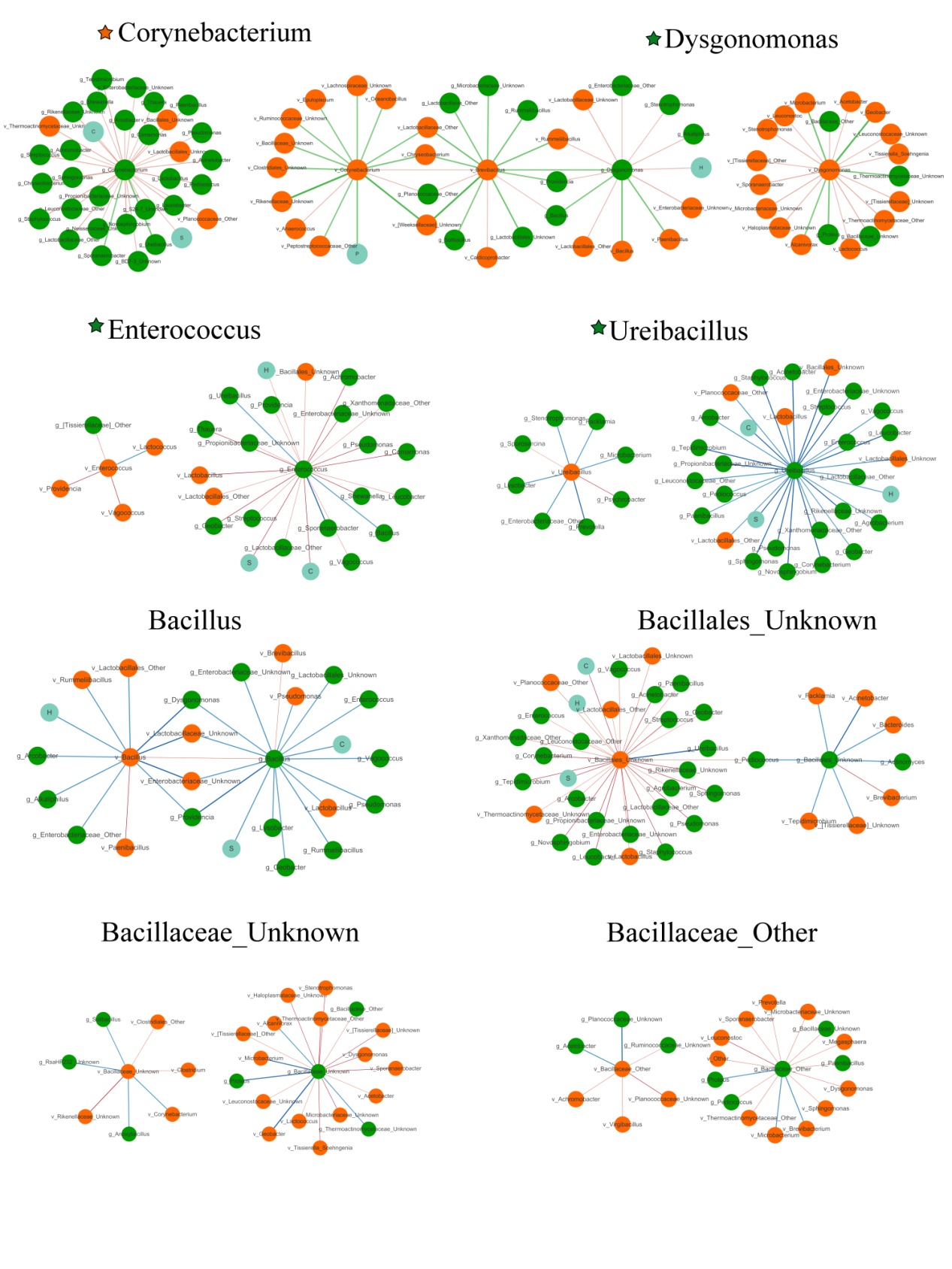


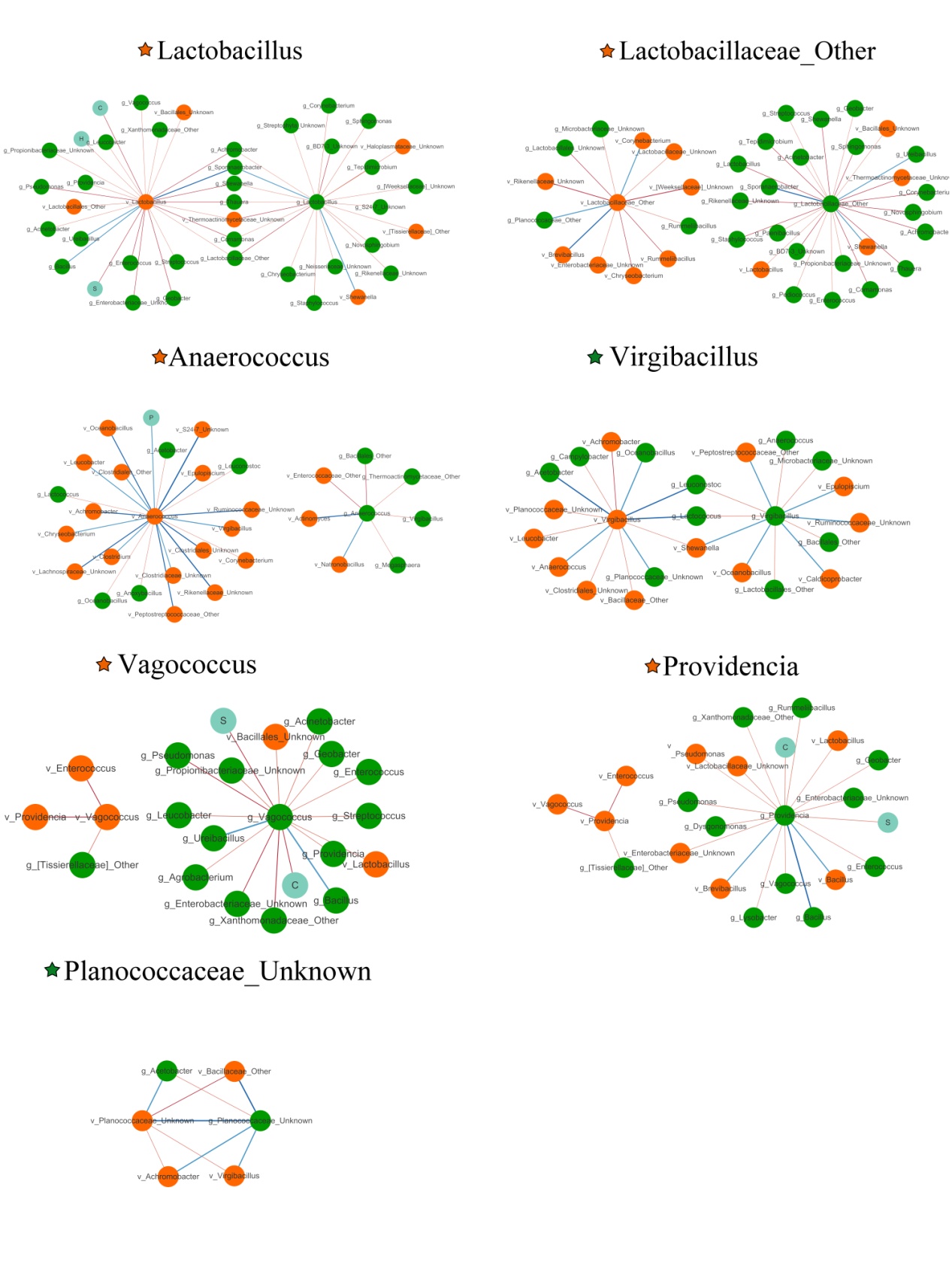


**Fig.S9** Subnetwork organized between the top most abundant bacteria and other genus. The same node colors represent nodes belonging to BG (Green) or BC(Orangered). The thickness of each edge is proportional to the value of the local similarity score. Green and red edges represent positive and negative correlations, respectively. Orangered and green stars indicate the relative abundance of bacteria in BC is significantly (*p* < 0.05) higher or lower than BG.


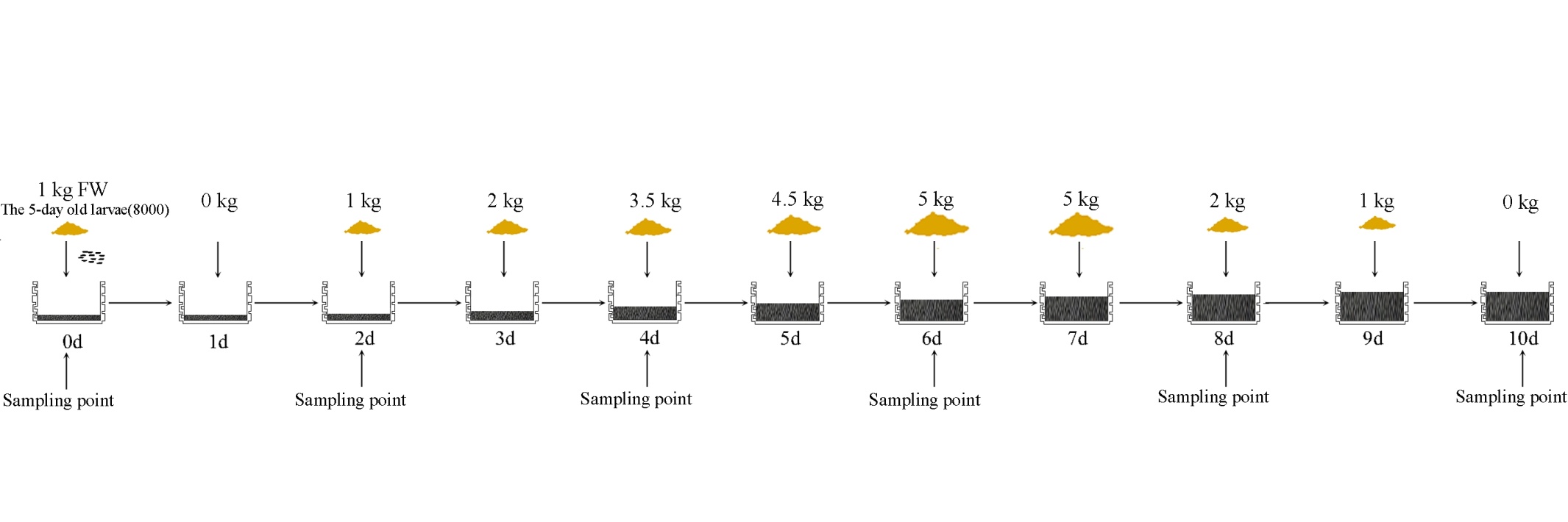


**Fig.S10** A continuous-feeding vermicomposting practice is applied to treat food waste samples with the aid of black soldier fly (*Hermetia illucens L*) larvae. Samples were collected at 6: 00 pm once two day and raw material addition took place at 7:00 pm. Mixing the heap before sampling. Besides, a practice is applied without larvae introduced as controls, of which sampling protocols are completely the same as vermicomposting.

- BSF life cycle

Life cycle of the BSF can be divided into four phases: egg, larva, pupa, and adult stage. The larval stage is 15-30 days, and the time varies according to the external environment, particularly to temperature. During the later period of larval stage (e.g., 12-15 days under room termperature of 25℃) , the prepupae migrates to the dry and suitable pupation site and converts into pupa. The adult flies are neither pest nor disease vector. They survive on the fat stored in their larval stage, later on, they may feed on nothing except water.

- 5 day-old BSF larvae and the amount of BSF larvae (8000) were used in this experiment

According to the operational experience of GuSheng company and previous research([Diener, Solano, Gutiérrez, Zurbrügg, & Tockner, 2011](#_ENREF_3); [Nguyen, Tomberlin, & Vanlaerhoven, 2015](#_ENREF_6); [Stefan, Christian, & Klement, 2009](#_ENREF_9)), we used 5 day-old BSF larvae to treat the food waste in this study. As to the quantity of 5 day-old BSF larvae, we learned from the operational experience of GuSheng company and the article of previous research. Diener's research showed that assuming a larval density of 5 larvae cm^-2^, a prepupal dry mass of 2.5 kg could be harvested for feeding with a waste equivalent of 100 mg chicken feed larva^-1^ •day^-1^([Stefan et al., 2009](#_ENREF_9)).The practice of GuSheng company has already proved that a larval density of 3 larvae cm^-2^, more prepupal dry mass could be harvested when food wates were fed with a waste equivalent of 300 mg FW larva^-1^ •day^-1^(wet weight, 60% moisture, contains 100 mg rice husk powder). Since the size of BSF larvae bioreactor is 40 × 70 × 15 cm, so we decided that the amount of BSF larvae is 8000, and the amount of FW is 25kg.
